# Refinement and Pattern Formation in Neural Circuits by the Interaction of Traveling Waves with Spike-Timing Dependent Plasticity

**DOI:** 10.1371/journal.pcbi.1004422

**Published:** 2015-08-26

**Authors:** James E. M. Bennett, Wyeth Bair

**Affiliations:** 1 Dept. Physiology, Anatomy and Genetics, University of Oxford, Oxford, United Kingdom; 2 Dept. Biological Structure, University of Washington, Seattle, Washington, United States of America; University of Pittsburgh, UNITED STATES

## Abstract

Traveling waves in the developing brain are a prominent source of highly correlated spiking activity that may instruct the refinement of neural circuits. A candidate mechanism for mediating such refinement is spike-timing dependent plasticity (STDP), which translates correlated activity patterns into changes in synaptic strength. To assess the potential of these phenomena to build useful structure in developing neural circuits, we examined the interaction of wave activity with STDP rules in simple, biologically plausible models of spiking neurons. We derive an expression for the synaptic strength dynamics showing that, by mapping the time dependence of STDP into spatial interactions, traveling waves can build periodic synaptic connectivity patterns into feedforward circuits with a broad class of experimentally observed STDP rules. The spatial scale of the connectivity patterns increases with wave speed and STDP time constants. We verify these results with simulations and demonstrate their robustness to likely sources of noise. We show how this pattern formation ability, which is analogous to solutions of reaction-diffusion systems that have been widely applied to biological pattern formation, can be harnessed to instruct the refinement of postsynaptic receptive fields. Our results hold for rich, complex wave patterns in two dimensions and over several orders of magnitude in wave speeds and STDP time constants, and they provide predictions that can be tested under existing experimental paradigms. Our model generalizes across brain areas and STDP rules, allowing broad application to the ubiquitous occurrence of traveling waves and to wave-like activity patterns induced by moving stimuli.

## Author Summary

In several areas of the developing brain, waves of electrical activity trace out distinct patterns across the nervous tissue. These waves are intricately involved in developmental processes that set up the structural connections of the adult brain, but it is unclear what role the wave patterns play. Here, we examine how the strength of connections in these brain areas may change by a process called spike-timing dependent plasticity, which is sensitive to the precise times at which individual neurons become electrically active. We use mathematical models and simulations to show that interactions between waves and plasticity build highly structured patterns into the connections. The results of our model are analogous to many cases of biological pattern formation seen, for example, in zebra stripes, leopard spots and seashells. An important connectivity pattern we consider is the receptive field, which determines to a large extent the specific function of a neuron. We demonstrate how pattern formation can refine the shape of a receptive field and therefore the specificity of a neuron, and explore several ways in which pattern formation may be disrupted, providing clues regarding pathologies in receptive field development. Our theory makes several predictions that may be tested using existing experimental paradigms.

## Introduction

After an initial stage of activity-independent construction [1], the developing nervous system undergoes a period of refinement that is strongly influenced by spontaneous and evoked patterns of neural activity [2, 3]. Traveling wavefronts are a striking feature of these activity patterns [4–9]. Within short temporal windows, wavefronts induce strong interneuronal correlations that can act through Hebbian mechanisms of synaptic plasticity to build structure into the connectivity of neural circuits [10, 11]. This has prompted the hypothesis that correlated activity plays an instructive role for circuit refinement in the developing brain (reviewed in [2] and [3]). One Hebbian mechanism that is well suited to this role and is widely reported in the brain is spike-timing dependent plasticity (STDP), for which synaptic connections are strengthened or weakened depending on the relative timing of pre- and postsynaptic spikes that arrive at the synapse, typically within tens of milliseconds of each other [12–14]. In this article, we undertake a mathematical analysis of the interaction between traveling wave activity patterns and STDP, and explore the types of connectivity patterns that emerge as a result of this interaction.

Past studies have demonstrated that STDP could translate correlated input patterns into structured neural circuits [15–21], and could mediate the construction of realistic receptive fields (RFs) with properties that resemble those found in the visual cortex [22, 23]. These models primarily focussed on spatial and temporal correlations as separable features when considering their interaction with STDP. However, the spatiotemporal correlations induced by traveling waves are space-time inseparable, providing additional information that may be utilized during circuit building. Space-time inseparable activity patterns map the temporal profile of the STDP rule into a spatial profile of synaptic strength changes [24], which can be used to build circuits that mimic neuronal sensitivity to visual motion during repeated exposure to moving visual stimuli [25–28]. But despite the demonstrated applicability of STDP to specific cases of neural circuit development, a more general, formal analysis of the interaction of STDP and wave-like activity patterns is still lacking.

Here, we derive a mathematical expression that accounts for the interaction of a variety of traveling wave activity patterns and STDP rules, and we examine the analytical predictions in a simple yet biologically plausible model of spiking neurons. We show that, for a broad class of experimentally observed STDP rules, such interactions build highly structured and periodic connectivity patterns into feedforward circuits, analogous to a Turing instability in reaction diffusion systems [29], which has been applied to diverse cases of biological pattern formation [30, 31]. We then demonstrate the robustness of this pattern formation process and how it can be utilized to construct and refine the size and shape of RFs. Our results offer theoretical insights that may advance the understanding of the role played by traveling wave phenomena in different areas of the brain. We highlight particular insights into visual system development and outline a number of predictions that may be tested experimentally.

## Results

Our results are organized as follows. First, we describe how traveling waves can interact with STDP to influence synaptic strengths, derive a mathematical expression for this effect and verify analytical solutions to the derived equations using a simulation of spiking neurons. Second, we use further simulations to explore the robustness of our analytical results. Third, we demonstrate the properties of waves and STDP rules that allow for different types of refinement in downstream receptive fields (RFs).

### Illustration of network dynamics

**Fig 1:**
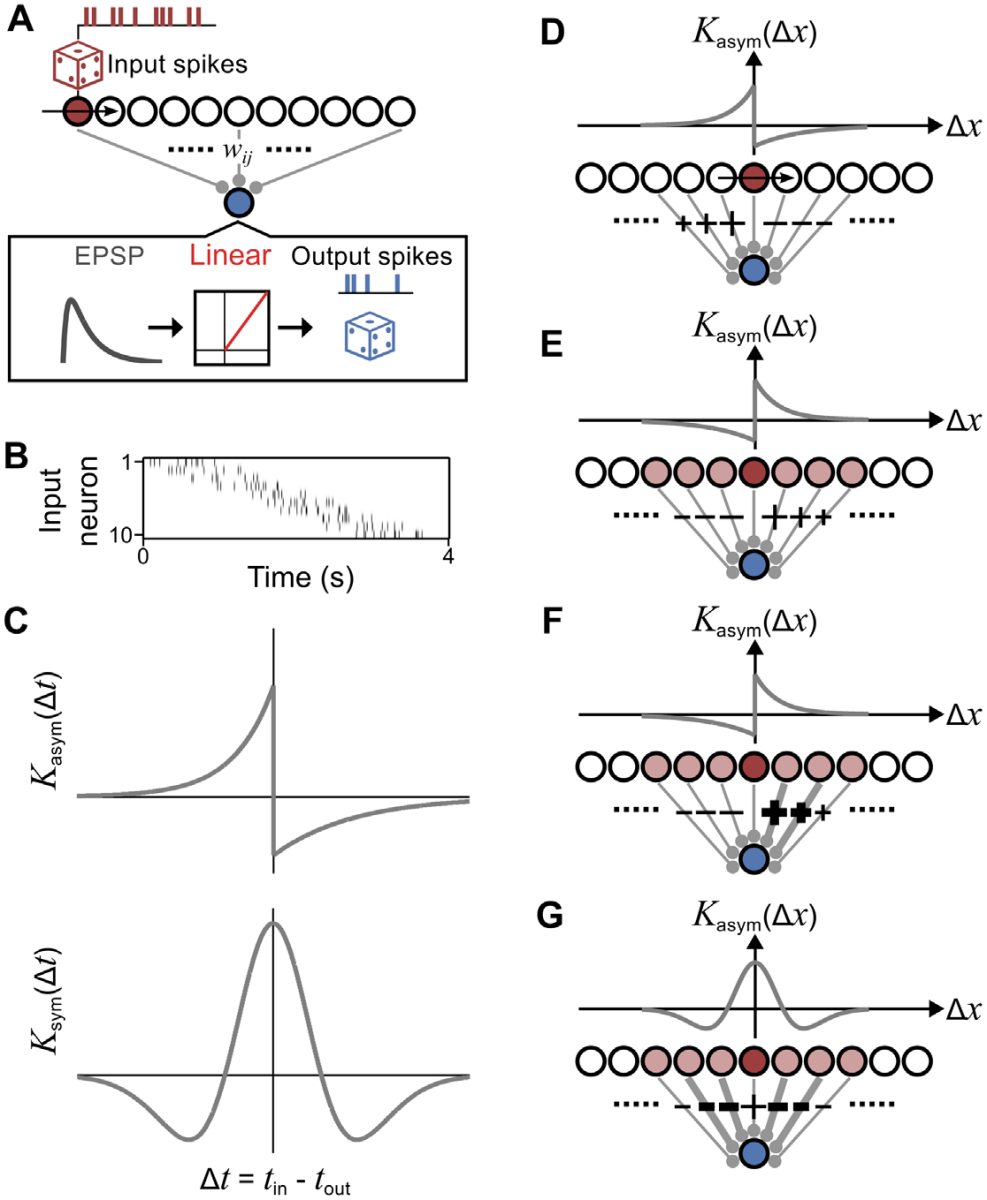
Model framework and dynamics. **A**. The model consists of a layer of input neurons, along which wavefronts of spiking activity propagate. Input neurons undergo a burst of spikes, generated stochastically, when the wavefront passes (red unit). Input spikes elicit excitatory postsynaptic potentials (EPSPs) in the output neuron (blue unit) that are scaled by the synaptic strength, *w*_*ij*_. The output neuron generates spikes stochastically with a firing rate linearly proportional to its summed EPSP. **B**. Example spike raster from the simulation, showing one wave traveling past input neurons 1–10. **C**. Synaptic strengths are modified by either an asymmetric (top) or symmetric (bottom) STDP rule. **D**. Schematic for the influence of the wavefront on modifications at surrounding synapses. An input neuron (red) is recruited by a wave, which travels from left to right, and increases the firing rate of the output neuron (blue). When an asymmetric STDP rule is at play, output spikes at the current time point will cause synapses behind the wavefront to increase in strength (‘+’ symbol) because they were active at an earlier time. Similarly, synapses in front of the wave will decrease (‘−’ symbol), because they will become active at a later time. Thus, traveling waves map the STDP rule onto space. **E.** Schematic for the influence of surrounding inputs (colored light red), on synaptic modifications at the wavefront (dark red). **F**. Same as **E**, except that inputs to the right of the wavefront induce even greater synaptic strengthening as their respective synapses are stronger. **G**. Same as **E**, except for a symmetric STDP rule. Here, the greater strength of synapses either side of the wavefront induce more synaptic weakening.

To understand how correlated activity caused by traveling waves could influence synaptic strengths via a STDP mechanism, we consider a reduced model (Fig 1A) consisting of a one-dimensional (1D) layer of presynaptic input cells, all of which connect via excitatory synapses, *w*_*i*,*j*_, onto a single, postsynaptic output cell. In later sections, we extend the input layer to two dimensions. When a wave traverses the input population (Fig 1A), each input neuron is recruited by the wavefront (red colored unit with rightward arrow) and discharges a burst of spikes, which drives spiking in the output neuron. Temporal differences between the spike times of a given input neuron and the output neuron, Δ*t* = *t*_in_−*t*_out_, determine how the respective synapse is modified in strength according to a STDP rule, *K*(Δ*t*) (see Methods, Eqs 21 and 22). The set of spikes for all input neurons leads to a simple diagonal band structure in space and time (Fig 1B).

To illustrate how this input spike pattern leads to spatially structured changes in synaptic strength, we consider a STDP rule that is asymmetric in Δ*t* (Fig 1C, top). In Fig 1D, we show a snapshot of the traveling wave and its influence on the surrounding synaptic strengths. Here, an input neuron (colored dark red) is recruited by the wavefront and fires a burst of spikes, which in turn elicits excitatory postsynaptic potentials (EPSPs) that drive the output neuron to spike. For waves traveling left to right, spikes generated by input neurons to the left of the wavefront always precede output spikes. Consequently, their respective synapses are strengthened, because the STDP rule specifies strengthening for negative Δ*t*. Likewise, synapses connecting input neurons to the right of the wavefront will be weakened. In fact, due to the wave motion, the dependence of the STDP rule on relative spike times, Δ*t*, is mapped onto the spatial axis of the input layer, Δ*x*, and thus the relative spike locations, as shown in Fig 1D and 1G. Consequently, as depicted in Fig 1E, all input neurons (colored light red) that surround a synapse will influence the net change in strength at that synapse. One might predict this change to be proportional to the integral of the STDP rule, $K_{0}=\int_{-\infty }^{\infty }d\Delta t\;K(\Delta t)$. However, the extent to which an input influences surrounding synapses depends on its influence over the output firing rate, and thus its own synaptic strength. For example, with an asymmetric STDP rule and waves traveling left to right, a synapse will strengthen relative to *K*_0_ if the surrounding synapses are stronger to the right than to the left, as in Fig 1F. On the other hand, a synapse will be relatively weakened if the surrounding synapses are stronger to the left than to the right. By the same argument, a synapse will be relatively weakened by a temporally symmetric STDP rule if the surrounding synapses are stronger on both sides (Fig 1G), and relatively strengthened if the surrounding synapses are weaker. In this way, strong synapses increasingly dictate changes to local synaptic strengths as more waves pass. Eventually, the connectivity pattern begins to form islands of strong synapses that are flanked by regions of weak synapses.

In the following analytical derivation and simulations, we show how this process of wave-induced STDP results in a distinct type of pattern formation in the network for a broad class of STDP rules.

### Deriving synaptic strength dynamics

Here, we derive a description for the dynamics of synaptic strengths that are driven by pairs of input and output spikes acting through a STDP rule and resulting from traveling wave activity patterns traversing the input layer. Within our framework, input and output spike trains are generated by a stochastic process with a time-dependent firing rate. The stochastic arrival of spikes results in stochastic changes to the synaptic strengths, thus posing a challenge when seeking a description for the spatial structure of synaptic strengths that evolves slowly over long periods of time, during which many traveling waves occur. It is therefore useful to separate the slow dynamics from the fast, stochastic dynamics under the assumption that, during a limited period of time, Δ*T*, individual changes in synaptic strengths are negligible, but accumulate slowly over multiple periods of Δ*T* as a result of the time-averaged input and output activity. By approximating synaptic strengths as being constant during the period Δ*T*, Kempter et al. [16] showed that changes in synaptic strength over this period could be described by the inner product of the STDP rule, *K*(Δ*t*), and the cross-correlation function, *C*_*ij*_(Δ*t*,*t*), between the spike trains of input neuron *i* and output neuron *j*:

$$\frac{w_{ij}\left(t+\Delta T\right)-w_{ij}\left(t\right)}{\Delta T}=\frac{\Delta w_{ij}\left(t\right)}{\Delta T}\approx \eta \int_{t}^{t+\Delta T}\;d\Delta t\;K\left(\Delta t\right)C_{ij}\left(\Delta t,t\right),$$ (1)

where *η* is a small, positive constant that sets the required slow rate of change in synaptic strengths. The cross-correlation is given by:

$$C_{ij}\left(\Delta t,t\right)=\int_{t}^{t+\Delta T}\;dt^{\prime }\;S_{i}\left(t^{\prime }+\Delta t\right)S_{j}\left(t^{\prime }\right),$$ (2)

where *S*_*i*(*j*)_(*t*) are ensemble averages, for example over multiple waves, of the input (output) spike trains and can thus be identified with the input (output) firing rates. It is important that the firing rates be sufficiently high for *C*_*ij*_(Δ*t*,*t*) to accurately portray wave-induced correlations. In addition, *η* must be sufficiently small for wave-induced correlations to be recovered over several waves. Moreover, without small *η*, calculating *C*_*ij*_(Δ*t*,*t*) becomes difficult, as *S*_*j*_(*t*) would depend on stochastically changing synaptic strengths, *w*_*ij*_(*t*). As such, Eq 1 implements the approximation by averaging over the small stochastic fluctuations, thus providing only the mean drift in *w*_*ij*_(*t*). Given that we will deal with discrete waves that pass one-by-one across the input layer, it is convenient to relate the time scale, Δ*T*, to the passage time of just a single wave. Further assumptions are now required to uphold the validity of Eq 1. First, in order to ensure that multiple waves do not mutually influence changes in synaptic strength, Δ*T* must include an amount of time, 𝓚, both before and after the wave, where 𝓚 is the temporal width of the STDP rule which contains most of its power. More formally, we require that $\int_{-𝓚}^{𝓚}d\Delta t\;\text{∣K(Δt)∣}\gg \int_{-\infty }^{-𝓚}d\Delta t\;\text{∣K(Δt)∣}+\int_{𝓚}^{\infty }d\Delta t\;\text{∣K(Δt)∣}$ [16]. Because we are effectively considering Δ*w*_*ij*_(*t*) for a wave in isolation, we can extend the integral limits in Eqs 1 and 2 to ±∞. Second, with *w*_*ij*_(*t*) effectively constant during a wave, and because Δ*w*_*ij*_(*t*) is small, we will analyze changes in *w*_*ij*_ on a slower time scale, *T*, which is discretized in Δ*T* increments. Thus, we approximate *w*_*ij*_(*t*) with *w*_*ij*_(*T*) and *C*_*ij*_(Δ*t*,*t*) with *C*_*ij*_(Δ*t*,*T*), and therefore approximate the left-hand side of Eq 1 with ∂*w*_*ij*_(*T*)/∂*T* = ∂_*T*_ *w*_*ij*_(*T*).

By considering a simple 1D chain of input neurons with a single output neuron, we replace all subscripts *i* with the argument, *x*. That is, we replace *w*_*ij*_(*T*) with *w*(*x*,*T*), *S*_*i*_(*t*) with *S*_in_(*x*,*t*), and *C*_*ij*_(Δ*t*,*T*) with *C*(*x*,Δ*t*,*T*). Eq 1 then becomes:

$$\partial_{T}w\left(x,T\right)\approx \eta \int_{-\infty }^{\infty }\;d\Delta t\;K\left(\Delta t\right)C\left(x,\Delta t,T\right),$$ (3)

where $C(x,\Delta t,T)=\int_{-\infty }^{\infty }\;dt\;S_{\text{in}}(x,t+\Delta t)S_{\text{out}}(t)$, with *S*_in_ and *S*_out_ the input and output firing rates, respectively. As a final matter of notation, we will hereafter refrain from explicitly writing the dependence of *w* and *C* on *T*, for brevity.

We make two further assumptions to simplify the analytical derivation, then relax these for a more general case: *i*) the input firing rate at the wavefront can be described as a short, traveling pulse using a Dirac delta function: *S*_in_(*x*,*t*) = *δ*(*x*−*vt*), where *δ*(*y*) = ∞ if *y* = 0 and is zero otherwise, and *v* is the wave speed; *ii*) the output neuron’s response to its input is instantaneous: $S_{\text{out}}(t)=\int_{-\infty }^{\infty }\;dx\;S_{\text{in}}(x,t)w(x)=w(vt)$. Using these forms for *S*_(in)out_, *C*(*x*,Δ*t*) = *w*(*x*−*v*Δ*t*) and we can write Eq 3 as

$$\begin{array}{ccc} \partial_{T}w\left(x\right) & = & \eta \int_{-\infty }^{\infty }\;d\Delta t\;K\left(\Delta t\right)w\left(x-v\Delta t\right)\\ & = & \frac{\eta }{v}\int_{-\infty }^{\infty }\;d\Delta x\;K\left(\Delta x/v\right)w\left(x-\Delta x\right)\\ & = & \eta K_{v}\left(x\right)*w\left(x\right), \end{array}$$ (4)

where *K*_*v*_(*x*) has been introduced as a rescaled copy of *K*, *K*_*v*_(*x*) = *v* ^−1^ *K*(*x*/*v*), and * denotes convolution. There are two key features to Eq 4. Firstly, as illustrated above, the STDP rule can be reinterpreted as a spatial kernel as a result of the wavefront’s constant velocity, which maintains a strict relationship between space and time. Secondly, the wave’s effect on the synaptic dynamics is described by a convolution of the STDP rule with the synaptic strengths. By deriving a solution for *w*(*x*) in Eq 4, we will demonstrate how convolution plays an important role in the type of connectivity patterns that *w*(*x*) acquires, but first we relax the two simplifying assumptions used to reach Eq 4.

Incorporating finite input bursts and the dependence of output firing rates on EPSPs adds a simple modification to Eq 4, which becomes

$$\partial_{T}w\left(x\right)=\eta K_{v}\left(x\right)*\alpha \left(-x/v\right)*\alpha \left(x/v\right)*\epsilon \left(x/v\right)*w\left(x\right),$$ (5)

where *α*(*t*) describes the time dependent firing rate during an input burst, and thus captures the shape of the wavefront, and *ϵ*(*t*) is the EPSP. In our model, both *α*(*t*) and *ϵ*(*t*) are positive valued for *t* > 0 (Methods), and act as low pass filters on the STDP rule. The full derivation for Eq 5 is provided in S1 Text. Note that the firing rate of the output neuron (*R*_out_ in Methods) acts simply as a coefficient of *ϵ* in Eq 5 and hence plays a similar role to *η* by varying the rate at which synaptic strengths are modified. Amalgamating all terms in Eq 5, except for *w*(*x*), we have

$$\partial_{T}w\left(x\right)=\eta \kappa \left(x\right)*w\left(x\right),$$ (6)

where *κ*(*x*) is the effective spike-location dependent plasticity rule, which incorporates the dynamics of input bursts and EPSPs:

$$\kappa \left(x\right)=K_{v}\left(x\right)*\alpha \left(-x/v\right)*\alpha \left(x/v\right)*\epsilon \left(x/v\right).$$ (7)

**Fig 2:**
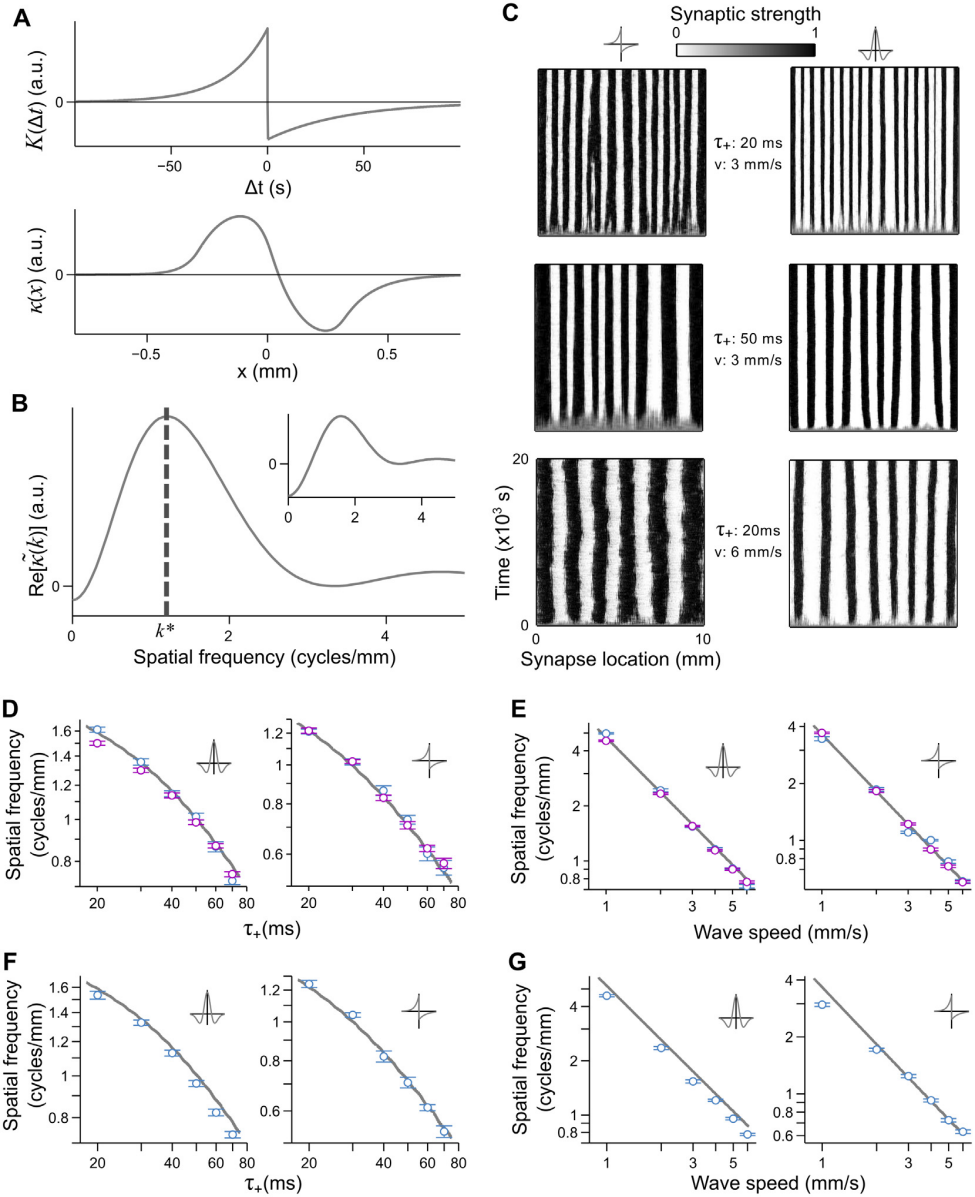
Traveling waves build periodic connectivity patterns into the synaptic strengths through STDP. **A**. Example of a STDP rule (top panel, *τ*_+_ = 20 ms, *τ*_−_ = 40 ms, *A*_+_ = 1.0, *A*_−_ = 0.51) that is mapped into a location-dependent plasticity rule (bottom panel) by a wave traveling at 3 mm/s (burst duration is 0.1 s). **B**. Re[$\tilde{\kappa }(k)$] for the STDP rule in **A**. The dominant spatial frequency, k*, lies where $\tilde{\kappa }(k)$ has a global maximum. Inset: $\tilde{\kappa }(k)$ for a symmetric STDP rule (*τ*_+_ = 20 ms, *v* = 3 mm/s). **C**. Evolution of the synaptic strengths, initialized with strength 0.5, from 500 input neurons to a single output neuron, under the influence of an asymmetric (left column) and symmetric (right column) STDP rule. Waves sculpt a periodic connectivity pattern into the synaptic strengths with a spatial frequency equal to k* (see **B** and Eq 5). Larger values for *τ*_+_ (controls STDP rule width) or *v* (wave speed) produce lower spatial frequencies in the periodic pattern. Values for *τ*_+_ and *v* are given between the columns. **D–E**. Log-log plots for the spatial frequency of the steady state connectivity pattern as a function of *τ*_+_ and *v*, for both the symmetric (left panels) and asymmetric (right panels) STDP rules. Grey curves: predicted k*; blue: mean spatial frequency ± SEM of the final connectivity pattern in the spiking simulation; purple: mean spatial frequency ± SEM of *w*(*x*) in the numerical solution to Eq 6. **D**. Dependence of the dominant spatial frequency on *τ*_+_, keeping *v* = 3 mm/s fixed. **E**. Dependence of the dominant spatial frequency on *v*, keeping *τ*_+_ = 20 ms fixed. **F–G**. Same as **D** and **E**, but for an integrate and fire output neuron model.

When a typical STDP rule (Fig 2A, top; see legend for parameters) is transformed into a spatial kernel (Fig 2A, bottom) by a wave traveling at 3 mm/s (the speed of spontaneous waves in the mouse cerebellum [8], or a 25 °/*s* stimulus on the kitten retina using the visual angle to space conversion in Methods), the kernel extends over approximately 1 mm of input space. Note that *κ*(*x*) preserves the asymmetric shape of the STDP rule, but is low pass filtered by *α*(*t*) and *ϵ*(*t*).

A solution for *w*(*x*) as *T* → ∞ is more easily found in the frequency domain by taking the Fourier transform, such that convolution becomes multiplication:

$$\partial_{T}\tilde{w}\left(k\right)=\eta \tilde{\kappa }\left(k\right)\tilde{w}\left(k\right),$$ (8)

where ‘∼’ denotes the Fourier transform and *k* is the spatial frequency. Using *w*_0_ to describe the initial synaptic strengths at time *T* = 0, the solution to Eq 8 for $\tilde{w}(k)$ is

$$\tilde{w}\left(k\right)={\tilde{w}}_{0}\left(k\right)e^{\eta \tilde{\kappa }\left(k\right)T}.$$ (9)

We can reconcile Eq 9 with several previous studies of STDP by writing $\tilde{\kappa }(k)=\lambda (k)+i\phi (k)$ to explicitly express real and imaginary components:

$$\tilde{w}\left(k\right)={\tilde{w}}_{0}\left(k\right)e^{\eta \lambda (k)T}e^{i\eta \phi (k)T}.$$ (10)

Here, the imaginary component gives rise to a spatial phase shift in *w*(*x*), a feature that has been exploited to drive a spatial redistribution of synaptic strengths by asymmetric STDP rules in several models of sequence learning [26, 32], asymmetric shifts in hippocampal place fields [33, 34], and the formation of direction selective cells [24, 27, 28].

The focus of our results, however, is the stability of $\tilde{w}(k)$, which is determined by the real component, *λ*(*k*). We therefore consider waves that travel in both directions so that, for sufficiently small *η*, *κ*(*x*) is effectively symmetric and contains no imaginary component. This can be shown by integrating Eq 8 over two wave events, with the first wave traveling left to right, and the second wave traveling right to left (replacing *v* with −*v*). After the first wave, at *T* = *T*_1_,

$$\tilde{w}\left(k,T_{1}\right)={\tilde{w}}_{0}\left(k\right)e^{\eta \tilde{\kappa }\left(k\right)T_{1}}.$$ (11)

Using $\tilde{w}(k,T_{1})$ as the initial condition for the second wave, we have at *T* = *T*_2_

$$\tilde{w}\left(k,T_{2}\right)=\tilde{w}\left(k,T_{1}\right)e^{\eta \bar{\tilde{\kappa }\left(k\right)}\left(T_{2}-T_{1}\right)},$$ (12)

where $\bar{\tilde{\kappa }(k)}$ is the complex conjugate of $\tilde{\kappa }(k)$, with conjugation resulting from the sign reversal in the wave speed. Expanding Eq 12, we have

$$\begin{array}{ccc} \tilde{w}\left(k,T_{2}\right) & = & {\tilde{w}}_{0}\left(k\right)e^{\eta \lambda \left(k\right)T_{1}}e^{i\eta \phi \left(k\right)T_{1}}e^{\eta \lambda \left(k\right)\left(T_{2}-T_{1}\right)}e^{-i\eta \phi \left(k\right)\left(T_{2}-T_{1}\right)}\\ & = & {\tilde{w}}_{0}\left(k\right)e^{\eta \lambda (k)2\Delta T}, \end{array}$$ (13)

where Δ*T* = *T*_1_ = *T*_2_−*T*_1_ is the time taken for one wave to cross the input layer. We can therefore write an approximation to Eq 9 for the special case in which waves travel in both directions in equal numbers:

$$\tilde{w}\left(k\right)\approx {\tilde{w}}_{0}\left(k\right)e^{\eta \text{Re}[\tilde{\kappa }\left(k\right)]T}.$$ (14)

If the wave direction were random, instead of alternating, Eq 14 would be valid only if a large number of waves traversed the input layer during the interval Δ*T*/*η*.

Thus, for any *k* such that $\text{Re}[\tilde{\kappa }(k)]<0$, $\tilde{w}(k)$ will be stable and decay to zero, whereas for any *k* such that $\text{Re}[\tilde{\kappa }(k)]>0$, $\tilde{w}(k)$ will become unstable and grow exponentially. For the STDP rules used in this study (Fig 1C), $\tilde{\kappa }(k)$ has a positive valued maximum at k* (Fig 2B). Therefore, if the synaptic strengths are initially random, such that the expected form of ${\tilde{w}}_{0}(k)$ is flat, then $\tilde{w}(k^{*})$ will be the fastest growing eigenmode of the synaptic strengths, and $\tilde{w}(k)$ will asymptotically approximate a delta function, *δ*(k*). If only a single spatial frequency dominates $\tilde{w}(k)$, *w*(*x*) will be sinusoidal. Thus, our derivation predicts that, for STDP rules like those reported experimentally, the synaptic strengths will develop a connectivity pattern that is periodic in space with a period of 1/k*, under the influence of traveling waves.

A similar model of pattern formation describes the development of ocular dominance columns in primary visual cortex [35], in which it is proposed that short range excitatory and long range inhibitory lateral interactions give rise to the spatially periodic dominance of eye specific afferents across primary visual cortex. In our model, it is the mapping of the STDP rule onto space that provides the lateral interactions necessary for pattern formation. More generally, the solution derived above is analogous to pattern forming solutions that result from Turing instabilities in reaction-diffusion systems [29], whereby the initially homogeneous distribution of synaptic strengths becomes unstable, allowing the fastest growing eigenmode to dominate the resulting pattern. In systems composed of a diffusible activator and an inhibitor, Turing instabilities frequently occur when the inhibitor diffuses over greater distances than does the activator [30]. Here, the decay constants of the positive and negative STDP lobes bear similarities to the length scales of diffusion in reaction-diffusion systems. Thus, STDP rules with narrow windows for strengthening and wide windows for weakening are good candidates for pattern formation in neural circuits that support traveling wave activity patterns.

### Stability of the connectivity pattern

If the synapses are unbounded, the STDP rules used here will always yield a sinusoidal connectivity pattern when driven by traveling waves, given sufficient time, and the mean synaptic strength will always be zero (the mean of a sine wave). This is far from a physiologically plausible scenario: without bounds, the synaptic strengths would tend towards ±∞. When bounds are imposed, however, care is needed to formulate the STDP rule, so that the synaptic strengths do not reach the upper or lower bound before the dominant eigenmode at *k* = k* takes hold. This can be achieved by ensuring that there is not too strong a bias for either synaptic weakening or strengthening in the STDP rule: $-B_{L}<\int_{-\infty }^{\infty }d\Delta t\;K(\Delta t)=\text{Re}[\tilde{\kappa }(k=0)]<B_{U}$, where *B*_*L*_ and *B*_*U*_ are, respectively, the magnitudes of the lower and upper bounds to the STDP bias. Because synaptic strengths are constrained to be positive, it must be that *B*_*L*_ > *B*_*U*_. In other words, the range of biases for synaptic weakening that will yield stable periodic patterns is greater than that for strengthening. Nevertheless, it is important to note that we need not have *B*_*U*_ < 0. This contrasts with previous studies that utilized different input activity patterns [16, 17, 19], and that required a bias for synaptic weakening by setting $\int_{-\infty }^{\infty }d\Delta t\;K(\Delta t)<0$ to stabilize the connectivity pattern. That is, in our model, it remains possible for periodic patterns to emerge even if there is a bias for synaptic strengthening in the STDP rule, so long as the synaptic strengths are not pushed to the upper bound before the dominant spatial frequency takes hold. The stability of the connectivity will also be sensitive to the learning rate, which is scaled by *η* in Eq 6, and the initial conditions of the synaptic strengths. For example, we later explore formulations of $\tilde{\kappa }(k)$ for which there are multiple peaks that are similar in amplitude, or a single, broad peak. The stochastic dynamics introduced by spiking neurons may therefore move the slower dynamics of plasticity along a spectrum of eigenmodes, resulting in more aperiodic connectivity patterns. We later introduce a robustness measure to quantify the periodicity of a connectivity pattern and that takes these potential features of $\tilde{\kappa }(k)$ into account.

### Spatial pattern formation in a spiking neuron simulation

To test the predictions of the analytical derivation above, we examined whether a periodic connectivity pattern would develop as a result of traveling waves and STDP in a simulated network of linear Poisson, spiking neurons. The simulation captures the architecture and function of the network illustrated in Fig 1A, consisting of a 1D layer of input neurons that all synapse onto a single output neuron. During simulations, synaptic strengths were modified by one of two distinct forms of STDP rule (see Methods) that have been reported in the literature, one that is asymmetric (Eq 21) [13, 17, 36] and the other symmetric (Eq 22) [37, 38] in Δ*t*. Spiking activity is generated by a traveling wavefront that moves back and forth along the input layer, eliciting EPSPs and spikes in the output neuron. We varied two parameters in the simulation: *i*) the temporal window of *K*(Δ*t*), which we control with the decay time constant for synaptic strengthening, *τ*_+_, and *ii*) the wave speed, *v*. These parameters modulate the shape of *κ*(*x*) and, therefore, the spatial frequency of the predicted periodic connectivity pattern.

As predicted by our derivation (Eq 9), the synaptic strengths in the simulation indeed developed a periodic connectivity pattern (Fig 2C) in the presence of traveling waves for both the asymmetric and symmetric rules. The periodic pattern was robust over a range of STDP time constants (*τ*_+_ = 20, 30, 40, 50, 60 and 70 ms), keeping the wave speed constant (*v* = 3 mm/s), and for a range of wave speeds (*v* = 1, 2, 3, 4, 5, and 6 mm/s), keeping *τ*_+_ constant (*τ*_+_ = 20 ms). As predicted, increasing *τ*_+_ or *v* caused the period of the connectivity pattern to increase (Fig 2C). To test the accuracy with which Eq 9 predicts the resulting spatial frequency over this range of parameters, we computed the power spectrum of the steady state synaptic strengths (mean subtracted) to which a Gaussian curve was fit. The spatial frequency of the connectivity pattern was taken to be the centre of the fitted Gaussian. We repeated simulations sixteen times for each set of parameters, using a different seed for the random number generators. In Fig 2D and 2E, we compare the measured spatial frequencies (blue circles) with the predicted spatial frequencies, k* (grey curves), as a function of *τ*_+_ (Fig 2D) and *v* (Fig 2E), where the predicted values were found by numerically computing $\text{Re}[\tilde{\kappa }(k)]$ and determining the spatial frequency at its peak. We measured the accuracy of our predictions by computing the coefficient of determination, R^2^, between the logarithms of the predicted and measured spatial frequencies. For each panel in Fig 2D and 2E, R^2^ > 0.85. Thus, the assumptions made to derive Eq 9 appear to maintain a veritable description of the noisy dynamics in the simulation over the range of parameters tested. In addition to the simulations, we verified that solutions obtained by numerically integrating Eq 6 (incorporating nonlinearities such as hard bounds to *w*(*x*), see Methods) also produced periodic connectivity patterns with spatial frequencies that matched predictions (magenta circles, Fig 2D and 2E, R^2^ > 0.92).

To investigate whether nonlinearities in the postsynaptic response influence the outcome of the connectivity patterns, we replaced the linear output neuron with a leaky integrate and fire (LIF) neuron (see Methods) that modeled absolute and relative refractory periods of 2 ms and 5 ms, respectively. These simulations yielded a similar relationship between the spatial frequency of the connectivity pattern, wave speed and STDP time scale (Fig 2F and 2G). This similarity might be expected, as the wave correlations extend over relatively long time scales and thus smooth out any ripples in the correlation function, *C*(*x*,Δ*t*), that would be introduced on the short time scales of the refractory period or EPSP rise time. More noticeable differences may, however, be observed if wavefronts elicited very short bursts or only single spikes.

The development of periodic patterns in synaptic connectivity could have wide applications throughout the nervous system of many species, particularly because of the ubiquity of both traveling waves and STDP. However, it is first necessary to determine the extent to which pattern formation is influenced 1) over the range of space and time scales observed in biology, 2) in the presence of noise, and 3) by 2D waves. We explore these issues in the following sections.

### Pattern formation over a wide range of spatial and temporal scales

In the previous section, we found that the wave speed and STDP time scale are key parameters that determine the spatial frequency of periodic connectivity patterns. Traveling waves in different areas of the brain are characterized by wave speeds that span at least two of orders of magnitude, from slow retinal waves with speeds on the order of 0.1 mm/s [39] to fast cortical waves with speeds reaching 17 mm/s [9]. On the other hand, time scales for STDP are typically 10–100 ms [40], but time scales on the order of seconds are predicted to be relevant to retinal waves [41]. We consider in the Discussion how the theoretical results above might apply to these different biological circuits. To do this, we first obtain a more complete picture of the spatial scales of pattern formation predicted by the theory over a wide range of wave speeds and STDP time scales.

**Fig 3:**
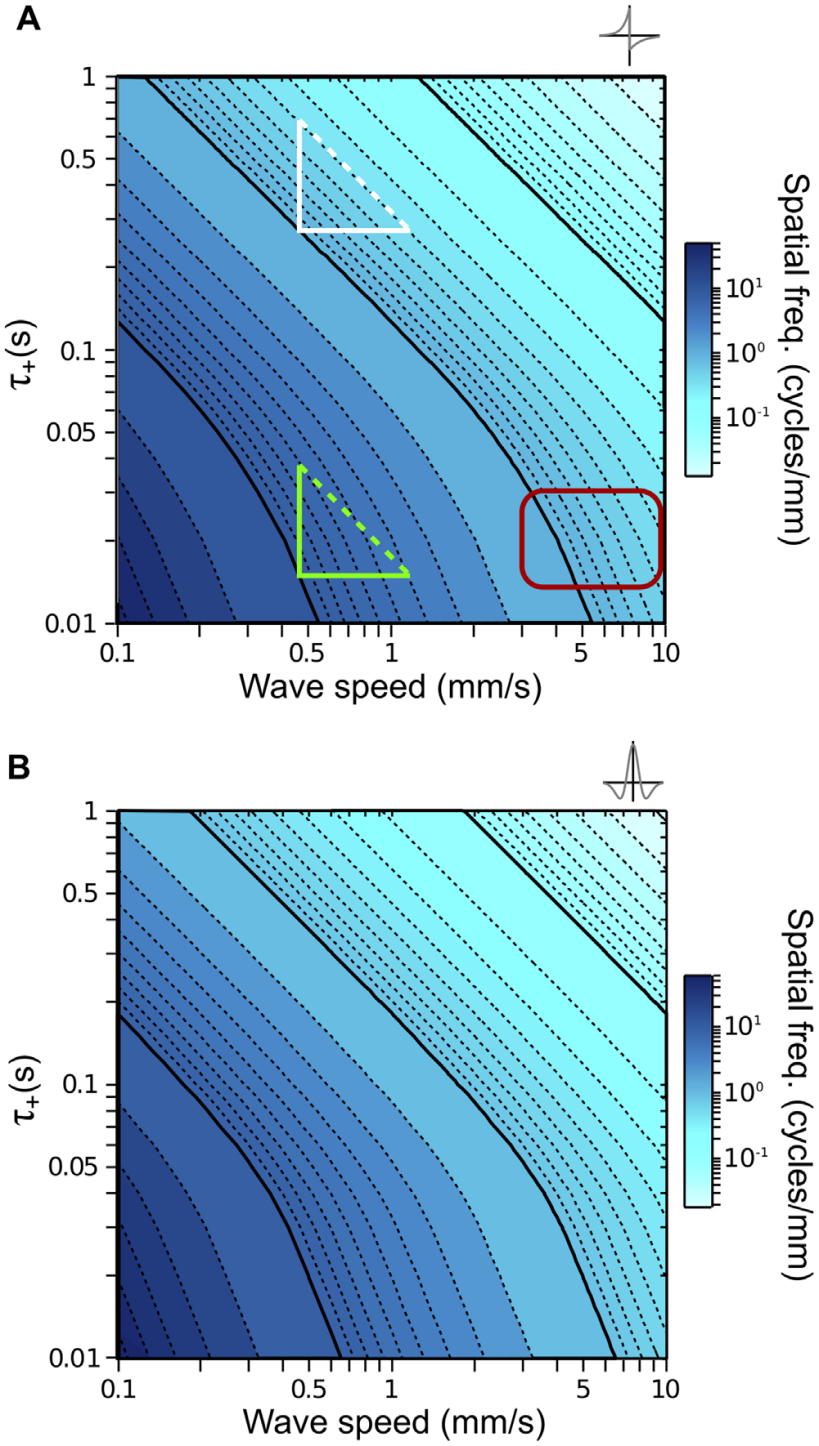
Predicted spatial frequencies as a function of the wave speed and STDP time scale. In all plots, solid black contours denote spatial frequencies equal to 10 raised to integer exponents. **A.** Predicted spatial frequencies for an asymmetric STDP rule as a function of *v* and *τ*_+_. White triangle: when *τ*_+_ is longer than the burst duration, scaling *τ*_+_ or *v* by the same amount has the same effect on the spatial frequency. Green triangle: when *τ*_+_ is shorter than the burst duration, the burst duration has a noticeable influence on the spatial frequency. Red rectangle: wave speeds recorded in the cerebellum, ventral cortex and hippocampus of neonatal mice occupy this region for STDP rules with *τ*_+_ ≈ 20 ms. **B.** Landscape of predicted spatial frequencies for a symmetric STDP rule.

We calculated the landscape of spatial frequencies as a function of *τ*_+_ and *v* by numerically computing $\tilde{\kappa }(k)$, and finding the spatial frequency, k*, that maximizes its real component. The k* landscapes for both the asymmetric and symmetric STDP rules (Fig 3A and 3B, respectively) reveal a remarkably similar dependence of the predicted spatial frequency on *τ*_+_ and *v*, which is perhaps not surprising given the simple exponential functions underlying the STDP rules (Eqs 21 and 22). In particular, for a large region of parameter space, scaling either *v* or *τ*_+_ by a factor, *f*, simply scales k* by 1/*f*. We illustrate this scaling feature using triangles with two equal sides aligned to the axes, as multiplication in linear space is equivalent to addition in logarithmic space. Starting at one iso-frequency contour (1 cycle/mm, bottom left vertex of white triangle in Fig 3A), a constant step along the *v*-axis moves k* to the same iso-frequency contour (0.04 cycles/mm) as does an equal step along the *τ*_+_-axis. Because the input burst acts as a low pass filter on the STDP rule, this relationship does not continue (green triangle, Fig 3A) when *τ*_+_ becomes shorter than the burst duration (here 0.1 s) of the input neurons. Thus, the burst duration has a strong influence on k* at time scales longer than *τ*_+_, which is particularly the case during retinal waves when burst durations are often as long as 3 s [42, 43]. Addressing the impact of the burst duration is the focus of the next section.

### Impact of burst duration on pattern formation

The warping of the spatial frequency landscapes above shows that the burst duration also plays a role in pattern formation. Furthermore, when correlated activity patterns comprise long burst durations, STDP rules with short time scales struggle to extract information from the correlations that might be relevant for circuit development [20, 41, 44]. Here, we further examine the influence of burst duration on pattern formation in our model by carrying out simulations over the range of burst durations that are observed for different types of traveling wave.

**Fig 4:**
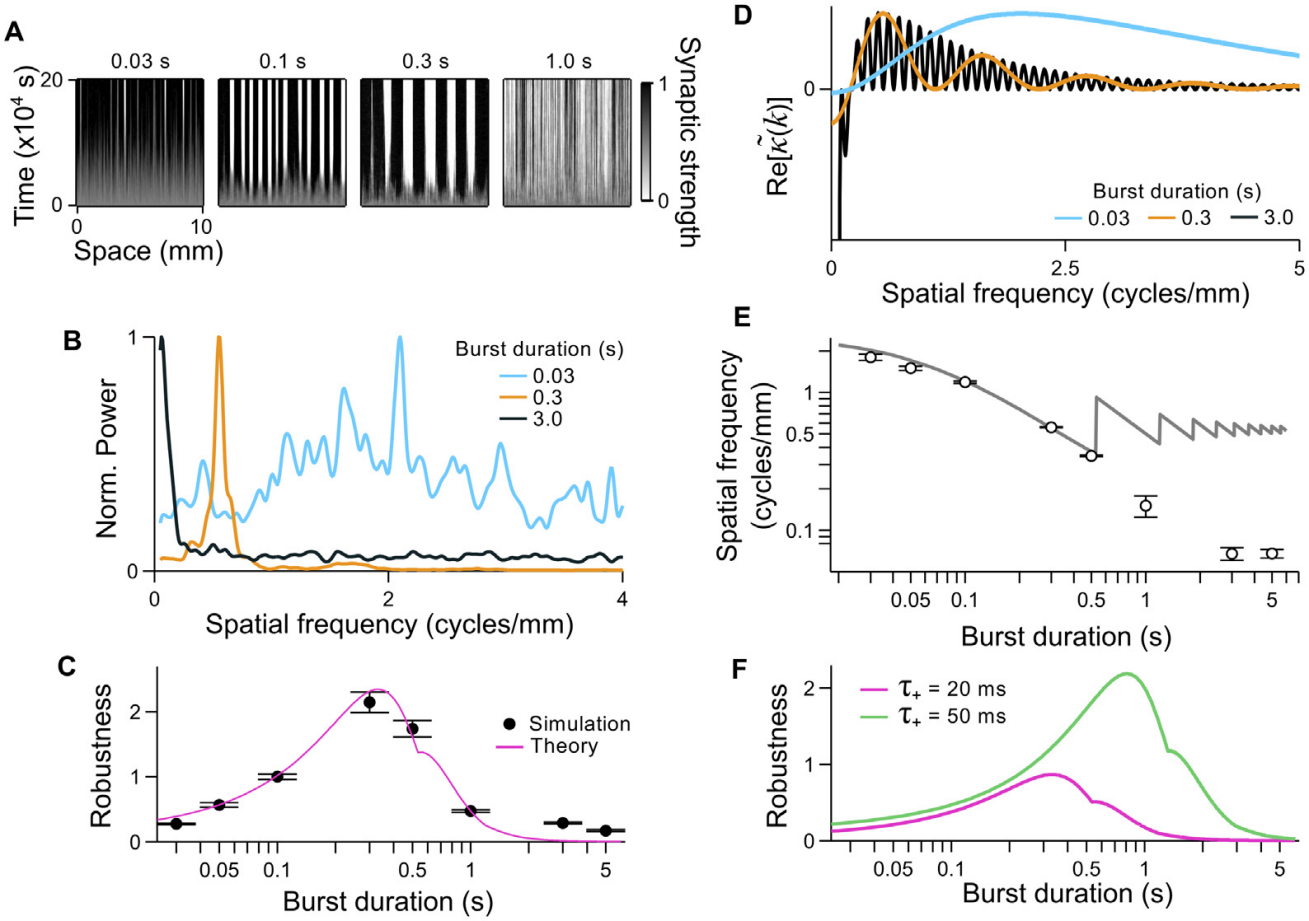
The influence of burst duration on pattern formation. **A.** Examples of synaptic strength evolution over time. Numbers along the top indicate the input burst duration in seconds. **B.** Mean power spectra, normalized by the peak power in each spectrum, of the final connectivity patterns in simulations using burst durations of 0.03 s (blue), 0.3 s (orange), and 3.0 s (black). **C.** Robustness of the periodic connectivity pattern to different burst durations. Reference power spectra are taken from simulations with a burst duration of 0.1 s. Black: robustness measures for each burst duration. Pink: theoretical robustness measure, based on the concentration of power at k* in $\text{Re}[\tilde{\kappa }(k)]$, normalized to the theoretical robustness for a burst duration of 0.1 s. **D.** Examples of $\text{Re}[\tilde{\kappa }(k)]$ for burst durations of 0.03 s (blue), 0.3 s (orange) and 3.0 s (black). The negative lobe in the black curve extends beyond the horizontal axis and has been cut for clarity. **E.** Spatial frequencies of connectivity patterns that developed in simulations as a function of the burst duration (black circles and error bars: mean ± SEM). Grey line: predicted spatial frequencies. The sawtooth fluctuations at longer burst durations correspond to the emergence of new dominant peaks in $\tilde{\kappa }(k)$, which is compressed towards lower spatial frequencies as the burst duration is increased. **F.** Theoretical robustness as a function of burst duration for asymmetric STDP rules with *τ*_+_ = 20 ms (pink) and *τ*_+_ = 50 ms (green).

Waves in this set of simulations traveled with a fixed speed of 3 mm/s, and we used the asymmetric STDP rule with a fixed decay time of *t*_+_ = 20 ms. In Fig 4A, we show examples of the evolving connectivity pattern for different burst durations. For bursts lasting 0.03 s, the connectivity pattern had only a very weak periodic structure (Fig 4A, left panel). The power spectrum of connectivity patterns when the burst duration was 0.03 s, averaged over repeated trials, is shown in Fig 4B (blue). By normalizing the spectrum to the power at its peak, it is clear to see that power is spread over a broad range of spatial frequencies. Burst durations on the order of a few hundred milliseconds produced more distinct periodic connectivity patterns (Fig 4A, middle panels), with power concentrated around the peak in the power spectrum (Fig 4B, orange). However, in simulations with burst durations of 1 s or longer, the connectivity pattern became disordered (Fig 4A, right panel), with some power concentrated at the lowest spatial frequencies and otherwise spread evenly across higher frequencies (Fig 4B, black).

To summarize the robustness with which a periodic connectivity pattern is produced under different conditions, we define *robustness* to be the ratio of the power spectrum amplitude at its peak to the total power in the discretized power spectrum (see Methods). All robustness measures are plotted in comparison to a reference value, which we take to be the mean robustness when parameters yielded a clear periodic connectivity pattern. Here, simulations with a 0.1 s burst duration were used as the reference. The robustness is plotted in Fig 4C for simulations using burst durations from 0.03 s to 5 s (black circles). In agreement with the shapes of the power spectra in Fig 4B, the robustness is relatively high for burst durations in the range 0.1–0.5 s, and relatively low outside of this range. Because non-linearities in the simulation, such as bounded synaptic strengths, allow for the building of multiple spatial frequencies into the connectivity pattern, we examined whether the concentration of power in $\text{Re}[\tilde{\kappa }(k)]$ at the dominant spatial frequency, k*, could explain the relationship between the burst duration and robustness. That is, we numerically computed $\text{Re}[\tilde{\kappa }(k)]$ over the full range of simulated burst durations, from which we calculated the ratio of power at k* to the total power (see Methods). We plot this theoretical measure of robustness in Fig 4C (pink), having normalized it to the theoretical robustness when the burst duration is 0.1 s. The theoretical robustness provides a good estimate for the robustness with which the periodic connectivity patterns are produced in the simulations. To demonstrate how different burst durations distribute power across different spatial frequency ranges, we plot examples of $\text{Re}[\tilde{\kappa }(k)]$ in Fig 4D for three burst durations: 0.03 s (blue), 0.3 s (orange) and 3.0 s (black). For a 0.03 s burst, power is distributed across a wide range of high frequencies, whereas, for a 3.0 s burst, most of the power is concentrated in the negative dip at the lowest frequencies. However, for 0.3 s bursts, power is concentrated between these two extremes, around the dominant spatial frequency of k*. This trend is not specific to the choice of *α*(*t*). In S1A Fig, we show $\text{Re}[\tilde{\kappa }(k)]$ for which *α*(*t*) is modeled using an *alpha* function.

Despite the poor robustness at short burst durations, the spatial frequencies that were measured from the connectivity patterns were well matched to the predicted k* for burst durations between 0.03–0.5 s (Fig 4E). However, for burst durations exceeding 0.5 s, the connectivity patterns no longer yielded spatial frequencies that matched the theory. This is likely to be due to the similar amplitude of multiple peaks in $\tilde{\kappa }(k)$ for longer burst durations (for example, the black curve in Fig 4D), compounded by the overall loss in robustness (Fig 4C). Butts & Rokhsar [41] have shown that waves with long burst durations provide more information that is relevant for refinement when the plasticity rule has a time scale much longer than is typically seen for STDP. It might therefore be possible to rescue pattern formation for waves with long burst durations by using a longer time scale for the STDP rule. To examine this possibility, we computed the fraction of power at $\tilde{\kappa }(k^{*})$ over the range of simulated burst durations for a STDP rule with *τ*_+_ = 50 ms (Fig 4F, green). The wider STDP rule exhibits a greater concentration of power at k* over a much wider range of burst durations, when compared with the STDP rule with *τ*_+_ = 20 ms, and the same trend is seen for other *α*(*t*) kernels (S1B Fig). This includes bursts exceeding 1 s in duration. For burst durations as long as 3 s, which are common for retinal waves, STDP rules with even longer time scales would be expected, according to our model. Thus, our model supports the hypothesis of Butts & Rokhsar [41], and provides a new approach for estimating the required time scale of STDP with which retinal waves may refine developing neural circuits.

Additional contributions to wave-related correlations can arise from the noisy mechanisms of wave and spike generation during early development, which may deliver waves in quick succession as well as generate non-wave related input spikes. The extent to which our analytical results can be applied to biological systems therefore depends on their robustness to these additional contributions to *C*(*x*,Δ*t*). In the following sections, we seek to understand how the presence of noise and multiple waves may impact upon the development of periodic connectivity patterns.

### Sensitivity of the connectivity pattern to noise

**Fig 5:**
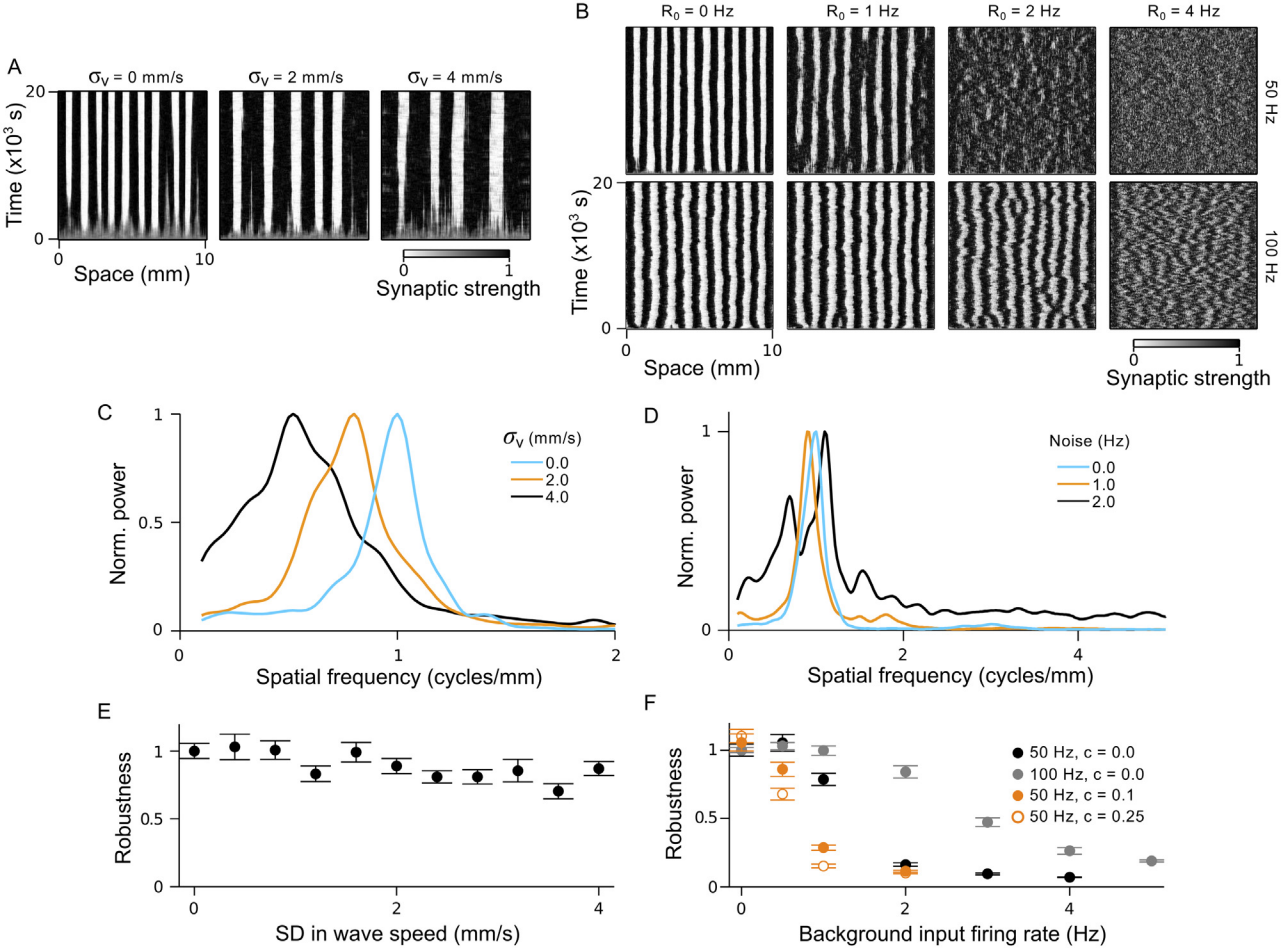
Robustness of periodic connectivity pattern to variation in wave speed and background input spikes. **A & B.** Examples of synaptic strength evolution over time for simulations in which: **A)** wave speeds were drawn from a lognormal distribution with mean 4 mm/s and standard deviation *σ*_*v*_; **B)** a background firing rate of *R*_0_ was assigned to input neurons. The firing rate during a wave burst was 50 Hz (top row) or 100 Hz (bottom row). **C & D.** Mean power spectra for the connectivity patterns, normalized by the maximum in each spectrum, for simulations in which: **C)** wave speeds were drawn from a lognormal distribution with *σ*_*v*_ = 0 mm/s (blue), *σ*_*v*_ = 2 mm/s (orange) and *σ*_*v*_ = 4 mm/s (black); **D)** input neurons were assigned a background firing rate of 0.0 Hz (blue), 1.0 Hz (orange) and 2.0 Hz (black), with 50 Hz firing rates during a wave burst. **E & F.** Robustness of periodic connectivity patterns to: **E)** variation in wave speed, where reference power spectra are from simulations with *σ*_*v*_ = 0; **F)** background spiking of input neurons, where reference power spectra are from simulations with *R*_0_ = 0. Black: 50 Hz firing rate during a wave. Grey: 100 Hz firing rate during a wave. Orange: 50 Hz firing rate during a wave, with local input correlations of strength *c* = 0.1 (closed circles) and *c* = 0.25 (open circles).

Thus far, we have tested theoretical predictions using idealized wavefronts. In reality, spontaneous and sensory driven waves exist among continuous background activity and travel with variable wave speeds. Here, we use simulations to establish the sensitivity of pattern formation to these sources of noise. To examine the effect of variable wave speed, we conducted a set of simulations in which a new speed was assigned to each wave from a lognormal distribution, with a mean of 4 mm/s and standard deviation (SD) *σ*_*v*_. We imposed a minimum speed of 0.05 mm/s so that waves would not take too long to traverse the input layer. Examples of the evolving connectivity pattern in these simulations are provided in Fig 5A and, in Fig 5C, we plot the mean power spectra of the final connectivity patterns for *σ*_*v*_ = 0.0, 2.0 and 4.0 mm/s. An unexpected feature of the resulting connectivity patterns was a decrease in the spatial frequency with increasing *σ*_*v*_ (orange: *σ*_*v*_ = 0.0 Hz, cyan: *σ*_*v*_ = 2.0 Hz, black: *σ*_*v*_ = 4.0 Hz). In these simulations, the developing connectivity pattern may experience a greater influence from the faster waves that recruit more input neurons per unit time and thus drive higher output firing rates. This would correspond to our earlier results in Fig 2, for which we showed that lower spatial frequencies are associated with higher wave speeds. In Fig 5E, we plot the robustness of the connectivity pattern to variation in wave speed, where we have referenced robustness to the case when *σ*_*v*_ = 0. Despite the variation in wave speed, which corresponds to there being a spectrum of spatial frequencies impressed on the network, the connectivity retains a reasonably robust periodic structure.

We next tested the robustness of periodic connectivity patterning to the presence of background spiking noise, which diminishes the relative contribution of wave-activity to *C*(*x*,Δ*t*) in Eq 3. To implement background noise in the simulation, input neurons that were not participating in a wave fired spontaneous spikes with a firing rate of *R*_0_, which was varied between simulations (see Methods). Examples of the evolving connectivity patterns are shown in Fig 5B (top row) for different background firing rates. Mean power spectra of the final connectivity patterns when *R*_0_ = 0.0 (cyan), 1.0 (orange) and 2.0 Hz (black) are plotted in Fig 5D, and show little variation in the dominant spatial frequency when *R*_0_ < 2.0 Hz. However, the robustness degraded substantially when *R*_0_ ≥ 2.0 Hz (black circles in Fig 5F, reference case *R*_0_ = 0 Hz). In these simulations, a new wave traversed the input layer every 10 s, but the burst duration was only 0.1 s. Therefore, the ratio of wave-related spikes to background spikes, rather than the background rate, might better parameterize when the connectivity pattern will be robust. In this case, periodic patterns lacked robustness when the ratio went below approximately 1:4. Accordingly, the robustness could be significantly enhanced for almost all background rates by increasing the firing rate during a wave burst to 100 Hz. In this case, the robustness degraded substantially when *R*_0_ ≥ 4 Hz (Fig 5B, bottom row; Fig 5F, grey circles). During early, spontaneous waves, low background firing rates are typical, and there is good reason to believe that background firing rates are low in early stages of sensory processing as well [45]. We review more evidence for this in the Discussion.

Spike-spike correlations induced by the wavefront, rather than time averaged firing rates, are the driving force behind periodic patterning in our model. Additional sources of spike-spike correlations may therefore disrupt periodic patterning. We conducted a set of simulations in which inputs experienced instantaneous correlations with their neighbors (see Methods) while also varying the background firing rate. With non-zero background rates, increasing the correlation strength does indeed degrade the robustness of the periodic connectivity patterns (Fig 5F, orange circles), referenced to simulations with no local correlations and *R*_0_ = 0.0 Hz. In the absence of background spiking, however, the additional local correlations act to enhance the robustness. Typically, correlations are not instantaneous but decay over time [46–49] and thus would have a reduced impact on plasticity due to the decaying amplitude of the STDP rules around Δ*t* = 0 [18].

### Sensitivity of the connectivity pattern to multiple, non-isolated waves

The intervals between consecutive spontaneous waves can be very variable, and inter-wave intervals (IWIs) cover a broad range, from approximately 100 ms in the cerebellum [8] to tens of seconds in the retina [39, 50]. IWIs during sensory driven waves may also be highly variable, and are likely to match the temporal pattern of naturally occurring stimulus features to which a population of input neurons are tuned. During vision, for example, multiple luminance contours can be cast across the retina in quick succession when tracking a moving object. In the following, we investigate how the presence of multiple waves, which simultaneously drive spiking in the output neuron, impact the development of the connectivity pattern. In so doing, we test our assumption in the above derivation that waves be sufficiently isolated in time.

We ran a set of simulations in which waves were generated with an approximately lognormal distribution of IWIs and varied the mean IWI, *μ*_IWI_, between simulations (see Methods). At a speed of 4 mm/s, waves took 2.5 s to traverse the input layer. Thus, to ensure that multiple waves were present for the majority of the simulation, we set *μ*_IWI_ ≤ 2.0 s across the set of simulations.

**Fig 6:**
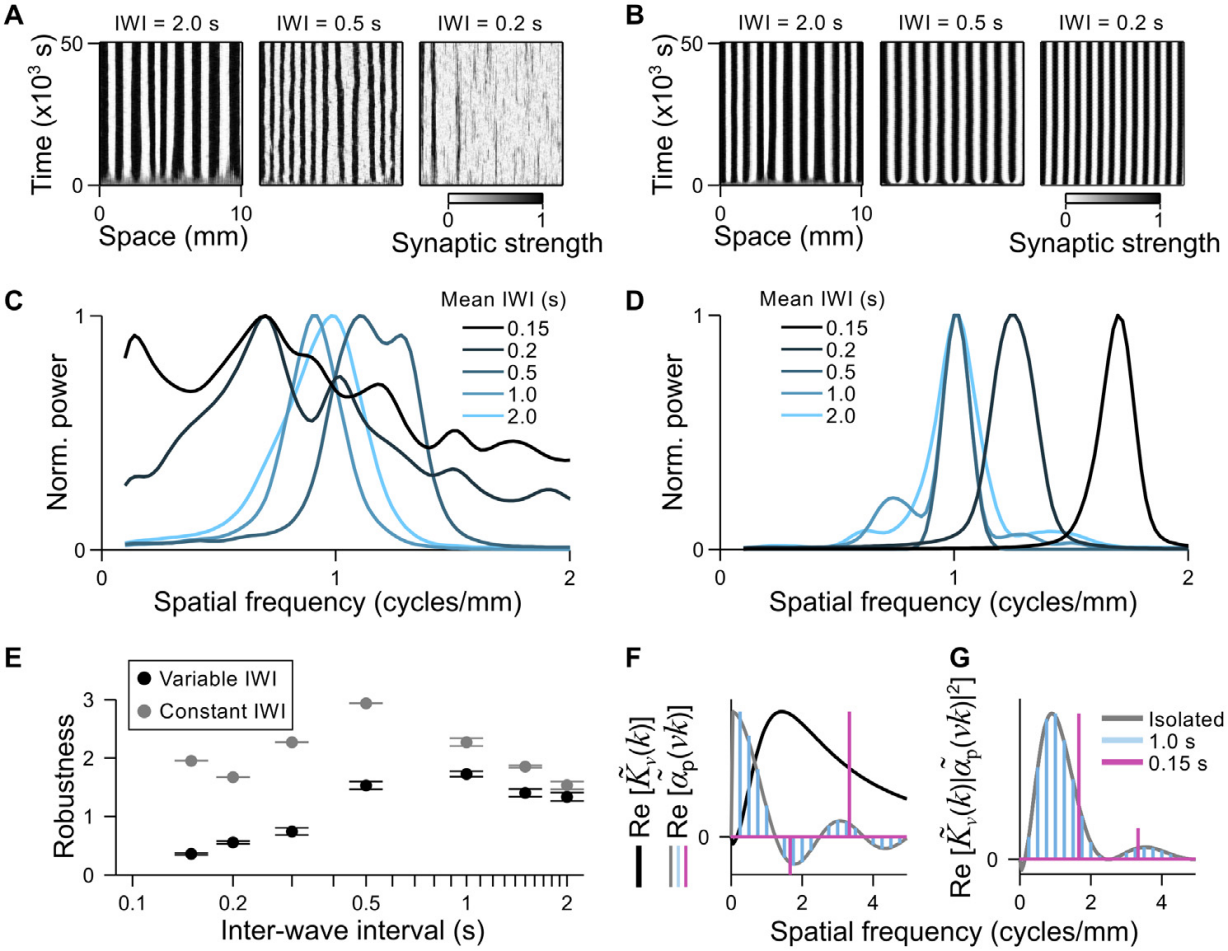
Robustness of periodic connectivity patterns when multiple waves traverse the inputs simultaneously. **A & B.** Examples of synaptic strength evolution over time for simulations in which: **A)** waves are generated with IWIs drawn from an approximately lognormal distribution; **B)** waves are generated regularly with a constant IWI. **C & D.** Power spectra for the connectivity patterns, normalized by the maximum in each spectrum, for simulations in which: **C)** IWIs are drawn from a lognormal distribution. Light blue to black corresponds to mean IWIs of 2.0 s, 1.0 s, 0.5 s, 0.2 s and 0.15 s, respectively; **D)** IWIs are constant. Color scheme the same as for **C**. **E**. Robustness of connectivity patterns as a function of the mean IWI. Reference power spectra are taken from simulations with a constant IWI of 5 s. Black: IWIs drawn from a lognormal distribution. Grey: IWIs are constant. **F.** An example of $\text{Re}[{\tilde{K}}_{v}(k)]$ (black) and examples of $\text{Re}[{\tilde{\alpha }}_{\text{p}}(vk)]$ for a single wave in isolation (grey) and multiple waves with constant IWIs of 1 s (blue) and 0.15 s (pink). **G.** Examples of $\text{Re}[{\tilde{K}}_{v}(k)|{\tilde{\alpha }}_{\text{p}}(vk){|}^{2}]$ for the same IWIs in **F**.

Despite multiple waves driving the output cell, periodic connectivity patterns emerged in the simulations, examples of which are shown in Fig 6A (left and center panels). Power spectra of the connectivity patterns had distinct peaks near the predicted spatial frequency (0.91 cycles/mm) for *μ*_IWI_ as short as 0.5 s (Fig 6C). The connectivity pattern began to degrade for *μ*_IWI_ < 0.5 s, and had little structure when *μ*_IWI_ ≤ 0.2 s (Fig 6A, right panel), with power spread broadly across the spectrum (Fig 6C). The loss of robustness with decreasing *μ*_IWI_ is summarized in Fig 6E (black circles), where robustness is referenced to simulations in which a constant IWI of 5 s was used, thus ensuring that only one wave was present at a time.

The loss of robustness with decreasing *μ*_IWI_ may be related to our requirement in the above derivation that waves be sufficiently isolated in time, with Δ*T* > 2𝓚, where Δ*T* can be interpreted as the IWI and 𝓚 as half the temporal width of the STPD rule. Although 𝓚 is not precisely defined, values in the range 0.1–0.3 s would be reasonable for the STDP rule used here, for which the decay time of the longer, negative lobe was *τ*_−_ = 0.04 s. An alternative explanation might be that successive waves with very short IWIs are not very different from single waves with long burst durations, as far as the timing precision of the STDP rule is concerned. Because the burst duration also influences robustness, these two possible factors would be difficult to disentangle, and efforts to do so go beyond the scope of this study. The important qualitative result is that, above a lower limit to the mean IWI of only a few tenths of a second, the succession of randomly occurring waves does not greatly degrade the robustness of periodic connectivity patterns.

We next asked whether a constant IWI between waves, which would contribute a strong frequency component to $\tilde{\kappa }(k)$, would lead to a similar degradation in robustness with decreasing IWI. Because wavefronts of activity in the retina readily track moving luminance edges [7], regular waves could correspond to stimulation of the retina by luminance gratings, as used in experiments studying the development of orientation and direction selectivity [51]. We ran simulations in which waves traversed the inputs in a periodic fashion, with a constant IWI, but otherwise used the same parameters as for the simulations with irregular waves. Examples of the periodic patterns that developed for different IWIs are provided in Fig 6B, and their power spectra are shown in Fig 6D. It is clear that regular waves built robust periodic patterns, even with the shortest IWIs. Our robustness measure confirmed that this was the case across all of the IWIs tested (Fig 6E, grey circles). In addition to the enhanced robustness, a distinct feature of the connectivity patterns with constant IWIs is the shift towards higher spatial frequencies for short IWIs (Fig 6D, power spectra for IWIs of 0.2 s and 0.15 s).

The emergence of robust periodic connectivity patterns, with increased spatial frequencies for fixed IWIs ≤ 0.2 s, appears at odds with our theoretical predictions. However, these features can be easily accounted for by considering how *κ*(*x*) is constructed in Eq 7. To represent the periodic input bursts elicited by multiple waves, we replace *α*(*t*) in Eq 7 with $\alpha_{\text{p}}(t)=\int_{-\infty }^{\infty }dt^{\prime }\sum_{n}\delta (t^{\prime }-(n\times \text{IWI}))\alpha (t-t^{\prime })$, the convolution of *α*(*t*) with a *Dirac comb*. Eq 7 expresses *κ*(*x*) as the convolution of *K*_*v*_(*x*), *α*_p_(*x*/*v*), *α*_p_(−*x*/*v*) and *ϵ*(*x*/*v*), and can be solved by taking its Fourier transform, whereby convolution in the real domain is equivalent to multiplication in the frequency domain. In Fig 6F, we plot the two functions that have the greatest influence in our simulations. In black is the Fourier transform of the STDP rule, ${\tilde{K}}_{v}(k)$. The remaining curves are the Fourier transforms of the input firing patterns, ${\tilde{\alpha }}_{\text{p}}(vk)$ (with the zeroth frequency component removed), for three cases: a single wave in isolation (effectively, IWI = ∞ grey curve) and periodic waves with IWI = 1.0 s (blue) and IWI = 0.15 s (pink). Note that the Fourier transform of the EPSP has little effect in this calculation because its time constant is much shorter than that of the other functions. The product ${\tilde{K}}_{v}(k)|{\tilde{\alpha }}_{\text{p}}(vk){|}^{2}$ therefore yields a good approximation of $\tilde{\kappa }(k)$, and is drawn for the three cases of ${\tilde{\alpha }}_{\text{p}}(vk)$ in Fig 6G. Because the Fourier transform of a *Dirac comb* is another *Dirac comb*, ${\tilde{\alpha }}_{\text{p}}(vk)$ produces sharp peaks in $\tilde{\kappa }(k)$ (pink and blue) at spatial frequencies that may be higher (pink) than the peak in the case of isolated waves (grey). The large amplitude and narrow peaks in $\tilde{\kappa }(k)$, when IWI = 0.15 s and 0.2 s, explain the dominance and robustness of a single spatial frequency in the respective connectivity patterns, and the positions of these peaks explain the shift of the power spectra toward higher spatial frequencies in Fig 6D.

The strong dependence of the spatial frequency on the IWI for short IWIs only, as depicted in Fig 6F and 6G, suggests a critical value for the IWI. We can relate the critical value to the time taken for a wave to travel a distance equal to one cycle of the periodic pattern: IWI_crit_ = 1/vk* ≈ 0.27 s. That is to say, when IWI < IWI_crit_, the spatial frequency with which waves are lined up along the input layer exceeds the dominant spatial frequency of *κ*(*x*) for a wave in isolation (S2 Fig). This interpretation fits well with our assumption in the derivation that waves be sufficiently isolated in time (and therefore isolated in space). Moreover, the existence of a critical IWI provides a means for quantifying 𝓚, and thus provides a possible explanation for the degradation in robustness when *μ*_IWI_ ≤ 0.3 s in the simulations with irregular waves.

### Traveling waves are instructive for shaping receptive fields via spike-timing dependent plasticity

**Fig 7:**
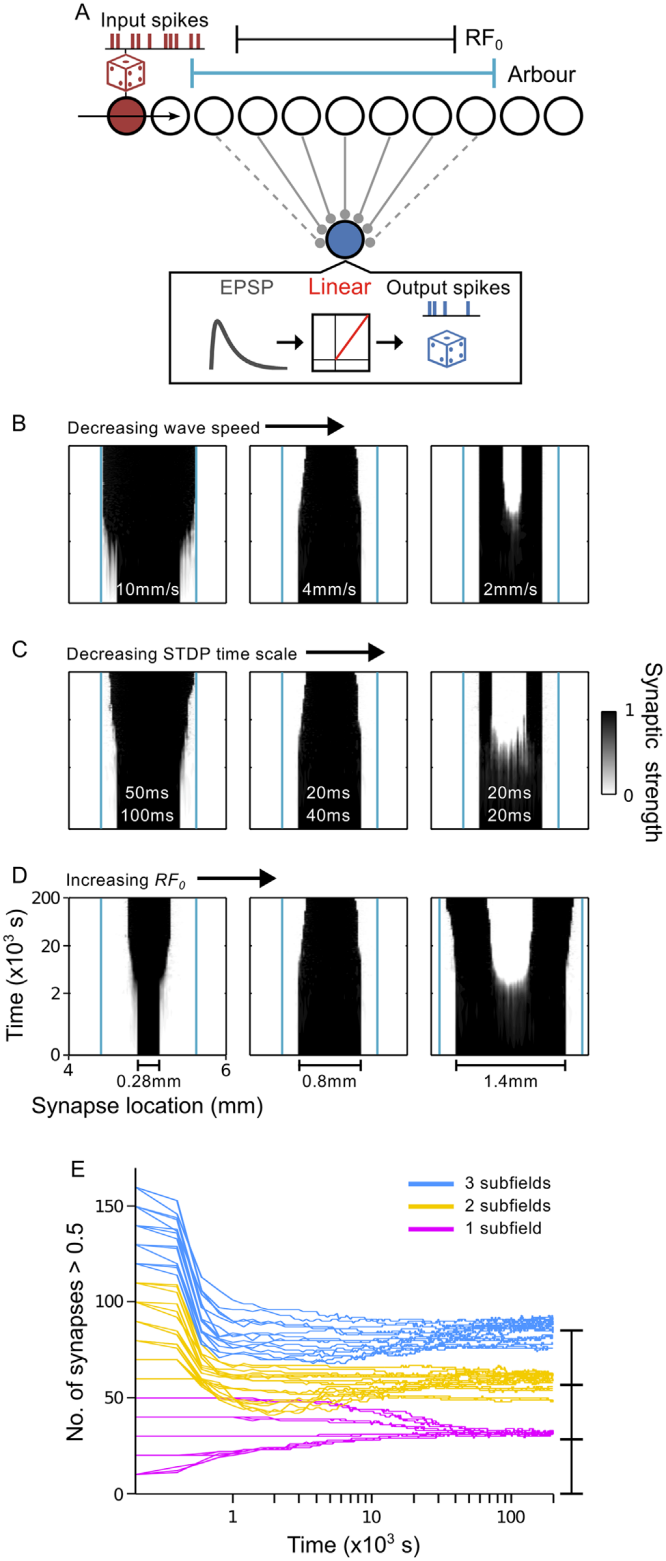
Refinement of receptive fields. **A**. Schematic of network architecture for simulations investigating RF development. Grey solid connectors: synapses at maximal strength comprise the initial RF. Grey dashed connectors: synapses with zero strength at the start of the simulation but are modifiable, as they lie within the arbor function (teal). **B–D**. Examples of RF development over time for different *RF*_0_, *v* and *τ*_+_ combinations. Teal lines mark the boundary of the arbor function. **B**. Left → centre → right: *v* = 10 mm/s, 4 mm/s and 2 mm/s, keeping *τ*_+_ = 20 ms and *RF*_0_ = 0.8 mm fixed. **C**. Left → centre → right: *τ*_+_ = 50 ms, 20 ms and 20 ms, *τ*_−_ = 100 ms, 40 ms and 20 ms, *A*_−_ = 0.55, 0.55 and 1.1, keeping *v* = 4 mm/s and *RF*_0_ = 0.8 mm fixed. **D**. Left → centre → right: *RF*_0_ = 0.28 mm, 0.8 mm and 1.4 mm, keeping *v* = 4 mm/s and *τ*_+_ = 20 ms fixed. **E**. Development of the mean number of synapses with strength > 0.5 in simulations using *RF*_0_ values from 0.2 mm to 3.2 mm in steps of 0.2 mm. Traces tend towards one of three values, depending on the number of subfields that develop. Blue: RFs split into three subfields; yellow: RFs split into two subfields; purple: RFs maintain just a single field.

The filtering properties of a RF result from feedforward and recurrent synaptic inputs to the neuron, as well as its intrinsic cell properties. In this section, we used simulations of spiking neurons to examine whether the interaction of traveling waves and STDP rules, which can impose spatially periodic connectivity patterns on a uniform field of synaptic strengths (shown above), might be a plausible mechanism for shaping the feedforward component of RFs. As such, a RF in our model refers to the spatial pattern of synaptic strengths impinging on the output neuron from a small region of the input layer. During development, coarse RFs are thought to be set up by molecular cues before activity dependent refinement takes place [52]. Therefore, at the beginning of each simulation, we set synapses from the center of the input layer to maximum strength and the remaining synapses to zero, thus endowing the output neuron with an initial RF having a diameter, *RF*_0_. Despite this restricted initial arrangement of synaptic strengths, we found that a periodic pattern would nevertheless emerge across the entire input array (S3 Fig). This outcome is not typical of the brain, as topographic maps and the limited size of axonal arbors and dendritic trees restrict the spatial distribution of synaptic inputs to a neuron. We therefore applied an arbor function (see Methods) that was centered in the middle of the input layer and spanned a region greater than *RF*_0_. Synapses that were outside the arbor were disconnected from the output neuron and prevented from strengthening. A schematic of this new network architecture is provided in Fig 7A. In the majority of the following simulations, we set *RF*_0_ = 0.8 mm, which approximates an area of retina that corresponds to RF sizes in developing areas of the visual system, including the cat primary visual cortex [53, 54], the lateral geniculate nucleus (LGN) in ferrets [55, 56], and the superior colliculus (SC) in adult mice that have had disrupted retinal waves. The conversion between RF size and retinal distance is described in the Methods. Furthermore, in the remaining sections, we increase the bias for synaptic weakening in the asymmetric STDP rule from *A*_−_ = 0.51 to *A*_−_ = 0.55, which is useful for RF refinement. Later, we examine the influence of this bias in greater detail.

By interpreting the output neuron RF as a single cycle in the type of periodic connectivity patterns obtained above, we are able to examine how properties such as the wave speed and the STDP time scale may shape RF development by controlling the characteristic wavelength, 1/k*, of the connectivity pattern. Depending on the size of 1/k* with respect to *RF*_0_, one of three modes of RF modification were observed. In one set of simulations, the RF became larger (Fig 7B, left), smaller (Fig 7B, middle) or split into subfields (Fig 7B, right) with progressively slower wave speeds and thus shorter characteristic wavelengths. The same modes of RF modification were achieved by decreasing the STDP time scales (Fig 7C) and holding the wave speed constant. If instead of changing k* we increased *RF*_0_, keeping the wave speed and STDP rule fixed, the RFs were similarly modified (Fig 7D). Thus, characteristic wavelengths that are long relative to *RF*_0_ caused the RF to grow, shorter wavelengths caused the RF to shrink, and even shorter wavelengths enabled multiple cycles of the periodic pattern to form within the arbor.

Our results suggest that 1/k* in fact determines the size of the final RF, or each subfield. To verify this, we varied *RF*_0_ from 0.2 mm to 3.2 mm between simulations, with the wave speed and STDP rule fixed, and looked for the convergence of RF sizes, which we measured as the number of synapses with strengths > 0.5. To accommodate *RF*_0_, the width of the arbor function was set to the larger of 0.8 mm or *RF*_0_+0.4 mm. RF sizes converged to one of three sizes (Fig 7E), depending on whether the RF maintained a single field (purple), or split into two (yellow) or three (blue) subfields. The larger *RF*_0_, the more subfields that emerged. Regardless of *RF*_0_, the mean final size of each subfield was approximately the same. Integer multiples of the mean subfield size (measured from RFs with three subfields) are drawn on the right of Fig 7E to illustrate this fact. Small biases were evident, however, whereby larger values of *RF*_0_ tended to give rise to larger RFs or subfields. This considered, the final number of strong synapses were easily distinguishable between cases in which one, two or three subfields developed. Thus, the size and shape of the final RF tightly corresponds with the wave, STDP and initial RF properties.

### A quantitative relationship between periodic patterning and refinement of receptive fields

**Fig 8:**
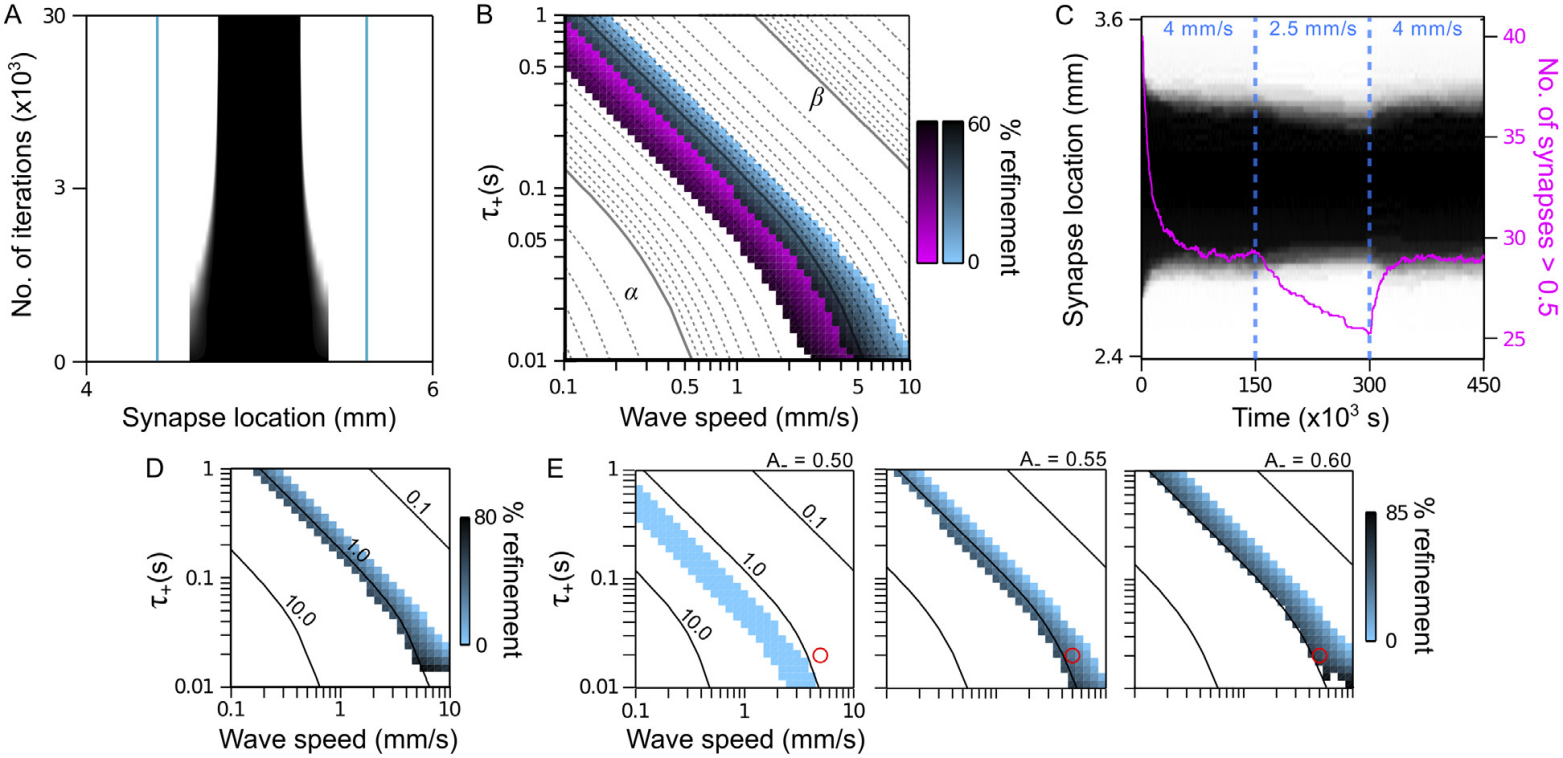
Relationship between periodic patterning and receptive field refinement. **A**. Example of RF refinement obtained by numerically solving Eq 6. Vertical teal lines denote the arbor function boundary. **B**. Refinement phase space for RFs as a function of wave speed and STDP time constants. Colored regions: areas of phase space in which RFs maintain a single field that is refined to smaller sizes; blue: *RF*_0_ = 0.8 mm; purple: *RF*_0_ = 0.44 mm. Shading indicates the total change in RF size as a percentage of *RF*_0_. Region *α* corresponds to higher spatial frequencies in which RFs split into subfields. Region *β* corresponds to lower spatial frequencies in which RFs expand. Overlaid are the iso-frequency contours of Fig 3. Solid contours correspond to spatial frequencies with integer exponents of 10 (lower left to upper right: 10 cyc./mm, 1 cyc./mm and 0.1 cyc./mm). **C**. Incremental changes in wave speed can continually refine RFs while maintaining a single subfield structure. Shown is the development of a RF, averaged over 16 trials, during which the wave speed is first set to 4 mm/s and then decreases to 2.5 mm/s, after which the RF decreases in size. Increasing the wave speed back to 4 mm/s returns the RF to its previous refined size. Purple trace: plot of the mean number of synapses with strength > 0.5 as an indicator of RF size. **D**. Refinement phase space using a symmetric STDP rule, for which *RF*_0_ matches that for the blue region in **B**. Numbers next to the iso-frequency contours indicate their spatial frequency in cycles/mm. **E**. Refinement phase spaces, using the asymmetric STDP rule, for different biases towards synaptic weakening. Left: *A*_−_ = 0.50, which corresponds to $\int_{\infty }^{\infty }d\Delta t\;K(\Delta t)=0$. Centre and right: *A*_−_ = 0.55 and 0.60, respectively, which correspond to $\int_{\infty }^{\infty }d\Delta t\;K(\Delta t)>0$. Red circles lie at the same *v* and *τ*_+_ coordinates, to compare the effect of different *A*_−_ values on RF refinement. The refinement phase space moves to lower spatial frequencies as *A*_−_ increases.

We showed above, in Fig 7B and 7C, that different wave and STDP parameter combinations could yield the same mode of RF modification, including RF expansion, contraction, or splitting. Here, we focus on RFs that are made to contract, which is particularly relevant to the refinement of topographic maps such as the retinotopic map in superior colliculus, and examine the range over which the wave speed and STDP time scale achieves this mode of modification. To do this, we numerically integrated Eq 6 (see Methods) for the wide range of wave speeds and STDP time constants used to generate Fig 3, holding *RF*_0_ = 0.8 mm constant. An example of *w*(*x*) during the numerical integration of Eq 6, starting with an initial RF, is shown in Fig 8A, for which parameters matched those in the central panels of Fig 7B and 7D. Using the solutions for *w*(*x*), we constructed a phase diagram for the size and shape of RFs that developed, which we call the refinement phase space.

In the refinement phase space, shown in Fig 8B, there is a region (shaded blue) corresponding to *v* and *τ*_+_ parameters that caused the RF to contract, with darker shades of blue indicating greater contraction, measured as a percentage of *RF*_0_. In the region labelled *α* (unshaded), at which *κ*(*x*) has higher characteristic spatial frequencies, RFs split into subfields. In the region labelled *β* (unshaded), at which spatial frequencies are lower, RFs expanded. Overlaid are the iso-frequency contours of the predicted spatial frequencies, k*, as in Fig 3. The boundaries between each mode of RF modification closely follow the iso-frequency contours, with greater RF contraction (darker shades) occurring at higher spatial frequencies. Because the outcome of the final RF depends on the initial RF size, we recomputed the refinement phase space, setting *RF*_0_ = 0.44 mm, which just exceeded the smallest size of RFs after contracting in solutions when *RF*_0_ = 0.8 mm. For *RF*_0_ = 0.44 mm, the region of phase space in which RFs contracted (colored purple) corresponded to slower wave speeds and shorter STDP time constants, i.e. higher spatial frequencies in *κ*(*x*). Taken together, these results confirm that smaller initial RF sizes require higher spatial frequencies in *κ*(*x*) to achieve a particular mode of RF modification. The area of the blue and purple regions of phase space also show that, in order for RFs to be refined to a smaller size by the asymmetric STDP rule, the maximum possible wave speed can be just over a factor of two times greater than the minimum possible wave speed. At least the same range in *τ*_+_ can also enable RF contraction. Thus, the mechanism of RF contraction described here allows for some variation in STDP properties between synapses, variation in wave speed, or even fluctuations in both of these phenomena over time.

An interesting corollary of these results is that changes in wave speed and STDP properties can follow changes in RF size, thus allowing continued contraction beyond that of a fixed *v* and *τ*_+_ combination. To demonstrate this point, we ran a simulation using the same parameters as those used for the central panels in Fig 7B and 7D. However, once the RF had refined to a smaller size, we decreased the wave speed from 4 mm/s to 2.5 mm/s. Note that, according to Fig 8B, *v* = 2.5 mm/s and *τ*_+_ = 20 ms would cause a RF with *RF*_0_ = 0.8 mm to split into subfields, as this point in the phase space lies to the lower left of the blue shaded region. However, as shown in Fig 8C, an initial period of contraction with *v* = 4 mm/s set up further contraction, after a decrease in wave speed to *v* = 2.5 mm/s, without splitting the RF into subfields. Increasing the wave speed back to 4 mm/s at a later time caused the RF to expand and return to its previous size. Analogously, changes in the time constants of STDP can achieve the same effect.

The phase diagram corresponds very well with the simulation results in Fig 7, confirming our hypothesis that *κ*(*x*) and *RF*_0_ jointly determine the final shape and size of the RF. This was not unique to the asymmetric STDP rule. In Fig 8D, we show a similar correspondence between refinement and the iso-frequency contours for a symmetric STDP rule.

Tuning the characteristic wavelength by itself was not sufficient to achieve RF contraction. In addition, both asymmetric and symmetric STDP rules required a bias towards synaptic weakening. In Fig 8E, we plot the refinement phase space of a 0.8 mm RF for three asymmetric STDP rules that differed only in *A*_−_, which scaled the amplitude of the negative lobe in the rule. When *A*_−_ = 0.5, we have $\int_{-\infty }^{\infty }\;d\Delta tK(\Delta t)=0$, such that synapses no longer compete for connections with the output. Consequently, increasing the dominant spatial frequency of *κ*(*x*) would not decrease the size of the RF before causing it to split into subfields (left panel). However, by increasing *A*_−_ (*A*_−_ = 0.55, middle panel; *A*_−_ = 0.6, right panel), such that $\int_{-\infty }^{\infty }\;d\Delta tK(\Delta t)<0$, RF contraction was possible. Furthermore, increasing *A*_−_ moved the iso-frequency contours, making RF contraction possible at lower characteristic spatial frequencies and, therefore, larger *v* and *τ*_+_ values. As such, for a given *v* and *τ*_+_ pair (red circles in Fig 8E), greater contraction was achieved with increased bias for synaptic weakening.

Equipped with a basic understanding of the relationship between periodic patterning and RF modification, we examine how more realistic traveling waves and STDP impact the development of RFs in two dimensions in the following sections.

### Refinement of receptive fields in two spatial dimensions

**Fig 9:**
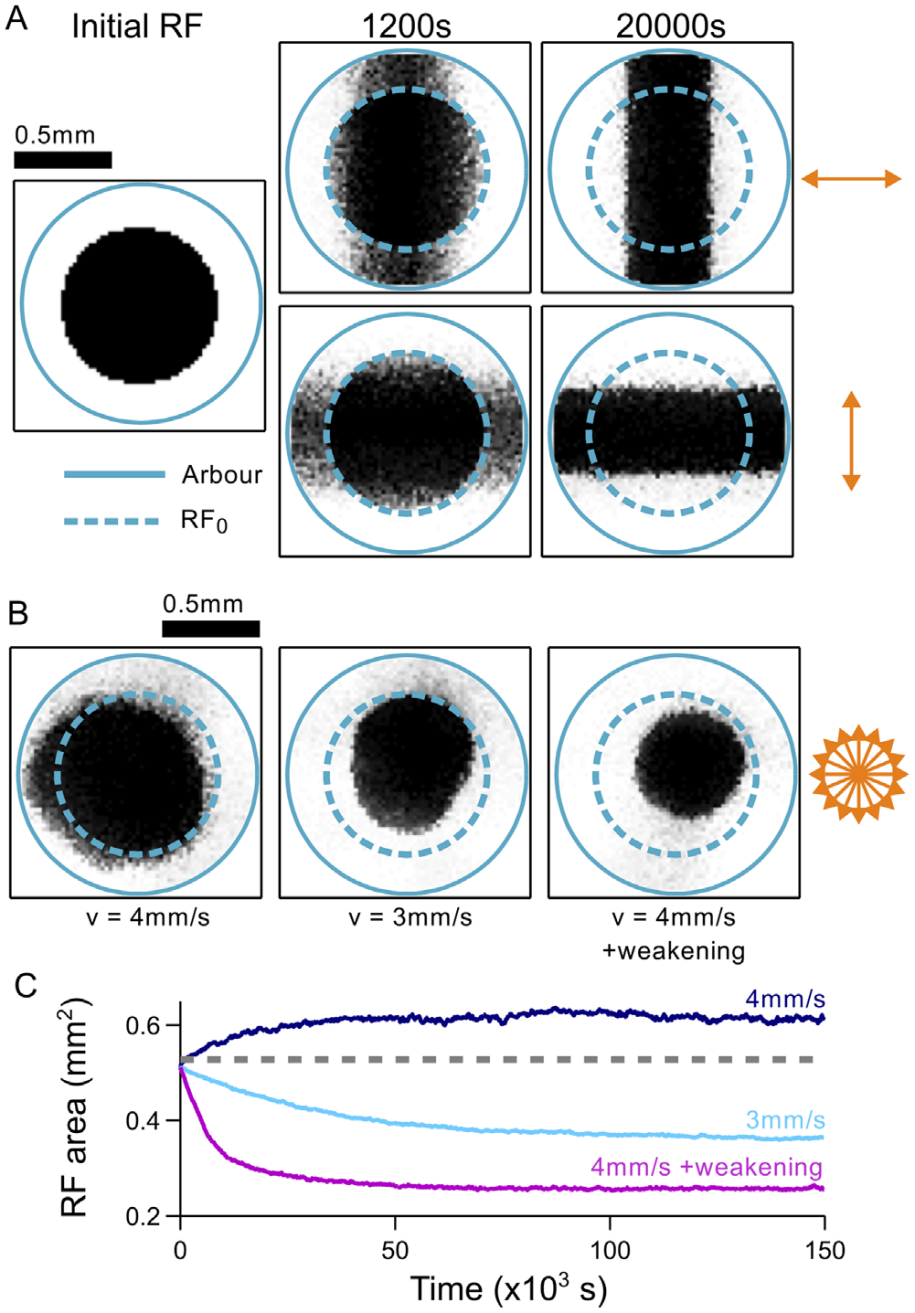
Refinement of receptive fields with 2D plane waves. **A**. Refinement depends on the direction of wave propagation. If an initial RF is circular (left), then waves traveling along a horizontal direction will refine the RF along the horizontal axis only (top row). The RF necessarily expands to the arbor boundary along the orthogonal axis. Likewise, waves traveling along a vertical direction refine RFs along the vertical axis only (bottom row). Increasing time from left to right. Arrows: direction of wave propagation. Solid teal circle: arbor boundary. Dashed green circle: boundary of the initial RF. **B**. Final RFs that were exposed to plane waves traveling randomly in 16 possible directions. Left: using the same wave speed (4 mm/s) and STDP parameters (*τ*_+_ = 20 ms, *A*_−_ = 0.55) as in **A**. Centre: using a slower wave speed (3 mm/s) to lower the characteristic spatial frequency of *κ*(***x***). Right: using the faster wave speed (4 mm/s) but shifting refinement phase space to lower frequencies by increasing the bias for synaptic weakening (*A*_−_ = 0.60). **C**. Summary of the development of RF sizes for each of the conditions in **B**, averaged over three trials for each condition.

In two dimensions, the variables *x* and *v* in Eq 6 can now be considered as 2D vectors, ***x*** = (*x*,*y*) and ***v*** = (*v*_*x*_,*v*_*y*_), in the *x*-*y* plane. As such, we expected the connectivity pattern to adopt the characteristic spatial frequency, k* = (*k*_*x*_,*k*_*y*_), and thus for the type of RF modification along a particular axis to depend on the direction of wave propagation. To test this, we generated wave stimuli in a 2D input layer consisting of 64×64 units arranged on a square lattice. Plane waves were generated in a similar fashion to the 1D scenario, moving with a constant speed in alternating directions, from one side of the input layer to the opposite side with a wave speed of 4 mm/s. The single output neuron was provided with an initial, circular RF 0.8 mm in diameter (Fig 9A, left panel), and synapses were modified by an asymmetric STDP rule (*τ*_+_ = 20 ms). When plane waves travelled along just one axis, the RF contracted along the same axis (Fig 9A; top panels: waves travel along the horizontal axis; bottom panels: waves travel along the vertical axis), corresponding with the 1D results above (Fig 8B). However, the RF grew along the orthogonal axis until it spanned the full extent of the arbor. This corresponds with an effectively infinite wave speed along the orthogonal axis, resulting in a periodic pattern with a spatial frequency of zero.

Exposing the network to a reduced set of wave directions in this way can be used to build RFs that exhibit periodic structure along the same directions. In S4 Fig, we provide examples of RFs that developed when waves traveled in both directions along one, two and three axes. Of particular interest are RFs that exhibit multiple parallel subfields (S4A Fig), which bare several similarities with oriented simple cell RFs in primary visual cortex. In the Discussion, we describe in more detail how periodic patterning might be applied to realistic simple cell RFs.

When waves travelled in 16 possible directions, equally spaced around the compass and in a random sequence, the final RF maintained an approximately circular shape (Fig 9B, left). However, using the same wave and STDP properties as for Fig 9A, the RF area increased (Fig 9C, dark blue) because contraction along the axis parallel to the wave direction was outweighed by expansion along the orthogonal axis. RF expansion could be counteracted by changing parameters that enhance contraction, as explained in the results of Fig 8. Thus, increasing k* by decreasing the wave speed to 3 mm/s (Fig 9B, middle), or increasing the amplitude for synaptic weakening in the STDP rule from *A*_−_ = 0.55 to *A*_−_ = 0.6 (Fig 9B, right), caused RFs to contract (Fig 9C, light blue and purple, respectively). This shows that the principle of using STDP and traveling waves to refine a RF extends from 1D to 2D networks for simple, idealized waves. In the following section, we will examine how this process holds up when waves follow more irregular and complex trajectories.

### Refinement of receptive fields by complex wave patterns

**Fig 10:**
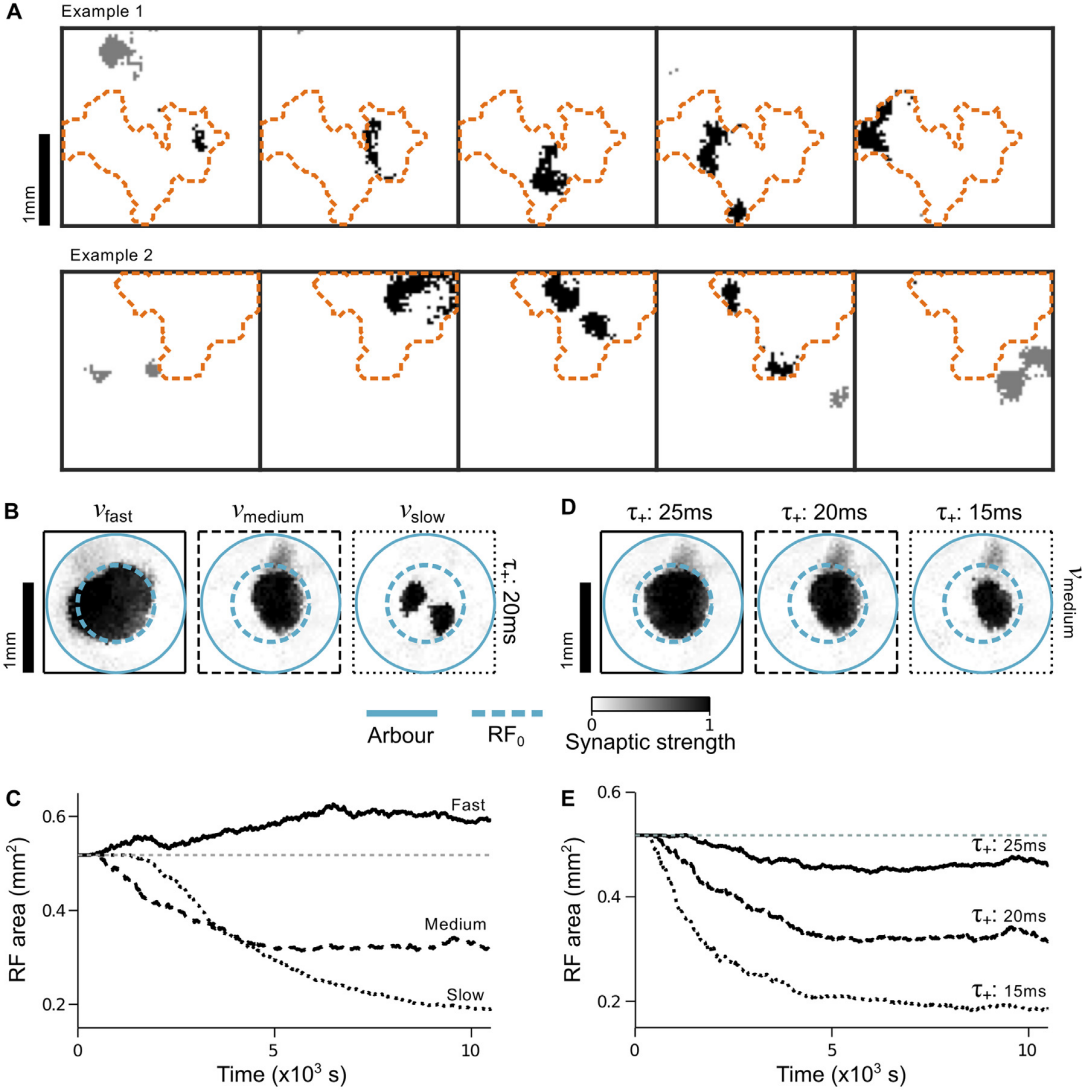
Refinement of RFs by complex wave patterns. **A**. Snapshots of two example waves generated by the complex wave model, with time increasing from left to right in steps of 0.4 s. Dashed orange curves mark the spatial boundaries within which activity was assigned to the same, isolated wave. Black pixels mark input neurons that fired at least one spike during a 0.1 s interval and were part of the isolated wave. Grey pixels mark input neurons that fired at least one spike but were not part of the isolated wave. The analysis of each isolated wave excluded activity from outside the orange boundary. **B**. Example final RFs that developed under different wave speeds, holding the asymmetric rule fixed with *τ*_+_ = 20 ms. Left: fast wave speeds; middle: medium wave speeds; right: slow wave speeds. Solid teal circles denote the boundary of the arbor function. Dashed teal circles denote the initial RF size. **C**. Summary of the development of RF areas under the influence of fast (solid black line), medium (dashed black line) and slow (dotted black line) waves, averaged over three trials. Dashed grey line: initial RF area. RF areas were calculated by multiplying the number of synapses with strength > 0.5 by the input cell spacing (34 *μ*m) squared. **D**. Example final RFs that developed under different time scales of an asymmetric STDP rule, holding the wave speed fixed with medium speed waves. Left: *τ*_+_ = 25 ms; middle: *τ*_+_ = 20 ms; right: *τ*_+_ = 15 ms. Teal circles the same as in **B**. **E**. Summary of the development of RF areas under the influence of different STDP time constants, averaged over three trials. Solid black line: *τ*_+_ = 25 ms; dashed black line: *τ*_+_ = 20 ms; dotted black line: *τ*_+_ = 15 ms. Dashed grey line: initial RF area.

To test the robustness of refinement under conditions in which waves are far from idealized plane waves moving with a constant velocity, we used a wave model developed by Feller et al. [39] to generate complex wave patterns in the input layer. In the Feller et al. model, waves are generated spontaneously in random locations, and propagate along winding trajectories on a 2D input layer (see Methods). Due to the complexity of the model, it was not possible to set a precise wave speed. We therefore controlled the mean wave speed by temporally rescaling precomputed wave patterns, and measuring the speeds of waves that were isolated by a center of mass (COM) tracking algorithm (Methods and S7 Fig). In this way, we generated slow, medium and fast waves with speeds of 1.29±0.01 mm/s, 2.58±0.02 mm/s and 3.87±0.03 mm/s (mean ± SEM), respectively. Examples of two isolated waves are shown in Fig 10A, and a 90 s movie of isolated waves is provided in S1 Movie.

Using these rescaled wave patterns as input to simulations of RF development, we found that, quite remarkably, complex waves could shape and refine RFs in much the same manner as simple plane waves. With an asymmetric STDP rule and holding *t*_+_ = 20 ms fixed, RFs could be made to expand (Fig 10B, left), contract (Fig 10B, center) or split into subfields (Fig 10B, right) by the fast, medium and slow complex waves, respectively. Although slow waves did not always split the RF, the final RF area was always smaller with slow waves than with medium wave speeds. The evolution of the RF area during the simulation, averaged over repeated trials, is plotted in Fig 10C for the different wave speeds (solid: fast waves; dashed: medium waves; dotted: slow waves). Similarly, STDP rules with shorter STDP time scales produced RFs with smaller areas (Fig 10D and 10E), corresponding to a shorter characteristic wavelength in a periodic connectivity pattern. These results recapitulate the dependence of RF development on wave speed and STDP time scales, even when the input wave patterns followed complex and noisy trajectories.

## Discussion

Here we examined how spatiotemporal correlations in traveling waves, a prominent feature of activity in the developing nervous system, could in principle interact with Hebbian STDP to instruct neural circuit development. Building upon previous studies of the interaction of STDP with correlated activity [16–21], we have identified a novel process by which traveling waves are able to establish and refine highly structured connectivity patterns in neural circuits. Specifically, our analysis has led to the following major results: 1) common examples of experimentally observed STDP build periodic patterns into feedforward networks when driven by traveling waves; 2) the spatial frequency of the periodic pattern scales with the wave speed and characteristic time scale of the STDP rule; 3) robust periodic connectivity patterns are produced only when the duration of the wave-related burst falls within a range that depends on the time scale of the STDP rule; the longer the burst duration, the longer the time scale of STDP must be to yield robust periodic patterns; 4) periodic patterning is robust to variability in the wave speed and wave frequency, but is lost if the ratio of non-wave spikes to wave-spikes becomes too high; 5) the shape and size of a RF can be modified by the spatial frequency imposed upon it by the wave-STDP interaction. Below, we discuss in greater detail how our results build upon previous work, and reflect on their application to experimentally observed wave phenomena and circuit refinement. We point out predictions from our model that may be tested using existing experimental paradigms, and suggest how our results may be extended in future work.

### Pattern formation in the brain and biology

Our theoretical results, expressed in Eqs 6 and 9, are analogous to those derived by Swindale [35], who modeled the development of ocular dominance columns in primary visual cortex. Swindale showed that short range excitatory and long range inhibitory lateral interactions could give rise to the spatially periodic dominance of eye specific afferents across primary visual cortex, a principle also used to model the development of orientation columns in visual cortex [57]. In our model, the lateral interactions between synapses that are necessary for pattern formation result from the timing dependence of STDP, which is mapped onto space by the spatiotemporal correlations of traveling waves. Pattern formation of this kind is analogous to a Turing instability in reaction-diffusion systems [29], which has been applied to diverse cases of biological pattern formation [31]. A useful recipe for Turing-like pattern formation includes the presence of a diffusible activator and inhibitor, whereby the inhibitor diffuses over greater distances than the activator [30]. STDP rules emulate this feature if *τ*_−_ > *τ*_+_, i.e. the temporal window for synaptic weakening is longer than that for synaptic strengthening. Such STDP rules are widely reported throughout the brain [14, 58]. In the alternative scenario, when *τ*_+_ > *τ*_−_, we found that STDP tends to weaken local regions of synapses that had strengthened by chance, preventing islands of strong synapses from forming. It is not impossible, however, for periodic patterns to form when *τ*_+_ > *τ*_−_, so long as we carefully choose *A*_+_ and *A*_−_, which control the overall bias for strengthening and weakening. However, we found that the stability of the pattern is very sensitive to small changes in *A*_+_ and *A*_−_ in this case.

### Influence of input correlations and synaptic competition on refinement

Models of circuit development by Hebbian plasticity require constraints on the synapses so as to keep their strengths within biologically realistic bounds. A suitable choice of constraint, such as subtractive normalization, provides competition between synapses so that when a subset of synapses strengthen, all other synapses are suppressed [11, 59, 60]. Under this constraint, correlations between nearby inputs over short temporal windows encourage a localized group of synapses to strengthen, yielding a RF-like connectivity pattern [11]. This is because synapses separated by larger distances are uncorrelated within short temporal windows, so are rarely strengthened but are often weakened together. STDP rules that are biased for synaptic weakening can achieve this type of competition between uncorrelated synapses, and are therefore capable of building RF-like connectivity patterns [18]. However, this competitive process is fundamentally different to the mechanism by which STDP builds structured connectivity patterns in this paper. We recognize that a single wave can induce strong correlations between any two input neurons that it passes, irrespective of their separation. They will be correlated with a specific time lag, Δ*t*, which scales with the separation, Δ*x*, between the two inputs as Δ*x* = *v*Δ*t*. If Δ*t* corresponds to synaptic weakening in the STDP rule, the synapses of the two input neurons will compete as a result of their strong correlations. Note that waves cannot produce periodic connectivity patterns if synaptic weakening does not have a particular dependence on timing, for example when competition is provided by subtractive normalization, as this will not yield the bandpass filtering that we have demonstrated for experimentally observed STDP rules (Fig 2B). This underlies why the wave speed, and the range of Δ*t* values for which the STDP rule specifies synaptic weakening or strengthening, determines the resulting spatial frequency of the connectivity pattern, and therefore influences the type of modification experienced by a RF.

In Fig 5, we showed how contributions to the correlation function that are not wave-related act to degrade the periodic connectivity pattern. The strength of wave-related correlations may be further suppressed by recurrent inputs arriving from other postsynaptic cells, which we have excluded from our model. Output spikes that are driven by recurrent activity are likely to be much less correlated with spikes in the input layer and will therefore contribute an approximately constant term to the correlation function, *C*(*x*,Δ*t*), the effect of which will be to emphasize any bias in the STDP rule towards synaptic weakening or strengthening. Moreover, excitatory recurrent connections are well known for encouraging similarities between postsynaptic RF properties, whereas inhibitory connections are known to decorrelate RFs, without drastically changing the basic RF properties of individual postsynaptic neurons [18, 57, 61–63].

Increasing the bias for synaptic weakening in our simulations enhanced the degree to which RFs could be refined to a smaller size (Fig 8E). A bias for weakening in STDP is frequently observed in experiments [14, 64], but is not the only possible source of synaptic competition, which can also be achieved by homeostatic regulation and intrinsic plasticity [65, 66]. As mentioned above, these additional sources of competition would not aid pattern formation if they do not have the appropriate spike timing dependence that yields bandpass filtering as do the STDP rules used in this study.

### Previous STDP models with space-time inseparable inputs

Several studies have previously explored the interaction between space-time inseparable input patterns and STDP. They demonstrated how motion stimuli can act through asymmetric STDP rules to impart an asymmetry in the spatial profile of excitatory connection strengths. This can be utilized to endow a network with direction selectivity similar to that found in the visual cortex [25–28]. Through our analytical results, we have shown that this phenomenon is one component of a richer set of dynamics with which the connectivity pattern can evolve under space-time inseparable inputs. Specifically, synaptic strengths are modified according to the convolution of the spatially mapped STDP rule, *κ*(*x*), with the synaptic strengths, *w*(*x*). Because of this convolution, the imaginary component of $\tilde{\kappa }(k)$ results in spatial shifts, whereas the real component results in the emergence of a periodic connectivity pattern. It is possible that periodic patterning was occluded in previous modeling studies due to the setup of those models for the specific application to the development of direction selectivity.

### Patterning and refinement by retinal waves

Retinal waves have been reported in the developing retina of several species [4, 67–71], suggesting that they play an important developmental role that has been conserved over the course of evolution. In mice, for example, disrupting normal retinal wave propagation [42, 72] has a profound effect on the refinement of retinal ganglion cell (RGC) afferents to, and RFs in, superior colliculus (SC) [73, 74]. One way in which retinal waves may drive this refinement is by imposing a wavelength onto the spatial structure of retinocollicular connections. To illustrate the relationship between RF sizes in SC, retinal wave speeds and STDP time scales, we computed the landscape of dominant spatial frequencies (S5 Fig) as a function of *v* and *τ*_+_, as in Fig 3, but using a typical retinal wave burst duration of 2 s for *α*(*t*) [42, 43]. Retinal waves travel at relatively slow speeds of 0.1–0.2 mm/s, such that if the STDP rule at retinocollicular synapses is asymmetric with *τ*_+_ = 20 ms, the characteristic wavelength would be 1/k* ≈ 0.09 mm (solid red rectangle in S5 Fig). By interpreting a RF as half a cycle in the periodic pattern, a wavelength of 0.09 mm corresponds to a RF size in mice of 1.8°, using the distance to visual angle conversion in Methods. This is much smaller than the reported RF sizes of ∼10° [74]. In order to obtain RFs ∼10° in diameter (equivalent to a wavelength of 0.51 mm) with slow retinal waves, the STDP time scale would have to be *τ*_+_ = 0.15 s (dashed red rectangle in S5 Fig), almost an order of magnitude longer than time scales commonly reported in STDP studies. The long duration of retinal wave bursts imposes an additional constraint on the minimum STDP time scale. We showed in Fig 4 that, when *τ*_+_ = 20 ms, periodic patterning degraded when the burst duration exceeded 0.5 s. However, as the STDP time scale increases, so too should the limiting burst duration (Fig 4F). Assuming that the limit imposed by the burst duration scales linearly with *τ*_+_ suggests that a STDP time constant exceeding 0.1 s should operate at retinocollicular synapses during retinal waves.

Our prediction of long STDP time scales at developing retinofugal synapses supports previous work by Butts and Rokhsar [41], who showed that retinal waves convey the most information about the relative retinotopic positions of RGCs over time scales ranging 0.1–2 s. This was further supported by the observation of such a rule at developing retinogeniculate synapses in the rat [44]. Evidence for long STDP time scales was recently provided in the visual cortex of mice that had a greater than normal expression of NMDA receptors (NMDARs) with NR2B subunits [75]. The STDP rule in these animals was temporally asymmetric, but was sensitive to remarkably long spike time differences greater than 0.1 s. NR2B expression is high during the stage of development when retinal waves are important for refinement, which in mice is the first postnatal week [52]. Thereafter, the number of NR2B containing NMDARs reduces, being replaced by faster acting NR2A containing NMDARs [76, 77]. The change in NMDAR composition may explain why many STDP studies in mice, which are typically conducted in the second or third postnatal week of development, reveal STDP rules with time scales not much longer than 20 ms. We speculate that such developmental changes in NMDAR composition may provide a gradual reduction in STDP time scales that enables greater levels of refinement, as we demonstrated in Fig 8B-8C. The effect of this developmental progression may be complemented by the observed reduction in wave speeds in mice during the same period [43]. Our results describe a potentially important role for these phenomena in refinement, which can be examined experimentally.

The noisy mechanism of wave generation in the developing retina adds considerable variability to the size of retinal waves [39]. In our model, it is important that the total distance travelled by a wave exceeds the characteristic wavelength, 1/k*. This is the spatial analog of requiring the wave duration to exceed the time scales of STDP in our analytical derivation. Our calculation above therefore suggests that retinal waves should travel distances greater than 0.51 mm in order to produce RFs 10° in diameter in the mouse SC. By tracking the center of mass of experimentally recorded retinal waves, Maccione et al. showed that a substantial majority of retinal waves indeed exceeded this distance throughout the period of major retinocollicular refinement [43].

Taken together, experimentally recorded retinal waves exhibit many of the properties required for retinocollicular refinement by a periodic patterning process. However, whether the mode of synaptic plasticity at retinocollicular synapses is suitable for this process has yet to be determined.

### Suitability of traveling waves for refinement in other brain areas

Traveling waves occur in many different parts of the brain, and each location will have particular constraints that limit the types of patterns that could form based on the interaction with STPD rules. As discussed above, one constraint is the characteristic wavelength, 1/k*, which sets a lower bound to the distance traveled by waves if periodic patterning is to be achieved. We computed the characteristic wavelengths associated with waves in brain areas other than the retina—including the cortex, cerebellum and hippocampus—and list them in Table 1, under the assumption of an asymmetric STDP rule like that used in many of the results above (*τ*_+_ = 20 ms), and which is reported widely throughout the brain. The speeds of waves in these areas are 1–2 orders of magnitude faster than retinal waves [5, 8, 9], but yield characteristic wavelengths that are similar in scale to that required for RFs in SC because of the short STDP time scale (first four entries in Table 1). Fast wave speeds and short STDP time scales place these waves in a region near the bottom right hand corner of Fig 3A (solid red rectangle), assuming a burst duration of 0.1 s. Also listed in Table 1 are the maximum distances over which the waves in different areas may propagate. Comparing these distances with the characteristic wavelengths reveals that periodic patterning in the efferents of the cerebellum, dorsal cortex and ventral cortex would not be expected, as the maximum possible propagation distance is close to or less than the lower bound set by 1/k*. However, wave properties in the hippocampus, as in the retina, do satisfy the minimum distance constraint for the type of STDP rule assumed. More detailed analyses of waves in the hippocampus and STDP at its efferent synapses is necessary to determine whether these phenomena could drive periodic patterning in a manner useful for development.

**Table 1:** Summary of different wave phenomena. Wavelengths, 1/k*, were computed using the wave speeds given, a burst duration of 0.1 s, and the same asymmetric STDP rule for the first four rows, with *τ*_+_ = 20 ms, *τ*_−_ = 40 ms, *A*_+_ = 1.0 and *A*_−_ = 0.51. For retinal waves in the last row, we used a burst duration of 2 s, and an asymmetric STDP rule with *τ*_+_ = 0.2 s, *τ*_−_ = 0.4 s, *A*_+_ = 1.0 and *A*_−_ = 0.55.

**Brain area**	**Wave speed (mm/s)**	1/k* (mm)	**Max. propagation distance (mm)**
Cerebellum [8]	3	0.8	1
Dorsal cortex [9]	17	4.8	3
Ventral cortex [9]	7	1.9	2
Hippocampus [5]	8	2.2	5
Retina [39]	0.15	0.5	3

### Development of cortical receptive fields

RFs of simple cells in the primary visual cortex (V1) exhibit weak selectivity for orientated features in the visual environment at the onset of vision [78–80] and become more selective with visual experience [79, 81]. Yet when an animal is raised in an environment comprising a restricted range of oriented contours, RF selectivity matures for those same orientations but not others [79–84]. A key feature of orientation tuned simple cell RFs is their composition of ON and OFF subfields, which are respectively sensitive to increments and decrements in luminosity, and are thought to reflect the visuotopic organization of ON and OFF inputs from the lateral geniculate nucleus (LGN) [85, 86]. The orientation of these subfields is thought to confer the orientation preference of the simple cell [85, 86]. However, it is not clear whether oriented features in the environment instruct the development of orientation selectivity, or whether they permit the maturation of RFs that are already selective for the same orientations. The spatial frequency tuning of simple cells may help to resolve this question, as it is well predicted by the periodic structure of ON and OFF subfields [87]. One model of simple cell RF development [57] posits that spatial frequency tuning is determined by the structure of spatial correlations between LGN cells. However, the necessary correlations were not observed in the developing LGN in later experiments [88]. Other models inspired by sparse coding schemes [89] posit that simple cell RFs result from learning the independent components of natural visual scenes [90], a consequence of which is that RFs would exhibit tuning for velocity and spatial frequency with an inversely proportional relationship [91]. However, despite recent insights [23, 63], it is not yet well established how sparse coding is implemented in biological circuits.

Our results provide an alternative mechanism for simple cell RF development. We have shown how oriented wavefronts can build orientated RFs (Fig 9A), and how the same process can develop multiple, periodic subfields (Fig 7 and S4A Fig), akin to the ON and OFF subfields of simple cells, thus providing a means for spatial frequency tuning (Fig 8). Moreover, our prediction of an inverse relationship between the wave speed and spatial frequency of the connectivity pattern provides a novel test for the role of visual experience in shaping simple cell development, and bares striking similarity to the sparse coding schemes mentioned above. Using stimuli that consist of high contrast luminance contours to elicit wave-like activity in the retina and LGN may help to test our predictions, and experimental protocols using such stimuli with young animals are already well established (for example, see [92]).

We can use our model to predict a range of plausible STDP parameters that would build RFs with the spatial frequency tuning of simple cells. In adult cats, spatial frequency tuning ranges from ∼0.2–2 cycles/° within eccentricities of ±15° [54, 93]. We further constrain the model by considering wave speeds that match the velocity tuning of cat simple cells (∼0.5–20°/s, [94]). In S6 Fig, we illustrate the spatial frequencies that are obtained in selected parts of this large STDP parameter space. Assuming an asymmetric STDP rule in the cortex [36, 95], realistic spatial frequencies can be obtained with STDP time scales ranging ∼1–100ms, and biases for synaptic weakening in the approximate range 0.3 ≲ *A*_-_/*A*_+_ ≲ 0.7. Realistically, STDP need not operate over such a wide range of parameters, as variability in the speeds of natural stimuli should explain most of the variability in spatial frequency tuning.

The relationship between temporal correlations and the development of spatially structured connectivity patterns may be extended to other visual RF properties. For example, temporal delays between inputs with spatially offset RFs are the major components needed to build direction selective cells [96, 97]. This kind of organization, and hence direction selectivity, can be learned with rate-dependent Hebbian plasticity [62], utilizing diverse response latencies in the LGN [98–100]. It is feasible that STDP should also yield spatial offsets between inputs with different response latencies, given its Hebbian nature. Combined with the capacity for periodic patterning, we speculate that the interaction of traveling waves with STDP could yield connectivity patterns that are direction selective, as well as orientation and spatial frequency tuned.

### Tolerance to background spiking noise

We found that pattern formation in our simulations was sensitive to noise: patterns began to degrade when the ratio of the background spike rate to the wave-induced spike rate became too high. It is therefore essential to consider what is known about background firing rates reported in the literature. We concentrate on background noise in the developing visual system, for which good data are available. During retinal wave activity in mice, retinal ganglion cells rarely spike outside of a burst. However, bursts that occur outside of a wave event have approximately the same firing rate as wave related bursts (Table 1 in [42]). Nevertheless, wave related bursts comprise approximately 90% of all bursts in the developing retina [42], suggesting that background spiking noise would have little impact on wave-induced correlations at this stage of visual development. Retinal waves also drive bursts in the LGN at firing rates of 10 Hz with long intervening periods of quiescence [101]. In later stages of development, visually evoked responses in the LGN reach firing rates of 10 Hz, whereas 1 Hz firing rates are typical of spontaneous activity [102]. This highly skewed distribution of firing rates is recapitulated in the developing visual cortex [103]. Thus, it is likely that, during early developmental periods when RF properties undergo refinement, spontaneous spikes contribute little to the overall activity in the visual system, and that patterned retinal waves and visual stimulation are propagated throughout the early visual system.

### Modulation of STDP by other factors

The precise dependence of STDP on spike timing can be sensitive to many factors that can modify the shape of the STDP rule (reviewed extensively in [14] and [58]). Whatever the mechanisms that underly these additional interactions, they must preserve a bandpass dependency on spike times in order for periodic patterns to form. Biophysical models of cellular activity that may underlie STDP have been proposed to account for some of these additional interactions [104, 105]. These models rely on the summed pre- and postsynaptic contributions to the intracellular calcium concentration, the amplitude of which at any given time determines whether synapses are strengthened or weakened. As such, the shape of the STDP rule changes for different spike trains and thus would not adhere to the spike-timing dependence that is necessary for bandpass filtering and pattern formation. If pattern formation as outlined in this paper does influence circuit development, then we predict that STDP must maintain a bandpass profile at synapses that mediate wave-like activity patterns. To test this, a typical STDP experimental protocol could be performed, in which paired spike bursts are stimulated in connected pre- and postsynaptic cells over a range of temporal offsets, and the resulting change in synaptic strength measured. A key requirement of such an experiment would be to extend the temporal offsets well beyond the burst durations, in contrast to previous studies [44, 106, 107], so as to detect the full temporal profile of the plasticity rule and ensure that synaptic changes decay to zero with larger offsets. If the bandpass property of synaptic plasticity is present at these synapses, then it should be reflected in the resulting STDP curve. Retinocollicular or geniculocortical synapses would be ideal substrates for testing this.

### Future directions

The robustness of our results to various types of noise suggests that pattern formation should also be achieved in more complex models that incorporate details specific to the circuit. The developing visual system provides a promising arena in which to test our theory of pattern formation for the reasons discussed above, and we outline here future work that will facilitate this investigation.

The dominant spatial frequency that characterizes a periodic pattern is strongly influenced by the stimulus response properties of the input neurons, as demonstrated by the effect of the burst duration in Fig 4. Incorporating accurate space- and time-dependent response properties of neurons in the visual pathway, as determined by their spatiotemporal RFs [55, 108–111], is therefore essential to making accurate predictions about pattern formation in downstream visual targets such as the SC and V1. For theoretical analysis, these features can be easily incorporated by reformulating *α*(*t*) as *α*(*x*,*t*) to take account presynaptic RF structures.

Application of our model to simple cell RF development will require modeling both ON and OFF response types in the input layer, which must become spatially segregated as a result of learning. Previous proposals for the mechanism of this segregation typically rely on correlations within each ON or OFF population exceeding those between the two populations [20, 57, 88]. The extent to which STDP will be sensitive to ON and OFF correlations, in addition to the strong spatiotemporal correlations induced by traveling waves, requires further investigation.

The linearity of our current model, though useful for theoretical analysis, prevents RFs from developing an orientation preference when waves travel in multiple directions (Fig 9B), as is the case for retinal waves and natural visual environments. Any bias towards one orientation is eventually averaged away by the influence of waves with other orientations. The addition of a nonlinearity, either in the sensitivity of STDP to the output firing rate, or in the transfer function for the output firing rate itself, should help to break the symmetry in wave directions and bias the RF towards any asymmetric shape that is built into it by chance. In this way, a population of output neurons might be able to acquire different orientation preferences when exposed to the same input pattern. In a similar fashion, a spectrum of spatial frequency preferences could be acquired throughout the population, rather than the uniform preference for a single spatial frequency, as we observed in simulations in which the wave speed was varied.

It is worth noting that traveling waves are just one incarnation of activity patterns that exhibit the space-time inseparable correlations necessary for periodic patterning. Our results may be generalized to any reliable temporal sequence of activity, some examples of which include navigation codes in the hippocampus [112], pre-motor coding in songbirds [113], and spontaneous ‘synfire chains’ in the cortex [114].

## Methods

### Overview

We model a reduced feedforward network consisting of a presynaptic layer of input neurons and a single postsynaptic output neuron. Traveling waves of activity traverse the input layer and recruit input neurons, which discharge bursts of spikes and provide excitatory synaptic inputs to the output neuron. Each feedforward synapse is modified according to the time delay between spikes from its corresponding input neuron and spikes from the output neuron. A schematic of the network is provided in Fig 1A.

### Spiking neuron simulation

Our model is simulated using a time step of 1 ms. During each step, a number of processes are simulated. First, input neurons generate spikes from one of two wave models described below. Each input spike elicits in the output neuron an excitatory postsynaptic potential (EPSP), which is weighted by the synaptic strength. EPSPs modulate the output membrane potential, which determines the generation of output spikes according to either a linear or nonlinear process, described below. Once all spikes have been generated for a given time step, synaptic strengths are modified by a STDP rule.

#### Traveling waves in the input layer

*1D and 2D plane waves*

The input layer comprised point neurons, arranged 20 *μ*m apart in a 1D chain or 2D square lattice. Plane waves were always initiated at one edge of the input layer and travelled the entire length to the opposite edge. For most simulations using plane waves, a blank period lasting at least 5 s was inserted between the end of one wave and the beginning of the next so as to allow the postsynaptic activation to decay to zero before the next wave traversed the inputs. The only simulations in which waves were not interleaved with a blank period were those in which the presence of multiple waves was tested.

In most 1D simulations, the wave direction was alternated between each wave. In simulations with multiple waves, the wave direction was alternated after every 100 waves. In the 2D plane wave simulations, the wave direction was selected randomly out of *N* possible directions that were equally spaced around the compass, where *N* was either 2, 4, 6 or 16.

When an input neuron is recruited by the wavefront, it undergoes a burst of spikes. The input firing rate during a burst, *α*(*t*), is modeled as a boxcar function: *α*(*t*) = *R*_in_[*H*(*t*)−*H*(*t*−*d*)], where *H*(*t*) is the Heaviside step function, *R*_in_ is the input firing rate, and *d* is the burst duration. During an input burst, spikes were generated in time steps of 1 ms using a Bernoulli process. Unless otherwise stated, *R*_in_ = 50 Hz.

In some simulations, input neurons were given background firing rates, $R_{\text{in}}^{\text{bg}}$, which are stated in the relevant results sections. In these simulations, the background rate was not added to the firing rate of inputs undergoing a wave-related burst. Otherwise, when an input neuron was not being recruited by a wave, it was silent.

In all cases, we repeated trials of plane wave simulations with the same model parameters 16 times, using different seeds for the random number generators, such that each trial would produce different spike trains. In simulations where a parameter that was normally fixed was instead a random variable, such as the wave speed, or wave direction in the case of 2D plane waves, the random number generator for that random variable was reseeded for repeated trials. Otherwise, the random number generator for spike generation was reseeded. In all cases, original spike sequences were always generated for each trial.

*Complex wave patterns*

To generate more complex wave patterns in 2D, we implemented a simulation based on a previously published model of spontaneous depolarizations that propagate across the developing mammalian retina [39]. In short, cells in the model that undergo spontaneous depolarizations provide excitatory input to nearby cells, triggering cascades of excitatory synaptic transmission that propagate throughout the network, while strong refractory currents quickly return active cells to the rest state. As such, wavefronts of activity propagate along winding trajectories between refractory domains of the network.

The model comprises a layer of starburst amacrine cells (SACs) and a layer of retinal ganglion cells (RGCs). Both layers are arranged on 64×64 square lattices with a cell spacing of 34 *μ*m. SACs receive excitatory synaptic input of equal strength from other SACs that are within a radial distance of 120 *μ*m. RGCs also receive excitatory input from SACs over the same distance but do not provide synaptic input to other RGCs or SACs. None of the synapses within the model retina undergo synaptic plasticity.

Every cell in the retina model is characterized by a dynamic excitation variable, *X*_*i*_, which is analogous to its membrane potential, for *i* = 1…*N* where *N* is the number of SACs or RGCs. The simulation evolves in time steps of 100 ms and *X*_*i*_ is updated for cells in the SAC (S) and RGC (R) layers according to

$$\begin{array}{c} \frac{d}{dt}X_{i}^{S}=n_{i}^{S}-\frac{X_{i}^{S}}{\tau^{R,S}}, \end{array}$$ (15)

$$\begin{array}{c} \frac{d}{dt}X_{i}^{R}=n_{i}^{S}-\frac{X_{i}^{R}}{\tau^{R,S}}, \end{array}$$ (16)

where $n_{i}^{\text{S}}$ is the number of active SACs at time, *t*, that are connected to cell *i* (from a maximum of 38 connected SACs). Thus, active SACs increase the excitation of connected SACs and RGCs. In the absence of input from any SACs, the excitation variables for both SACs and RGCs decay exponentially with a decay constant of *τ* ^R,S^ = 0.1 s. A SAC becomes active when $X_{i}^{\text{S}}$ exceeds a threshold, *θ*_S_ = 6. Similarly, RGCs become active when $X_{i}^{\text{R}}$ exceeds a threshold, *θ*_R_ = 10. Synaptic transmission from a SAC persists for 1 s, after which the SAC becomes refractory. Every time a SAC becomes refractory, the refractory period is chosen from a normal distribution with mean *τ*_ref_ = 40 s and standard deviation *σ*_ref_ = 20 s. After the refractory period, $X_{i}^{\text{S}}$ is set to zero. To model the spontaneous initiation of waves, SAC activation variables, $X_{i}^{\text{S}}$, were forced to exceed *θ*_S_ at each time step with a probability of 0.0035.

There are several possible ways to manipulate the wave speed in the wave model by tuning different parameters [39, 115]. However, this approach has the undesirable effect of changing the overall spatiotemporal structure of the waves, which complicates the interpretation of the effect wave speed has on receptive field development. In order to manipulate the wave speed without altering the wave patterns, we simulated long periods of wave activity and rescaled these activity patterns in time by a factor, *F*_t_. This involved recording the set of times, 𝓣_*i*_, at which the RGC activation, $X_{i}^{\text{R}}$, exceeded *θ*_R_. We then rescaled 𝓣_*i*_ by *F*_t_ to compress the wave patterns in time, and used the rescaled 𝓣_*i*_ as burst onset times for the *i* ^th^ RGC. RGC spike bursts 0.1 s in duration were then generated using a Bernoulli process with a firing rate of 50 Hz and temporal precision of 1 ms. The RGCs in this model retina act as the input neurons in the final set of 2D simulations in this paper. To produce slow, medium and fast waves, we used *F*_t_ = 0.21, 0.11 and 0.07, respectively. Thus, medium and fast waves were respectively two and three times faster than the slow waves.

We repeated trials of complex wave simulations with the same model parameters three times, using different seeds for the *τ*_ref_ random number generator, such that each trial would produce a different sequence of wave patterns.

#### Excitatory postsynaptic potentials

Each input spike elicits an EPSP in the output neuron. If at time, *t*_*j*,*n*_, input *j* fires its *n* ^th^ spike, then at some later time *t*, the resulting EPSP is described by a difference of exponentials:

$$\epsilon_{j,n}\left(t\right)=\frac{1}{\tau_{d}-\tau_{r}}[\exp(-\frac{\left(t-t_{j,n}\right)}{\tau_{d}})-\exp(-\frac{\left(t-t_{j,n}\right)}{\tau_{r}})],$$ (17)

where *τ*_*d*_ = 5 ms and *τ*_*r*_ = 1 ms are the EPSP decay and rise times, respectively, based on neurotransmission through AMPA receptors, and *ϵ*_*j*,*n*_(*t*) = 0 for *t* < *t*_*j*,*n*_.

#### Linear output neuron

Spikes in the linear output neuron are generated by a Bernoulli process: at time, *t*, the output fires a single spike with a probability that is determined by its time varying firing rate, *λ*(*t*), which is proportional to the summed EPSPs:

$$\lambda \left(t\right)=R_{\text{out}}\sum_{j}\sum_{n}w_{j}\left(t\right)\epsilon_{j,n}\left(t\right).$$ (18)

Here, *R*_out_ is a constant of proportionality, chosen to constrain the output neuron to realistic firing rates in the range 10–100 Hz, and *w*_*j*_ is the strength of the synapse from input neuron *j* at the time of its *n* ^th^ spike.

#### Nonlinear output neuron

We model a nonlinear output neuron with leaky integrate and fire (LIF) dynamics. The output neuron fires a spike at time $\hat{t}$ when its membrane potential, *u*(*t*), is pushed above a threshold, *ϑ*, by incoming EPSPs, *ϵ*(*t*). EPSPs follow Eq 17, which models the leaky response of *u*(*t*) to an inward synaptic current, *I*(*t*), that has an instantaneous rise and exponential decay: *I*(*t*) = *H*(*t*)exp(−*t*/*τ*_*r*_)/*τ*_*r*_, with *H*(*t*) the Heaviside step function. After an output spike, an instantaneous outward current, $-\vartheta \delta (t-\hat{t})$, returns *u*(*t*) to the resting potential, *u*_rest_ = 0. The full response of *u*(*t*) to incoming EPSPs and the outward current follows the equation

$$u\left(t\right)=\xi \left(t-\hat{t}\right)+\sum_{j}w_{j}\sum_{n}\epsilon_{j,n}\left(t-t_{j,n}\right),$$ (19)

where *ξ*(*t*) is a refractory kernel that models the leaky response of *u*(*t*) to the outward current. The kernel *ξ*(*t*) is therefore a decaying exponential that imposes a relative refractory period *ρ*_rel_ = *τ*_*d*_ = 5 ms. In addition, we incorporate into *ξ*(*t*) an absolute refractory period, *ρ*_abs_ = 2 ms, during which it is impossible for the output to spike. The full refractory kernel is therefore:

$$\xi \left(t\right)=\{\begin{array}{cc} 0 & \text{if}\;t<0\\ -\infty & \text{if}\;t\leq \rho_{\text{abs}}\\ -\vartheta \exp(-\frac{t}{\rho_{\text{rel}}}) & \text{if}\;t>\rho_{\text{abs}}. \end{array}$$ (20)

Note that *ξ*(*t*) depends only on the time since the last output spike, whereas EPSPs summate over all previous input spikes.

#### Synaptic plasticity

After the spike generating process, synaptic strengths are updated using a spike-timing dependent plasticity (STDP) rule. Two rules are used in this paper, one asymmetric [13], *K*_asym_, and the other symmetric [38] in time, *K*_sym_ (Fig 1C):

$$K_{\text{asym}}\left(\Delta t\right)=\{\begin{array}{cc} A_{+}\exp\left(\Delta t/\tau_{+}\right) & \text{if}\;\Delta t<0\\ -A_{-}\exp\left(-\Delta t/\tau_{-}\right) & \text{if}\;\Delta t>0\\ 0 & \text{if}\;\Delta t=0, \end{array}$$ (21)

$$K_{\text{sym}}\left(\Delta t\right)=[A_{+}\exp(-\frac{1}{2}{\left(\frac{\Delta t}{\tau_{+}}\right)}^{2})-A_{-}\exp(-\frac{1}{2}{\left(\frac{\Delta t}{\tau_{-}}\right)}^{2})].$$ (22)

Here, Δ*t* = *t*_in_−*t*_out_ is the time difference between spikes belonging to input and output neurons, respectively. The size of the temporal windows for synaptic strengthening and weakening are scaled by *τ*_+_ and *τ*_−_, respectively, and *A*_+_ and *A*_−_ scale the relative degree of strengthening and weakening, respectively. The size of synaptic modifications, or learning, is further scaled by *η*, which is set to be a small number. Every time an input (output) neuron spikes, all output (input) spikes in the previous 5*τ*_−_ seconds are used to update a synapse. Unless otherwise stated, all asymmetric STDP rules are constructed such that *τ*_−_ = 2*τ*_+_, and all symmetric STDP rules have *τ*_−_ = 1.6*τ*_+_. In the results, we therefore almost always refer to the STDP time scale using just *τ*_+_. Furthermore, unless otherwise stated, *A*_+_ = 1.0 and *A*_−_ = 0.51 for all asymmetric rules, whereas *A*_+_ = 3.2 and *A*_−_ = 2.1 for all symmetric rules. In the simulations with complex wave patterns, an asymmetric rule is used with *A*_+_ = 1.0 and *A*_−_ = 0.55.

To prevent synaptic strengths from increasing ad infinitum, or from becoming negative and thus inhibitory, both of which are biologically implausible scenarios, we imposed hard bounds at synaptic strengths of 0 and 1.

#### Initial synaptic strengths

At the beginning of every simulation, the synaptic strengths were initialized with one of two configurations: 1) all synapses were given a strength of 0.5; 2) the central synapses, spanning a distance of *RF*_0_, were given the maximum strength of 1.0, and all other synapses given strengths of zero, to simulate a coarse RF structure. When the synaptic strengths were initialized with a RF, an arbor function, *A*(*x*), was applied to maintain the RF structure and prevent a periodic connectivity pattern from emerging across all synapses. The arbor function was implemented by multiplying *A*(*x*) with ∂_*T*_ *w*(*x*), where

$$\begin{array}{ccc} A(x) & = & 1,\text{if}|x-x_{0}|\leq a_{0}\\ & = & 0,\text{if}|x-x_{0}|>a_{0}, \end{array}$$ (23)

where *a*_0_ is the radius of the arbor and *x*_0_ is the center of the arbor.

#### Local input correlations

In some simulations, we induced additional correlations between neighboring inputs by generating spikes in two stages for each time step. In the first stage, spikes were generated by a Bernoulli process as usual but with a modified input firing rate, ${\hat{R}}_{\text{in}}=R_{\text{in}}/\left(1+2c\right)$, where *c* is the correlation strength and takes values between 0 and 1. In the second stage, correlated spikes were generated with a probability, *c*, in all cells neighboring inputs that spiked in the first stage. In simulations with background firing rates, the background rate was likewise modified so that ${\hat{R}}_{\text{in}}^{\text{bg}}=R_{\text{in}}^{\text{bg}}/\left(1+2c\right)$. Dividing by the factor 1+2*c* in the first stage kept the firing rates constant after the second stage, allowing independent manipulation of the correlation strength.

#### Wave speed and inter-wave interval distributions

In simulations with variable wave speeds or IWIs, a new speed or IWI was drawn from a lognormal distribution for each wave. For the *i* ^th^ wave, the lognormal random variable, *y*, was:

$$y_{i}=e^{m+s\zeta_{i}},$$ (24)

where

$$m\left(\mu ,\sigma \right)=\ln(\frac{\mu }{\sqrt{1+\frac{\sigma }{\mu^{2}}}})$$ (25)

and

$$s\left(\mu ,\sigma \right)=\sqrt{\ln(1+\frac{\sigma }{\mu^{2}})}$$ (26)

are respectively the mean and standard deviation (SD) of ln(*y*), *μ* and *σ* are respectively the mean and SD of *y*, and *ζ* is a normally distributed random variable with zero mean and unit variance.

For simulations with variable speeds, *μ*_*v*_ = 4 mm/s was fixed and *σ*_*v*_ was varied between simulations. We imposed a minimum speed of 0.05 mm/s, such that the *i* ^th^ wave speed was:

$$v_{i}=\max(0.05,y_{i}).$$ (27)

For simulations with variable IWIs, the IWI distribution was modified slightly to ensure that IWIs were no shorter than the burst duration, *d*. That is, successive waves never overlapped. Each new IWI was therefore:

$${\text{IWI}}_{i}=d+y_{i},$$ (28)

where *y* was computed using *m*(*μ*−*d*,*σ*) and *s*(*μ*−*d*,*σ*). For these simulations, *σ* = 1 s was fixed, and *μ* was varied between simulations. Because a downtime of at least 5 s is needed for waves to clear the input layer before switching direction, we switched the wave direction in these simulations after every 100 waves. That is, ∼ 99% of the waves sampled from the IWI distribution.

### Analysis of complex wave patterns

To measure the speed and size of simulated complex wave patterns, we analyzed a 2000 s segment of RGC spiking activity from a simulation of the slow waves only, as these waves were less compressed in time and therefore lasted longer, enabling a more accurate measure of the wave properties. Spike times were first converted into a firing rate movie, *M*(*x*,*y*,*t*), with dimensions 64 × 64 × 20000, where the (*i*,*j*,*k*)^th^ bin in *M* contained the number of spikes fired by a single RGC, at location (*x*_*i*_,*y*_*j*_), during a 100 ms period, *t*_*k*_.

During the retinal wave simulation, multiple waves were occasionally present at the same time. Concurrent waves would occasionally collide into or split from each other, but they were mostly well separated in space. We sought to isolate concurrent waves to accurately measure their individual properties, such as speed and size, using a center of mass (COM) tracking algorithm, of which a schematic is drawn in S7 Fig. First, we computed a smoothed firing rate movie, *M*_s_(*x*,*y*,*t*) = (M*G)(*x*,*y*,*t*), where $G(x,y,t)=e^{\frac{x^{2}}{2\sigma_{x}^{2}}+\frac{y^{2}}{2\sigma_{y}^{2}}+\frac{t^{2}}{2\sigma_{t}^{2}}}$ is a Gaussian filter with a spatial standard deviation (SD), *σ*_*x*_ = *σ*_*y*_ = 34 *μ*m, and temporal SD, *σ*_*t*_ = 100 ms. After smoothing, pixel values below 10 were set to zero. Thus, a single time bin in *M*_s_ might have several domains of non-zero pixels. To track the COM of different waves, a boundary was drawn around each domain of activity in time bin *k* and the COM within the boundary calculated, where mass refers to the firing rates within the same boundary in *M*(*x*,*y*,*t* = *k*). Two domains, colored blue and orange, and their COMs (purple and green, respectively) are shown in S7 Fig, and the firing rates given in greyscale. Each domain was assigned a wave identification number (ID) but, if the COMs at two domains were less than 680 *μ*m apart, they were given the same ID. If the COM of one domain was within a 680 *μ*m radius from the COM of another domain in the previous time bin (purple lines extending from the COM in S7 Fig), it was given the same ID. In this way, wave COMs were tracked according to their ID. The COM trajectory of each wave was zero-padded and smoothed in time by a Gaussian filter with a SD of 0.1 s, and the first two and last two COM locations discarded. Thus, wave speeds and sizes were only computed for waves that lasted for more than 0.5 s. We used the path length of a COM trajectory as the distance travelled by a wave, and computed the wave speed from this by dividing it by the time taken to travel that trajectory.

RGCs near the edges of the model retina received fewer lateral inputs from SACs and were less active than those in the centre. Within the central 44 × 44 RGCs, the total level of spiking activity was comparably uniform. Accordingly, waves with a time-averaged centre of mass (COM) that resided in the outer 10 neurons were discarded from any further analysis. Occasionally, small segments of waves were isolated from larger waves. These were discarded from the analysis by removing waves that covered fewer than 1000 space-time bins.

The COM tracking algorithm performed well in separating waves that eventually merged with, or split from, other waves, and allowed us to perform analysis on almost every wave that had a unique ID by removing concurrent waves from that analysis. A total of 797 waves were isolated in the 2000 s segment, of which 779 lasted long enough to compute the wave speed and distance travelled.

All analysis was performed with Matlab R2012a using built-in and custom built functions.

### Computing numerical solutions

Concordance of the spiking model with analytical results is verified by numerically integrating Eq 6 using the forward Euler method. Synaptic strengths were initialized in the same way as in the spiking simulation. However, as there was no spiking noise when solving Eq 6 numerically, low amplitude Gaussian white noise was added to the initial synaptic strengths. To further align numerical integration with the simulations, nonlinearities that were present in the simulations were also incorporated in the numerical integration by: 1) restricting synaptic strengths to the range [0, 1]; 2) alternating the direction of the wave by replacing the wave speed, *v*, with (−1)^*n*^ *v* for the *n* ^th^ iteration; 3) applying the arbor function in Eq 23 when the initial condition of *w*(*x*) supported a RF structure. To prevent numerical solutions from becoming chaotic, the learning rate, *η*, was varied depending on the values of *τ*_+_ and *v*.

### Robustness measures

To measure the robustness, Ψ_*w*_, with which a periodic structure emerged in a connectivity pattern, we computed the discrete power spectrum, *P*(*k*_*i*_), of the connectivity pattern, with the DC component removed, and determined the ratio of the power at the dominant spatial frequency to the total power:

$$\Psi_{w}=\frac{P\left(k_{i}^{*}\right)}{\sum_{i=-k_{\text{N}}}^{k_{\text{N}}}P\left(k_{i}\right)},$$ (29)

where *k*_N_ is the Nyquist limit. As a predictor of robustness in the connectivity patterns, a theoretical robustness measure, Ψ_*κ*_, was computed for the power spectrum of the real component of $\tilde{\kappa }(k)$: $|\text{Re}[\tilde{\kappa }(k)]{|}^{2}$, which was obtained by first numerically computing *κ*(*x*) with a spatial resolution of 0.6 *μ*m. The theoretical robustness was therefore:

$$\Psi_{\kappa }=\frac{|\text{Re}\left[\tilde{\kappa }\left(k_{i}^{*}\right)\right]{|}^{2}}{\sum_{i=-k_{\text{N}}}^{k_{\text{N}}}{\left|\text{Re}\left[\tilde{\kappa }\left(k_{i}\right)\right]\right|}^{2}}.$$ (30)

### Converting between visual angle and retinal distance

To set up simulations of RF refinement, we use data from experimental studies regarding receptive fields, or traveling wave speeds, for example, which require conversion between degrees of visual angle and units of distance along the retinal surface. The precise conversion relationship between visual angle and retinal distance depends on the geometry of the eye, which differs for different animals. We therefore use a general conversion factor to provide a useful estimate. We assume that the retina covers two thirds of the circumference of a horizontal section through the center of the eye [116], and that this maps to 180° of visual angle. This means that distance in millimeters, *R*, corresponds to visual angle, *A*, according to *R* = *πrA*/135, where *r* is the radius of the eyeball. We used the following values for the radii of eyes in different animals at different stages of development: mouse, 1–7 days old: *r* ≈ 1.1 mm (Table 1 in [117]); mouse, adult: *r* ≈ 1.7 mm (Table 1 in [117]); ferret, eye opening: *r* ≈ 2.8 mm (Fig. 8 in [118]); cat, 4 weeks old: *r* ≈ 5 mm (Fig. 3 in [119]).

## Supporting Information

S1 Movie A 90 s extract of simulated slow waves demonstrate the performance of the COM tracking algorithm. Black and colored pixels correspond to RGCs that fired at least one spike in a 100 ms time bin. Each isolated wave is assigned a new color. Activity that was not assigned to any wave is in black.
(AVI)

S1 Text Full derivation of Eq 5 in the main text. (PDF)

S1 FigExamples of $\tilde{\kappa }(k)$ for three burst durations, *d* = 0.01 s (cyan), 0.1 s (orange) and 1.0 s (black), where *α*(*t*) has been modeled using a smooth function: $\alpha (t)=\frac{t}{d}e^{-t/d}$.All other parameters are the same as for Fig 4D. The power is distributed across a broad range of high frequencies for a 0.01 s burst, and within a small band of the lowest frequencies for a 1.0 s burst, yielding the strong negative lobe in the black curve. The negative lobe in the black curve extends beyond the horizontal axis and has been cut for clarity. However, for 0.1 s bursts, the power is concentrated in between these two extremes, around the dominant spatial frequency, k*.(TIFF)

S2 FigPredicted spatial frequencies as a function of the IWI for regular waves.Black circles: predicted spatial frequencies. Horizontal dashed grey line: predicted spatial frequency for a single wave in isolation. Vertical dashed grey line: critical IWI, IWI_crit_ = 1/vk*. Insets: examples of $\text{Re}\left[\tilde{\kappa }\left(k\right)\right]$ for different IWIs, including IWIs of 0.15 s (top left) and 0.2 s (second from top left). The dominant spatial frequency for regular waves varies around ∼ 0.91 cycles/mm, which is the dominant frequency for an isolated wave, as a result of different peaks in ${\tilde{\alpha }}_{\text{III}}(vk)$ being picked out by ${\tilde{K}}_{v}(k)$. When the IWI falls below IWI_crit_, the dominant frequency increases monotonically with decreasing IWI.(TIFF)

S3 Fig Emergence of the periodic connectivity pattern from an initial RF when the network has no arbor. (TIFF)

S4 Fig The structure of 2D RFs reflects the axes along which waves travel in the simulations. Top row: example RFs using wave speeds of 1 mm/s and *τ*_+_ = 20 ms. An arbor of 0.66 mm was used. Bottom row: 2D power spectra of the RFs with DC component removed, averaged over four repeated trials of the simulation, and normalized to the peak power. The orange circle denotes the predicted dominant spatial frequency for waves traveling in all directions. **A**. Waves traveling along the horizontal axis yield RFs that exhibit vertically aligned subfields and that are indicative of simple cell RFs in primary visual cortex. **B**. Waves travel along the horizontal and vertical axes. **C**. Waves travel along three equally spaced axes.
(TIFF)

S5 FigPredicted spatial frequencies as a function of the wave speed and STDP time scale for burst durations of 2 s.Solid black contours denote spatial frequencies equal to 10 raised to integer exponents. Spatial frequencies were obtained by locating the maximum in $\tilde{\kappa }(k)$ as a function of *v* and *τ*_+_, using an asymmetric STDP rule and a burst duration of 2 s for *α*(*t*). Sharp transitions in spatial frequency along the *τ*_+_ axis are due to *κ*(*x*) having several peaks of near equal amplitude (c.f. black curve in Fig 4D), such that small changes in *τ*_+_ can change which peak is the global maximum. Solid red rectangle: given a typical STDP rule with *τ*_+_ = 20 ms, the connectivity pattern associated with retinal wave speeds would have a dominant spatial frequency of ∼ 11 cycles/mm, or a wavelength of 0.9 mm. Dashed red rectangle: RFs in the SC require a characteristic wavelength of ∼ 0.51 mm, which corresponds to a spatial frequency of ∼ 2 cycles/mm. Given the speed of retinal waves, the required STDP time scale is predicted to be 0.1–0.2 s.(TIFF)

S6 FigSpatial frequency and velocity tuning of cat simple cells dictate a plausible range of STDP parameters at geniculocortical synapses.Using a burst duration of 100 ms, which matches the impulse response duration of immature LGN cells in the kitten [111], we computed a spatial frequency map as a function of the STDP decay times, *τ*_+_ and *τ*_-_, for four conditions: the STDP amplitudes were either *A*_−_/*A*_+_ = 0.3 (top row) or *A*_+_/*A*_−_ = 0.7 (bottom row), and the wave speed was either 3°/s (left column) or 10°/s (right column) [94]. We further restricted our analysis to STDP rules that had a reasonable bias for either weakening or strengthening by ignoring any rule for which the DC power exceeded that at the dominant spatial frequency, i.e. cases when $|\tilde{\kappa }{\left(0\right)|}^{2}>{\left|\tilde{\kappa }\left(k^{*}\right)\right|}^{2}$. We also restrict the spatial frequency maps to frequencies that lie in the range observed in adult cats: 0.2–2 cycles/° [54, 93].(TIFF)

S7 Fig Schematic for COM tracking algorithm. The two images depict activity that was generated with the complex wave model at time bins *k* (left) and *k* + 1 (right). The solid blue areas mark the domains in *M*_s_(*x*, *y*, *t*) that were assigned to one isolated wave, and the solid orange areas mark domains that were assigned to another isolated wave. Greyscale pixels illustrate the firing rates of RGCs in *M*(*x*, *y*, *t*) within each domain. Solid purple and green dots denote the COM for the first and second wave, respectively, in the current time bin. To illustrate how the COMs moved between time bins, the open purple and green dots (right) denote the COM of each wave in the previous time bin (left). Domains with COMs that are separated by less than 680 *μ*m (purple circle around the blue wave), in the same time bin or in adjacent time bins, are assigned to the same wave.
(TIFF)

## References

[1] Kolodkin AL, Tessier-Lavigne M. Mechanisms and Molecules of Neuronal Wiring: A Primer. Cold Spring Harb. Perspect. Biol. 2011; 3, a001727 10.1101/cshperspect.a001727 PMC3098670 21123392

[2] Katz LC, Shatz CJ. Synaptic activity and the construction of cortical circuits. Science 1996; 274, 1133–1138 10.1126/science.274.5290.1133 8895456

[3] Zhang LI, Poo MM. Electrical activity and development of neural circuits. Nat. Neurosci. 2001; 4, 1207–1214 11687831 10.1038/nn753

[4] Meister M, Wong RO, Baylor DA, Shatz CJ. Synchronous bursts of action potentials in ganglion cells of the developing mammalian retina. Science 1991; 252, 939–943 10.1126/science.2035024 2035024

[5] Leinekugel X, Khalilov I, Ben-Ari Y, Khazipov R. Giant depolarizing potentials: the septal pole of the hippocampus paces the activity of the developing intact septohippocampal complex in vitro. J. Neurosci. 1998; 18, 6349–6357 9698326 10.1523/JNEUROSCI.18-16-06349.1998 PMC6793205

[6] Garaschuk O, Linn J, Eilers J, Konnerth A. Large-scale oscillatory calcium waves in the immature cortex. Nature 2000; 3, 452–459 10.1038/74823 10769384

[7] Frechette ES, Sher A, Grivich MI, Petrusca D, Litke AM, Chichilnisky EJ. Fidelity of the Ensemble Code for Visual Motion in Primate Retina. J. Neurophysiol. 2005; 94, 119–135 10.1152/jn.01175.2004 15625091

[8] Watt AJ, Cuntz H, Mori M, Nusser Z, Sjöström PJ, Häusser M. Traveling waves in developing cerebellar cortex mediated by asymmetrical Purkinje cell connectivity. Nat. Neurosci. 2009; 12, 463–473 10.1038/nn.2285 19287389 PMC2912499

[9] Conhaim J, Cedarbaum ER, Barahimi M, Moore JG, Becker MI, Gleiss H, Kohl C, Moody WJ. Bimodal Septal and Cortical Triggering and Complex Propagation Patterns of Spontaneous Waves of Activity in the Developing Mouse Cerebral Cortex. Develop. Neurobiol. 2010; 70, 679–692 10.1002/dneu.20797 20506182

[10] Hebb DO. The organization of Behavior. New York: Wiley; 1949

[11] Miller KD, MacKay DJC. The role of constraints in Hebbian learning. Neural Comput. 1994; 6, 100–126 10.1162/neco.1994.6.1.100

[12] Markram H, Lübke J, Frotscher M, Sakmann B. Regulation of Synaptic Efficacy by Coincidence of Postsynaptic APs and EPSPs. Science 1997; 275, 213–215 10.1126/science.275.5297.213 8985014

[13] Zhang LI, Tao HW, Holt CE, Harris WA, Poo MM. A critical window for cooperation and competition among developing retinotectal synapses. Nature 1998; 395, 37–44 10.1038/25665 9738497

[14] Caporale N, Dan Y. Spike timing-dependent plasticity: a Hebbian learning rule. Annu. Rev. Neurosci. 2008; 31, 25–46 10.1146/annurev.neuro.31.060407.125639 18275283

[15] Gerstner W, Kempter R, van Hemmen JL, Wagner H. A neuronal learning rule for sub-millisecond temporal coding. Nature 1996; 383, 76–78 10.1038/383076a0 8779718

[16] Kempter R, Gerstner W, van Hemmen JL. Hebbian learning and spiking neurons. Phys. Rev. E 1999; 59, 4498–4514 10.1103/PhysRevE.59.4498

[17] Song S, Miller KD, Abbott LF. Competitive Hebbian learning through spike-timing-dependent synaptic plasticity. Nat. Neurosci. 2000; 3, 919–926 10.1038/78829 10966623

[18] Song S, Abbott LF. Cortical Development and Remapping through Spike Timing-Dependent Plasticity. Neuron 2001; 32, 339–350 10.1016/S0896-6273(01)00451-2 11684002

[19] Gütig R, Aharonov R, Rotter S, Sompolinsky H. Learning Input Correlations through Nonlinear Temporally Asymmetric Hebbian Plasticity. J. Neurosci. 2003; 23, 3697–3714 12736341 10.1523/JNEUROSCI.23-09-03697.2003 PMC6742165

[20] Gjorgjieva J, Toyoizumi T, Eglen SJ. Burst-time-dependent plasticity robustly guides ON/OFF segregation in the lateral geniculate nucleus. PloS Comp. Biol. 2009; 5(12): e1000618 10.1371/journal.pcbi.1000618 PMC2790088 20041207

[21] Gilson M, Burkitt AN, Grayden DB, Thomas DA, van Hemmen JL. Representation of input structure in synaptic weights by spike-timing-dependent plasticity. Phys. Rev. E 2010; 82, 021912 10.1103/PhysRevE.82.021912 20866842

[22] Clopath C, Büsing L, Vasilaki E, Gerstner W. Connectivity reflects coding: a model of voltage-based STDP with homeostasis. Nat. Neurosci. 2010; 13, 344–352 10.1038/nn.2479 20098420

[23] Savin C, Joshi P, Triesch J. Independent Component Analysis in Spiking Neurons. Plos. Comp. Biol. 2010; 6(4): e1000757 10.1371/journal.pcbi.1000757 PMC2858697 20421937

[24] Senn W, Buchs NJ. Spike-Based Synaptic Plasticity and the Emergence of Direction Selective Simple Cells: Mathematical Analysis. J. Comp. Neurosci. 2003; 14, 119–138 10.1023/A:1021935100586 12567013

[25] Buchs NJ, Senn W. Spike-Based Synaptic Plasticity and the Emergence of Direction Selective Simple Cells: Simulation Results. J. Comp. Neurosci. 2002; 13, 167–186 10.1023/A:1020210230751 12226559

[26] Rao RPN, Sejnowski T. Complex Cell-like Direction Selectivity through Spike-Timing Dependent Plasticity. IETE J. Research 2003; 49, 97–111 10.1080/03772063.2003.11416329 21057672 PMC2970931

[27] Wenisch OG, Noll J, van Hemmen JL. Spontaneously emerging direction selectivity maps in visual cortex through STDP. Biol. Cybern. 2005; 93, 239–247 10.1007/s00422-005-0006-z 16195915

[28] Honda H, Urakubo H, Tanaka K, Kuroda S. Analysis of development of direction selectivity in retinotectum by a neural circuit model with spike timing-dependent plasticity. J. Neurosci. 2011; 31, 1516–1527 10.1523/JNEUROSCI.3811-10.2011 21273436 PMC6623591

[29] Turing AM. The Chemical Basis of Morphogenesis. Phil. Trans. R. Soc. B 1952; 237, 37–72 10.1098/rstb.1952.0012

[30] Meinhardt H, Gierer A. Pattern formation by local self-activation and lateral inhibition. Bio. Essays 2000; 22, 753–760 10.1002/1521-1878(200008)22:8<753::AID-BIES9>3.0.CO;2-Z 10918306

[31] Kondo S, Miura T. Reaction-Diffusion Model as a Framework for Understanding Biological Pattern Formation. Science 2010; 329, 1616–1620 10.1126/science.1179047 20929839

[32] Fiete IR, Senn W, Wang CZH, Hahnloser RHR. Spike-time-dependent plasticity and heterosynaptic competition organize networks to produce long scale-free sequences of neural activity. Neuron 2010; 65, 563–576 10.1016/j.neuron.2010.02.003 20188660

[33] Blum KI, Abbott LF. A Model of Spatial Map Formation in the Hippocampus of the Rat. Neural Computation 1996; 8, 85–93 10.1162/neco.1996.8.1.85 8564805

[34] Mehta MR, Quirk MC, Wilson MA. Experience-Dependent Asymmetric Shape of Hippocampal Receptive Fields. Neuron 2000; 25, 707–715 10.1016/S0896-6273(00)81072-7 10774737

[35] Swindale NV. A model for the formation of ocular dominance stripes. Proc. R. Soc. Lond. 1980; B 208, 243–264 10.1098/rspb.1980.0051 6105656

[36] Feldman DE. Timing-based LTP and LTD at vertical inputs to layer II/III pyramidal cells in rate barrel cortex. Neuron 2000; 27, 45–56 10.1016/S0896-6273(00)00008-8 10939330

[37] Nishiyama M, Hong K, Mikoshiba K, Poo MM, Kato K. Calcium stores regulate the polarity and input specificity of synaptic modification. Nature 2000; 408, 584–588 10.1038/35046067 11117745

[38] Wittenberg GM, Wang SSH. Malleability of Spike-Timing-Dependent Plasticity at the CA3-CA1 Synapse. J. Neurosci. 2006; 26, 6610–6617 10.1523/JNEUROSCI.5388-05.2006 16775149 PMC6674029

[39] Feller MB, Butts DA, Aaron HL, Rokhsar DS, Shatz CJ. Dynamics processes shape spatiotemporal properties of retinal waves. Neuron 1997; 19, 293–306 10.1016/S0896-6273(00)80940-X 9292720

[40] Caporale N, Dan Y. Spike timing-dependent plasticity: a Hebbian learning rule. Annu. Rev. Neurosci. 2008; 31, 25–46 10.1146/annurev.neuro.31.060407.125639 18275283

[41] Butts DA, Rokhsar DS. The information content of spontaneous retinal waves. J. Neurosci. 2001; 21, 961–973 11157082 10.1523/JNEUROSCI.21-03-00961.2001 PMC6762322

[42] Stafford BK, Sher A, Litke AM, Feldheim DA. Spatial-Temporal Patterns of Retinal Waves Underlying Activity-Dependent Refinement of Retinofugal Projections. Neuron 2009; 64, 200–212 10.1016/j.neuron.2009.09.021 19874788 PMC2771121

[43] Maccione A, Hennig MH, Gandolfo M, Muthmann O, van Coppenhagen J, Eglen SJ, Berdondini L, Sernagor E. Following the ontogeny of retinal waves: pan-retinal recordings of population dynamics in the neonatal mouse. J. Physiol. 2014; 592, 1545–1563 10.1113/jphysiol.2013.262840 24366261 PMC3979611

[44] Butts DA, Kanold PO, Shatz CJ. A Burst-Based “Hebbian” Learning Rule at Retinogeniculate Synapses Links Retinal Waves to Activity-Dependent Refinement. Plos Biol. 2007; 5, e61 10.1371/journal.pbio.0050061 17341130 PMC1808114

[45] Olshausen BA, Field DJ. Sparse coding of sensory inputs. Current Opinion in Neurobiology 2004; 14, 481–487 10.1016/j.conb.2004.07.007 15321069

[46] Mastronarde DN. Correlated firing of retinal ganglion cells. Trends Neurosci. 1989; 12, 75–80 10.1016/0166-2236(89)90140-9 2469215

[47] Alonso JM, Usrey WM, Reid RC. Precisely correlated firing in cells of the lateral geniculate nucleus. Nature 1996; 383, 815–819 10.1038/383815a0 8893005

[48] Bair W, Zohary E, Newsome WT. Correlated firing in Macaque Visual Area MT: Time Scales and Relationship to Behavior. J. Neurosci. 2001; 21, 1676–1679 11222658 10.1523/JNEUROSCI.21-05-01676.2001 PMC6762960

[49] Kohn A, Smith MA. Stimulus Dependence of Neuronal Correlation in Primary Visual Cortex of the Macaque. J. Neurosci. 2005; 25, 3661–3673 10.1523/JNEUROSCI.5106-04.2005 15814797 PMC6725370

[50] Ford KJ, Félix AL, Feller MB. Cellular mechanisms underlying spatiotemporal features of cholinergic retinal waves. J. Neurosci. 2012; 32, 850–863 10.1523/JNEUROSCI.5309-12.2012 22262883 PMC3311224

[51] Li Y, van Hooser SD, Mazurek M, White LE, Fitzpatrick D. Experience with moving visual stimuli drives the early development of cortical direction selectivity. Nature 2008; 456, 952–956 10.1038/nature07417 18946471 PMC2644578

[52] Huberman AD, Feller MB, Chapman B. Mechanisms Underlying Development of Visual Maps and Receptive Fields. Annu. Rev. Neurosci. 2008; 31, 479–509 10.1146/annurev.neuro.31.060407.125533 18558864 PMC2655105

[53] Braastad BO, Heggelund P. Development of Spatial Receptive-Field Organization and Orientation Selectivity in Kitten Striate Cortex. J. Neurophysiol. 1985; 53, 1158–1178 3998804 10.1152/jn.1985.53.5.1158

[54] DeAngelis GC, Ohzawa I, Freeman RD. Spatiotemporal organization of simple-cell receptive fields in the cat’s striate cortex. I. General characteristics and postnatal development. J. Neurophysiol. 1993; 69, 1091–1117 8492151 10.1152/jn.1993.69.4.1091

[55] Tavazoie SH, Reid RC. Diverse receptive fields in the lateral geniculate nucleus during thalamocortical development. Nat. Neurosci. 2000; 3, 608–616 10.1038/75786 10816318

[56] Akerman CJ, Grubb MS, Thompson ID. Spatial and Temporal Properties of Visual Responses in the Thalamus of the Developing Ferret. J. Neurosci. 2004; 24, 170–182 10.1523/JNEUROSCI.1002-03.2004 14715950 PMC6729569

[57] Miller KD. A model for the development of simple cell receptive fields and the ordered arrangement of orientation columns through activity-dependent competition between ON- and OFF-center inputs. J. Neurosci. 1994; 14, 409–441 8283248 10.1523/JNEUROSCI.14-01-00409.1994 PMC6576834

[58] Feldman DE. The spike-timing dependence of plasticity. Neuron 2012; 75, 556–571 10.1016/j.neuron.2012.08.001 22920249 PMC3431193

[59] Goodhill GJ, Barrow HG. The Role of Weight Normalization in Competitive Learning. Neural Computation 1994; 6, 255–269 10.1162/neco.1994.6.2.255

[60] Miller KD. Synaptic Economics: Competition and Cooperation in Synaptic Plasticity. Neuron 1996; 17, 371–374 10.1016/S0896-6273(00)80169-5 8816700

[61] von der Malsburg C. Self-Organization of Orientation Sensitive Cells in the Striate Cortex. Kybernetik 1973; 14, 85–100 10.1007/BF00288907 4786750

[62] Wimbauer S, Wenisch OG, van Hemmen JL, Miller KD. Development of spatiotemporal receptive fields of simple cells: II. Simulation and analysis. Biol. Cybern. 1997; 77, 463–477 10.1007/s004220050406 9433757

[63] Zylberberg J, Murphy JT, DeWeese MR. A Sparse Coding Model with Synaptically Local Plasticity and Spiking Neurons Can Account for the Diverse Shapes of V1 Simple Cell Receptive Fields. PloS Comp. Biol. 2011; 7(10): e1002250 10.1371/journal.pcbi.1002250 PMC3203062 22046123

[64] Abbott LF, Nelson SB. Synaptic plasticity: taming the beast. Nat. Neurosci. Suppl. 2000; 3, 1178–1183 10.1038/81453 11127835

[65] Turrigiano GG, Nelson SB. Homeostatic plasticity in the developing nervous system. Nat. Rev. Neurosci. 2004; 5, 97–107 10.1038/nrn1327 14735113

[66] Nelson SB, Turrigiano GG. Strength through Diversity. Neuron 2008; 60, 477–482 10.1016/j.neuron.2008.10.020 18995822 PMC4919814

[67] Feller MB, Wellis DP, Stellwagen D, Werblin FS, Shatz CJ. Requirement for cholinergic synaptic transmission in the propagation of spontaneous retinal waves. Science 1996; 272, 1182–1187 10.1126/science.272.5265.1182 8638165

[68] Sernagor E, Eglen SJ, O’Donovan MJ. Differential Effects of Acetylcholine and Glutamate Blockade on the Spatiotemporal Dynamics of Retinal Waves. J. Neurosci. 2000; 20, RC56 (1–6) 10632622 10.1523/JNEUROSCI.20-02-j0004.2000 PMC6772391

[69] Sernagor E, Young C, Eglen SJ. Developmental Modulation of Retinal Wave Dynamics: Shedding Light on the GABA Saga. J. Neurosci. 2003; 23, 7621–7629 12930801 10.1523/JNEUROSCI.23-20-07621.2003 PMC6740765

[70] Syed MM, Lee S, Zheng J, Zhou ZJ. Stage-dependent dynamics and modulation of spontaneous waves in the developing rabbit retina. J. Physiol. 2004; 560, 533–549 10.1113/jphysiol.2004.066597 15308679 PMC1665265

[71] Warland DK, Huberman AD, Chalupa LM. Dynamics of Spontaneous Activity in the Fetal Macaque Retina during Development of Retinogeniculate Pathways. J. Neurosci. 2006; 26, 5190–5197 10.1523/JNEUROSCI.0328-06.2006 16687510 PMC6674245

[72] Sun C, Warland DK, Ballesteros JM, van der List D, Chalupa LM. Retinal waves in mice lacking the *β*2 subunit of the nicotinic acetylcholine receptor. Proc. Natl. Acad. Sci. USA 2008; 105, 13638–13643 10.1073/pnas.0807178105 18757739 PMC2527347

[73] McLaughlin T, Torborg CL, Feller MB, O’Leary DDM. Retinotopic map refinement requires spontaneous retinal waves during a brief critical period of development. Neuron 2003; 40, 1147–1160 10.1016/S0896-6273(03)00790-6 14687549

[74] Chandrasekaran AR, Plas DT, Gonzalez E, Crair MC. Evidence for an Instructive Role of Retinal Activity in Retinotopic Map Refinement in the Superior Colliculus of the Mouse. J. Neurosci. 2005; 25, 6929–6938 10.1523/JNEUROSCI.1470-05.2005 16033903 PMC6725341

[75] Guo Y, Huang S, de Pasquale R, McGehrin K, Lee HK, Zhao K, Kirkwood A. Dark Exposure Extends the Integration Window for Spike-Timing-Dependent Plasticity. J. Neurosci. 2012; 32, 15027–15035 10.1523/JNEUROSCI.2545-12.2012 23100424 PMC3496177

[76] Liu XB, Murray KD, Jones EG. Switching of NMDA Receptor 2A and 2B Subunits at Thalamic and Cortical Synapses during Early Postnatal Development. J. Neurosci. 2004; 24, 8885–8895 10.1523/JNEUROSCI.2476-04.2004 15470155 PMC6729956

[77] Shipton OA, Paulsen O. GluN2A and GluN2B subunit-containing NMDA receptors in hippocampal plasticity. Phil. Trans. R. Soc. B 2014; 369, 20130163 10.1098/rstb.2013.0163 24298164 PMC3843894

[78] Hubel DH, Wiesel TN. Receptive fields of cells in striate cortex of very young, visually inexperienced kittens. J. Neurophysiol. 1963; 26, 994–1002 14084171 10.1152/jn.1963.26.6.994

[79] Blakemore C, van Sluyters RC. Innate and environmental factors in the development of the kitten’s visual cortex. J. Physiol. 1975; 248, 663–716 10.1113/jphysiol.1975.sp010995 1151843 PMC1309546

[80] Sherk H, Stryker MP. Quantitative Study of Cortical Orientation Selectivity in Visually Inexperienced Kitten. J. Neurophysiol. 1976; 39, 63–70 1249604 10.1152/jn.1976.39.1.63

[81] White LE, Coppola DM, Fitzpatrick D. The contribution of sensory experience to the maturation of orientation selectivity in ferret visual cortex. Nature 2001; 411, 1049–1052 11429605 10.1038/35082568

[82] Blakemore C, Cooper GF. Development of the Brain depends on the Visual Environment. Nature 1970; 228, 477–478 10.1038/228477a0 5482506

[83] Blakemore C, Mitchell DE. Environmental Modification of the Visual Cortex and the Neural Basis of Learning and Memory. Nature 1973; 241, 467–468 10.1038/241467a0 4735865

[84] Stryker MP, Sherk H, Leventhal AG, Hirsch HVB. Physiological Consequences for the Cat’s Visual Cortex of Effectively Restricting Early Visual Experience With Oriented Contours. J. Neurophysiol. 1978; 41, 896–909 681993 10.1152/jn.1978.41.4.896

[85] Hubel DH, Wiesel TN. Receptive fields, binocular interaction and functional architecture in the cat’s visual cortex. J. Physiol. 1962; 160, 106–154 10.1113/jphysiol.1962.sp006837 14449617 PMC1359523

[86] Reid RC, Alonso JM. Specificity of monosynaptic connections from thalamus to visual cortex. Nature 1995; 378, 281–284 10.1038/378281a0 7477347

[87] Movshon JA, Thomson ID, Tolhurst DJ. Spatial summation in the receptive fields of simple cells in the cat’s striate cortex. J. Physiol. 1978; 283, 53–77 722589 10.1113/jphysiol.1978.sp012488 PMC1282765

[88] Ohshiro T, Weliky M. Simple fall-off pattern of correlated neural activity in the developing lateral geniculate nucleus. Nat. Neurosci. 2006; 9, 1541–1548 10.1038/nn1799 17115045

[89] Olshausen BA, Field DJ. Emergence of simple-cell receptive field properties by learning a sparse code for natural images. Nature 1996; 381, 607–609 10.1038/381607a0 8637596

[90] Bell AJ, Sejnowski TJ. The “Independent Components” of Natural Scenes are Edge Filters. Vision Res. 1997; 37, 3327–3338 10.1016/S0042-6989(97)00121-1 9425547 PMC2882863

[91] van Hateren JH, Ruderman DL. Independent component analysis of natural image sequences yields spatio-temporal filters similar to simple cells in primary visual cortex. Proc. R. Soc. Lond. B 1998; 265, 2315–2320 10.1098/rspb.1998.0577 PMC1689525 9881476

[92] van Hooser SD, Li Y, Christensson M, Smith GB, White LE, Fitzpatrick D. Initial neighborhood biases and the quality of motion stimulation jointly influence the rapid emergence of direction preference in visual cortex. J. Neurosci. 2012; 32, 7258–7266 10.1523/JNEUROSCI.0230-12.2012 22623671 PMC3368384

[93] Movshon JA, Thompson ID, Tolhurst DJ. Spatial and temporal contrast sensitivity of neurones in areas 17 and 18 of the cat’s visual cortex. J. Physiol. 1978; 283, 101–120 10.1113/jphysiol.1978.sp012490 722570 PMC1282767

[94] Movshon JA. The velocity tuning of single units in cat striate cortex. J. Physiol. 1975; 249, 445–468 10.1113/jphysiol.1975.sp011025 1177101 PMC1309587

[95] Froemke RC, Poo MM, Dan Y. Spike-timing-dependent synaptic plasticity depends on dendritic location. Nature 2005; 434, 221–225 10.1038/nature03366 15759002

[96] Hubel DH, Wiesel TN. Receptive fields, binocular interaction and functional architecture in the cat’s visual cortex. J. Physiol. 1962; 160, 106–154 10.1113/jphysiol.1962.sp006837 14449617 PMC1359523

[97] Adelson EH, Bergen JR. Spatiotemporal energy models for the perception of motion. J. Opt. Soc. Am. A 1985; 2, 284–299 10.1364/JOSAA.2.000284 3973762

[98] Saul AB, Humphrey AL. Spatial and temporal response properties of lagged and nonlagged cells in cat lateral geniculate nucleus. J. Neurophysiol. 1990; 64, 206–224 2388066 10.1152/jn.1990.64.1.206

[99] Wolfe J, Palmer LA. Temporal diversity in the lateral geniculate nucleus of cat. Visual Neurosci. 1998; 15, 653–675 10.1017/S0952523898154068 9682868

[100] De Valois RL, Cottaris NP, Mahon LE, Elfar SD, Wilson JA. Spatial and temporal receptive fields of geniculate and cortical cells and directional selectivity. Vision Research 2000; 40, 3685–3702 10.1016/S0042-6989(00)00210-8 11090662

[101] Mooney W, Penn AA, Gallego R, Shatz CJ. Thalamic relay of spontaneous retinal activity prior to vision. Neuron 1996; 17, 979–990 10.1016/S0896-6273(00)80218-4 8938119

[102] Akerman CJ, Smyth D, Thompson ID. Visual Experience before Eye-Opening and the Development of the Retinogeniculate Pathway. Neuron 2002; 36, 869–879 10.1016/S0896-6273(02)01010-3 12467590

[103] Fiser J, Chiu C, Weliky M. Small modulation of ongoing cortical dynamics by sensory input during natural vision. Nature 2004; 431, 573–578 10.1038/nature02907 15457262

[104] Shouval HZ, Bear MF, Cooper LN. A unified model of NMDA receptor-dependent bidirectional synaptic plasticity. Proc. Natl. Acad. Sci. USA 2002; 99, 10831–10836 10.1073/pnas.152343099 12136127 PMC125058

[105] Graupner M, Brunel N. Calcium-based plasticity model explains sensitivity of synaptic changes to spike pattern, rate, and dendritic location. Proc. Natl. Acad. Sci. USA 2012; 109, 3991–3996 10.1073/pnas.1109359109 22357758 PMC3309784

[106] Kobayashi K, Poo MM. Spike train timing-dependent associative modification of hippocampal CA3 recurrent synapses by mossy fibers. Neuron 2004; 41, 445–454 10.1016/S0896-6273(03)00873-0 14766182

[107] Nevian T, Sakmann B. Spine Ca^2+^ Signaling in Spike-Timing-Dependent Plasticity. J. Neurosci. 2006; 26, 11001–11013 10.1523/JNEUROSCI.1749-06.2006 17065442 PMC6674669

[108] Enroth-Cugell C, Robson JG. The contrast sensitivity of retinal ganglion cells of the cat. J. Physiol. 1966; 187, 517–552 10.1113/jphysiol.1966.sp008107 16783910 PMC1395960

[109] Enroth-Cugell C, Robson JG, Schweitzer-Tong DE, Watson AB. Spatio-temporal interactions in cat retinal ganglion cells showing linear spatial summation. J. Physiol. 1983; 341, 279–307 10.1113/jphysiol.1983.sp014806 6620181 PMC1195335

[110] Hubel DH, Wiesel TN. Integrative action in the cat’s lateral geniculate body. J. Physiol. 1961; 155, 385–396 10.1113/jphysiol.1961.sp006635 13716436 PMC1359861

[111] Cai D, DeAngelis GC, Freeman RD. Spatiotemporal Receptive Field Organization in the Lateral Geniculate Nucleus of Cats and Kittens. J. Neurophysiol. 1997; 78, 1045–1061 9307134 10.1152/jn.1997.78.2.1045

[112] Pastalkova E, Istkov V, Amarasingham A, Buszaki G. Internally Generated Cell Assembly Sequences in the Rat Hippocampus. Science 2008; 321, 1322–1327 10.1126/science.1159775 18772431 PMC2570043

[113] Hahnloser RHR, Kozhevnikov AA, Fee MS. An ultra-sparse code underlies the generation of neural sequences in a songbird. Nature 2002; 419, 65–70 10.1038/nature00974 12214232

[114] Ikegaya Y, Aaron G, Cossart R, Aronov D, Lampl I, Ferster D, Yuste R. Synfire Chains and Cortical Songs: Temporal Modules of Cortical Activity. Science 2004; 304, 559–564 10.1126/science.1093173 15105494

[115] Butts DA, Feller MB, Shatz CJ, Rokhsar DS. Retinal waves are governed by collective network properties. J. Neurosci. 1999; 19, 3580–3593 10212317 10.1523/JNEUROSCI.19-09-03580.1999 PMC6782231

[116] Sterratt DC, Lyngholm D, Willshaw DJ, Thompson ID. Standard Anatomical and Visual Space for the Mouse Retina: Computational Reconstruction and Transformation of Flattened Retinae with the Retistruct Package. PLOS Comp. Biol. 2013; 9(2): e1002921 10.1371/journal.pcbi.1002921 PMC3585388 23468609

[117] Bodenstein L, Sidman RL. Growth and Development of the Mouse Retinal Pigment Epithelium. Develop. Biol. 1987; 121, 192–204 10.1016/0012-1606(87)90153-9 3569658

[118] Henderson Z, Finlay BL, Wikler KC. Development of Ganglion Cell Topography in Ferret Retina. J. Neurosci. 1986; 8, 1194–1205 10.1523/JNEUROSCI.08-04-01194.1988 PMC6569276 3357016

[119] Thorn F, Gollender M, Erickson P. The development of the kitten’s visual optics. Vision Res. 1975; 16, 1145–1149 10.1016/0042-6989(76)90255-8 969227

