# Supplementary material for: Refinement and Pattern Formation in Neural Circuits by the Interaction of Traveling Waves with Spike-Timing Dependent Plasticity
Source: PLoS Comput Biol. 2015 Aug 26;11(8):e1004422. doi: 10.1371/journal.pcbi.1004422 (PMC4550436; doi:10.1371/journal.pcbi.1004422)
Supplement: S1 Text — (PDF) [file pcbi.1004422.s002.pdf]

S1 Text

Full derivation of Eq. 5 in the main text

Here, we provide a full derivation of the synaptic strength dynamics in Eq. 5 in the main text, taking into consideration 1) the time dependent firing rate of input neurons when recruited by a wave, and 2) the excitatory postsynaptic potentials (EPSPs) elicited in the output neuron. These additional terms do not compromise the assumptions required to derive Eq. 3, so we pick up the derivation at this point. The input firing rate and EPSPs enter the derivation in the calculation of the correlation function:

$$C(x,\Delta t) = \int_{-\infty}^{\infty} dt\, S_{\text{in}}(x, t+\Delta t) S_{\text{out}}(t), \tag{S1}$$

where $S_{\text{in}}(x,t)$ and $S_{\text{out}}(t)$ are the input and output firing rates, respectively, as an isolated wave traverses the input layer. The wavefront in the input layer is still described by a traveling pulse, $\delta(x - vt)$, as in the main text, and the firing rate of a single input neuron when recruited by the wavefront is described by $\alpha(t)$, which is zero valued for $t < 0$. The input firing rate during a wave is the convolution in time of the traveling pulse with $\alpha(t)$:

$$\begin{aligned} S_{\text{in}}(x,t) &= \int_{-\infty}^{\infty} dt'\, \delta(x - vt')\alpha(t-t') \\ &= \alpha(t - x/v), \end{aligned} \tag{S2}$$

and the output firing rate is the convolution in time of the input firing rate with the EPSP, $\epsilon(t)$, which is also zero valued for $t < 0$:

$$\begin{aligned} S_{\text{out}}(t) &= \int_{-\infty}^{\infty} dx\, w(x) \int_{-\infty}^{\infty} dt' \int_{-\infty}^{\infty} dt''\, \delta(x - vt'')\alpha(t'-t'')\epsilon(t-t'-t'') \\ &= \int_{-\infty}^{\infty} dx\, w(x) \int_{-\infty}^{\infty} dt'\, \alpha(t' - x/v)\epsilon(t - t' - x/v) \\ &= \int_{-\infty}^{\infty} dx\, w(x)[\alpha(t - x/v) * \epsilon(t - x/v)] \\ &= w(vt) * \alpha(t) * \epsilon(t). \end{aligned} \tag{S3}$$

where $*$ denotes convolution. Substituting Eqs. S2 and S3 into Eq. S1, the correlation function can be found thus:

$$\begin{aligned} C(x,\Delta t) &= \int_{-\infty}^{\infty} dt\, \alpha(t + \Delta t - x/v)[w(vt) * \alpha(t) * \epsilon(t)] \\ &= \alpha(\Delta t - x/v) * \alpha(x/v - \Delta t) * \epsilon(x/v - \Delta t) * w(x - v\Delta t). \end{aligned} \tag{S4}$$

The synaptic strength dynamics can now be computed by inserting Eq. S4 into Eq. 3:

$$\begin{aligned} \partial_T w(x) &= \eta \int_{-\infty}^{\infty} d\Delta t\, K(\Delta t) C(x, \Delta t, T) \\ &= \eta \int_{-\infty}^{\infty} d\Delta t\, K(\Delta t)[\alpha(\Delta t - x/v) * \alpha(x/v - \Delta t) * \epsilon(x/v - \Delta t) * w(x - v\Delta t)] \\ &= \frac{\eta}{v} \int_{-\infty}^{\infty} d\Delta x\, K(\Delta x/v)[\alpha((\Delta x - x)/v) * \alpha((x - \Delta x)/v) * \epsilon((x - \Delta x)/v) * w(x - \Delta x)] \\ &= \eta K_v(x) * \alpha(-x/v) * \alpha(x/v) * \epsilon(x/v) * w(x), \end{aligned} \tag{S5}$$

where $K_v(x) = v^{-1}K(x/v)$, as we had done in the main text. Here, we have arrived at the result expressed in Eq. 5. We go on to find solutions for this expression of the synaptic strength dynamics in the main text.
