# Supplementary figures and images for: Refinement and Pattern Formation in Neural Circuits by the Interaction of Traveling Waves with Spike-Timing Dependent Plasticity
Source: PLoS Comput Biol. 2015 Aug 26;11(8):e1004422. doi: 10.1371/journal.pcbi.1004422 (PMC4550436; doi:10.1371/journal.pcbi.1004422)

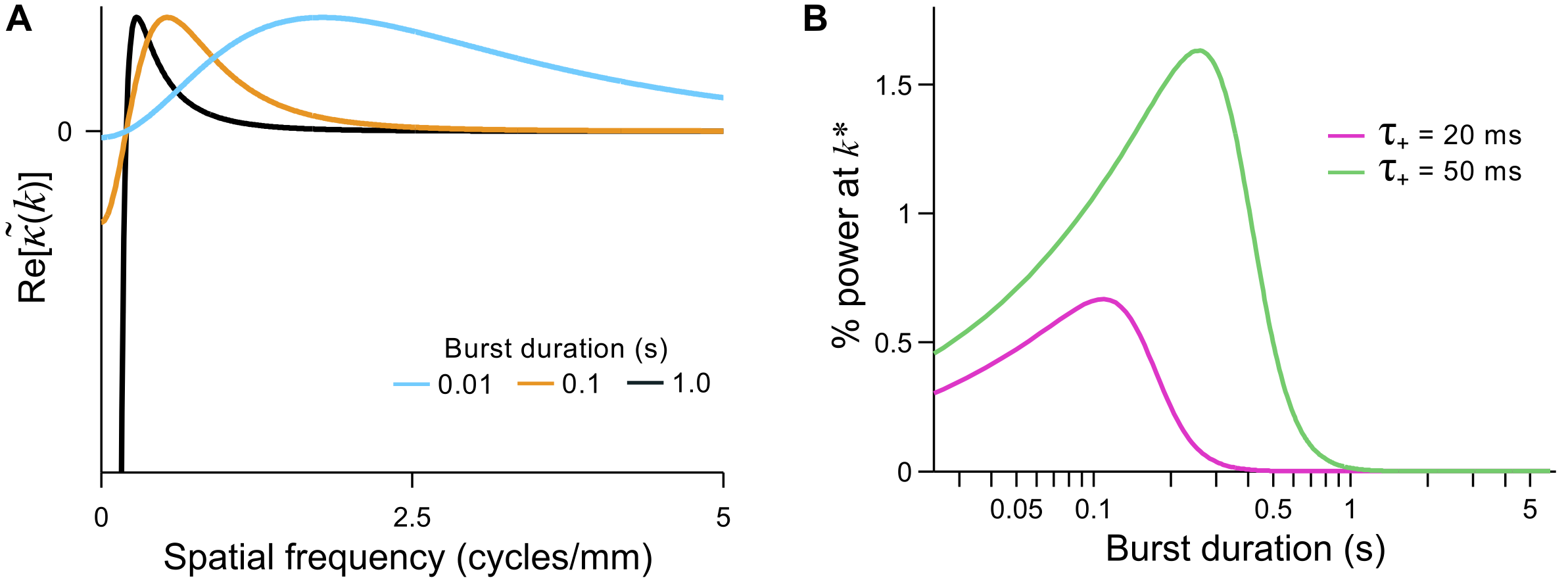

Supplement: S1 Fig — All other parameters are the same as for Fig 4D. The power is distributed across a broad range of high frequencies for a 0.01 s burst, and within a small band of the lowest frequencies for a 1.0 s burst, yielding the strong negative lobe in the black curve. The negative lobe in the black curve extends beyond the horizontal axis and has been cut for clarity. However, for 0.1 s bursts, the power is concentrated in between these two extremes, around the dominant spatial frequency, k*. (TIFF) [file pcbi.1004422.s003.tiff]

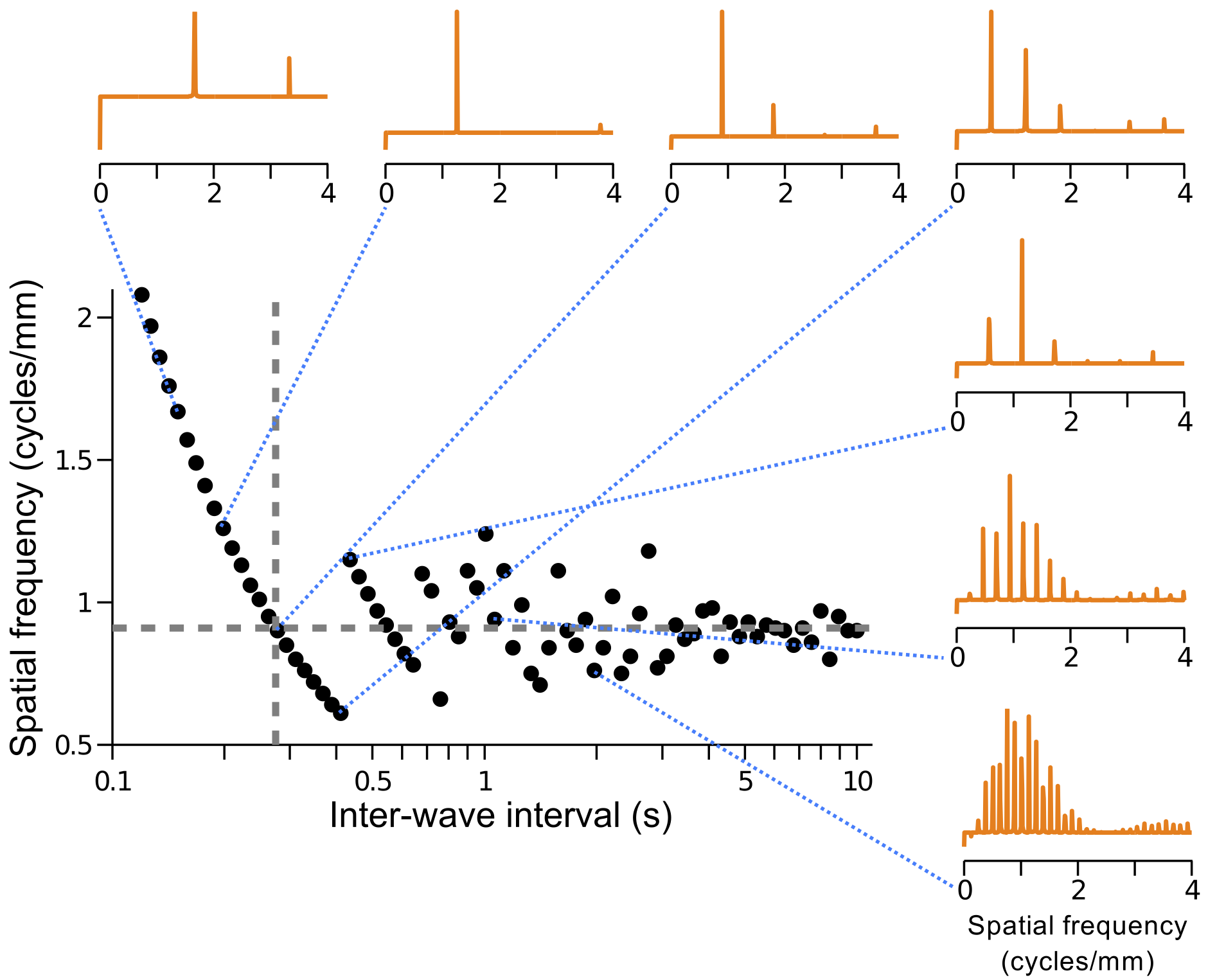

Supplement: S2 Fig — Black circles: predicted spatial frequencies. Horizontal dashed grey line: predicted spatial frequency for a single wave in isolation. Vertical dashed grey line: critical IWI, IWIcrit = 1/vk*. Insets: examples of Reκ~(k) for different IWIs, including IWIs of 0.15 s (top left) and 0.2 s (second from top left). The dominant spatial frequency for regular waves varies around ∼ 0.91 cycles/mm, which is the dominant frequency for an isolated wave, as a result of different peaks in α˜III(vk) being picked out by K~v(k). When the IWI falls below IWIcrit, the dominant frequency increases monotonically with decreasing IWI. (TIFF) [file pcbi.1004422.s004.tiff]

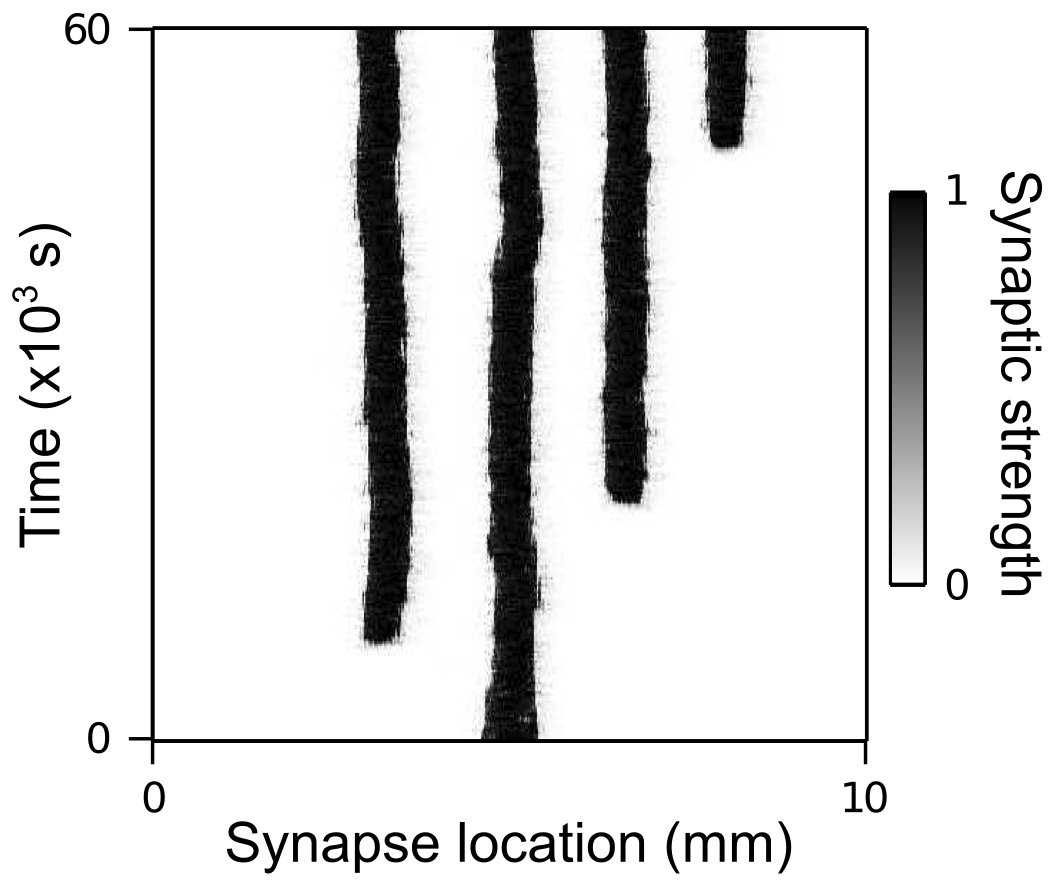

Supplement: S3 Fig — (TIFF) [file pcbi.1004422.s005.tiff]

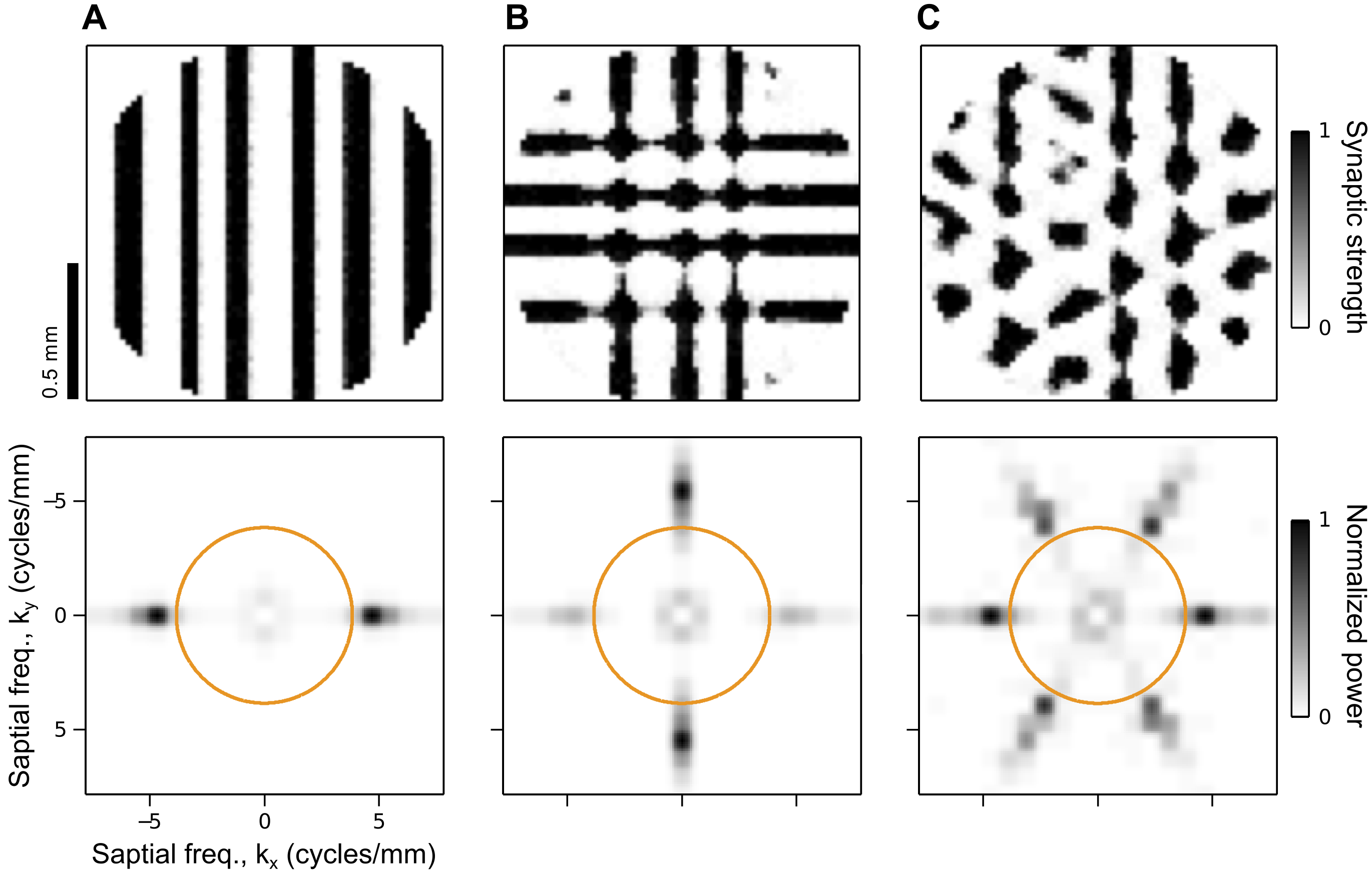

Supplement: S4 Fig — Top row: example RFs using wave speeds of 1 mm/s and τ + = 20 ms. An arbor of 0.66 mm was used. Bottom row: 2D power spectra of the RFs with DC component removed, averaged over four repeated trials of the simulation, and normalized to the peak power. The orange circle denotes the predicted dominant spatial frequency for waves traveling in all directions. A. Waves traveling along the horizontal axis yield RFs that exhibit vertically aligned subfields and that are indicative of simple cell RFs in primary visual cortex. B. Waves travel along the horizontal and vertical axes. C. Waves travel along three equally spaced axes. (TIFF) [file pcbi.1004422.s006.tiff]

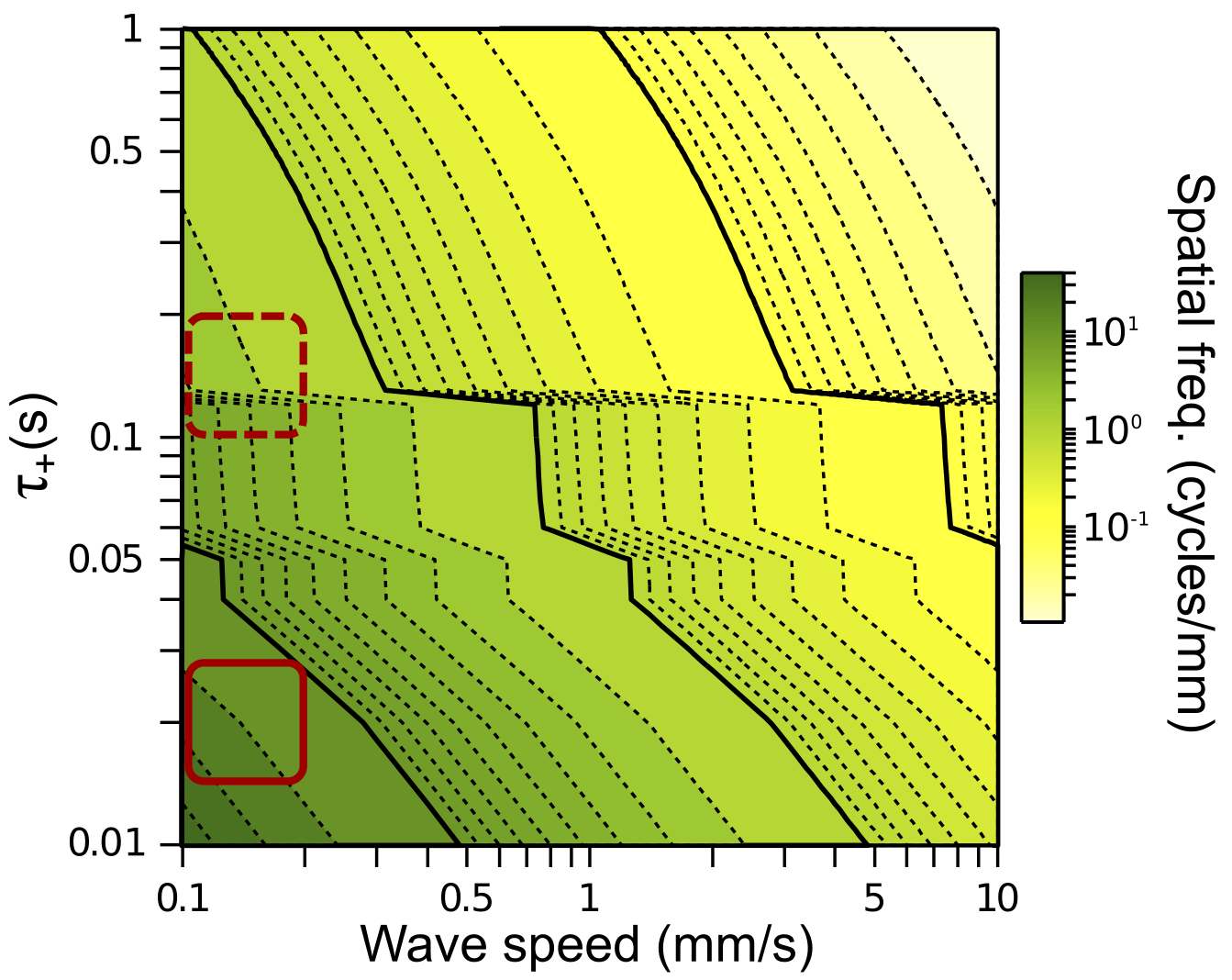

Supplement: S5 Fig — Solid black contours denote spatial frequencies equal to 10 raised to integer exponents. Spatial frequencies were obtained by locating the maximum in κ~(k) as a function of v and τ +, using an asymmetric STDP rule and a burst duration of 2 s for α(t). Sharp transitions in spatial frequency along the τ + axis are due to κ(x) having several peaks of near equal amplitude (c.f. black curve in Fig 4D), such that small changes in τ + can change which peak is the global maximum. Solid red rectangle: given a typical STDP rule with τ + = 20 ms, the connectivity pattern associated with retinal wave speeds would have a dominant spatial frequency of ∼ 11 cycles/mm, or a wavelength of 0.9 mm. Dashed red rectangle: RFs in the SC require a characteristic wavelength of ∼ 0.51 mm, which corresponds to a spatial frequency of ∼ 2 cycles/mm. Given the speed of retinal waves, the required STDP time scale is predicted to be 0.1–0.2 s. (TIFF) [file pcbi.1004422.s007.tiff]

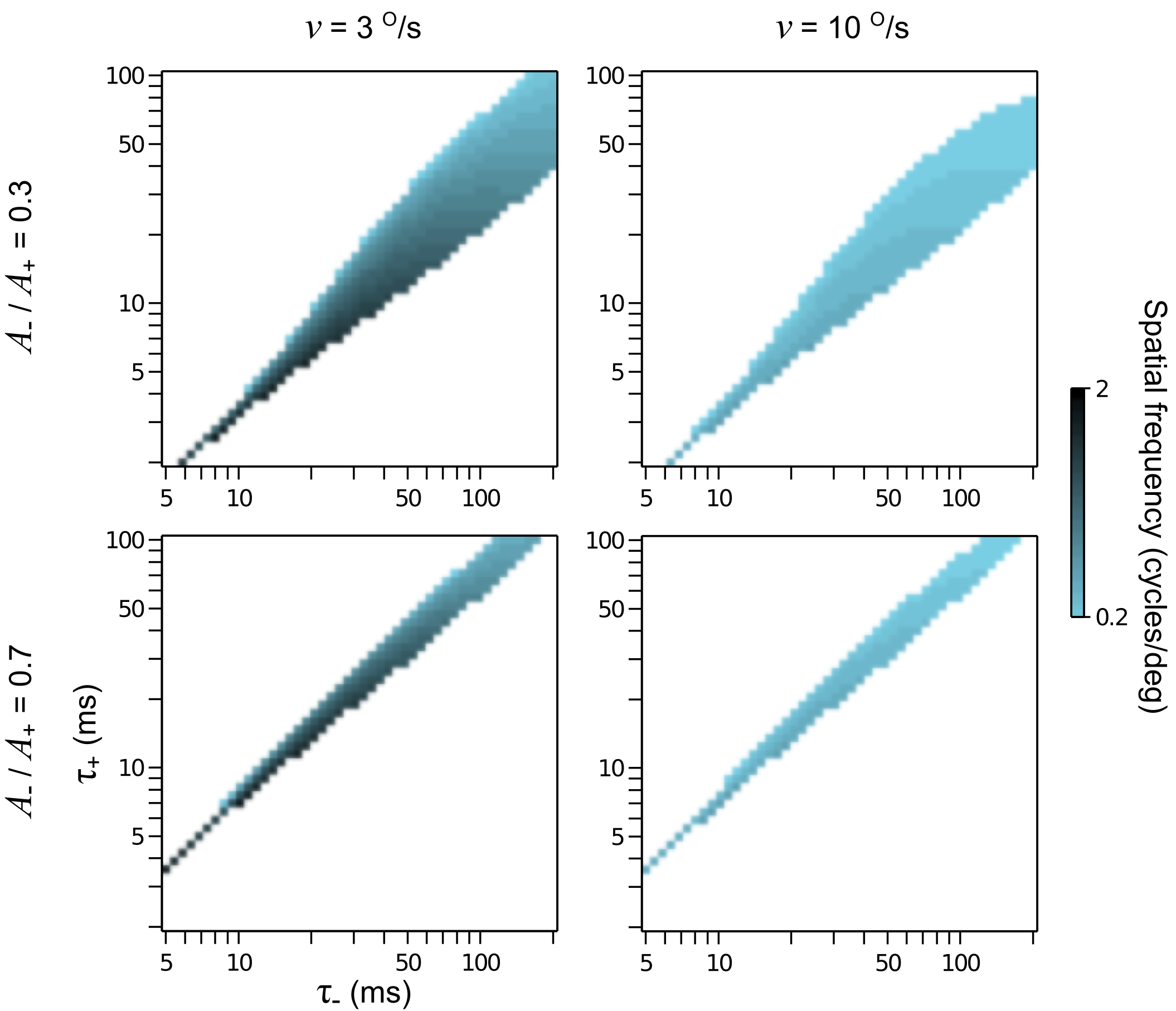

Supplement: S6 Fig — Using a burst duration of 100 ms, which matches the impulse response duration of immature LGN cells in the kitten [111], we computed a spatial frequency map as a function of the STDP decay times, τ + and τ -, for four conditions: the STDP amplitudes were either A −/A + = 0.3 (top row) or A +/A − = 0.7 (bottom row), and the wave speed was either 3°/s (left column) or 10°/s (right column) [94]. We further restricted our analysis to STDP rules that had a reasonable bias for either weakening or strengthening by ignoring any rule for which the DC power exceeded that at the dominant spatial frequency, i.e. cases when |κ~(0)|2>|κ~(k*)|2. We also restrict the spatial frequency maps to frequencies that lie in the range observed in adult cats: 0.2–2 cycles/° [54, 93]. (TIFF) [file pcbi.1004422.s008.tiff]

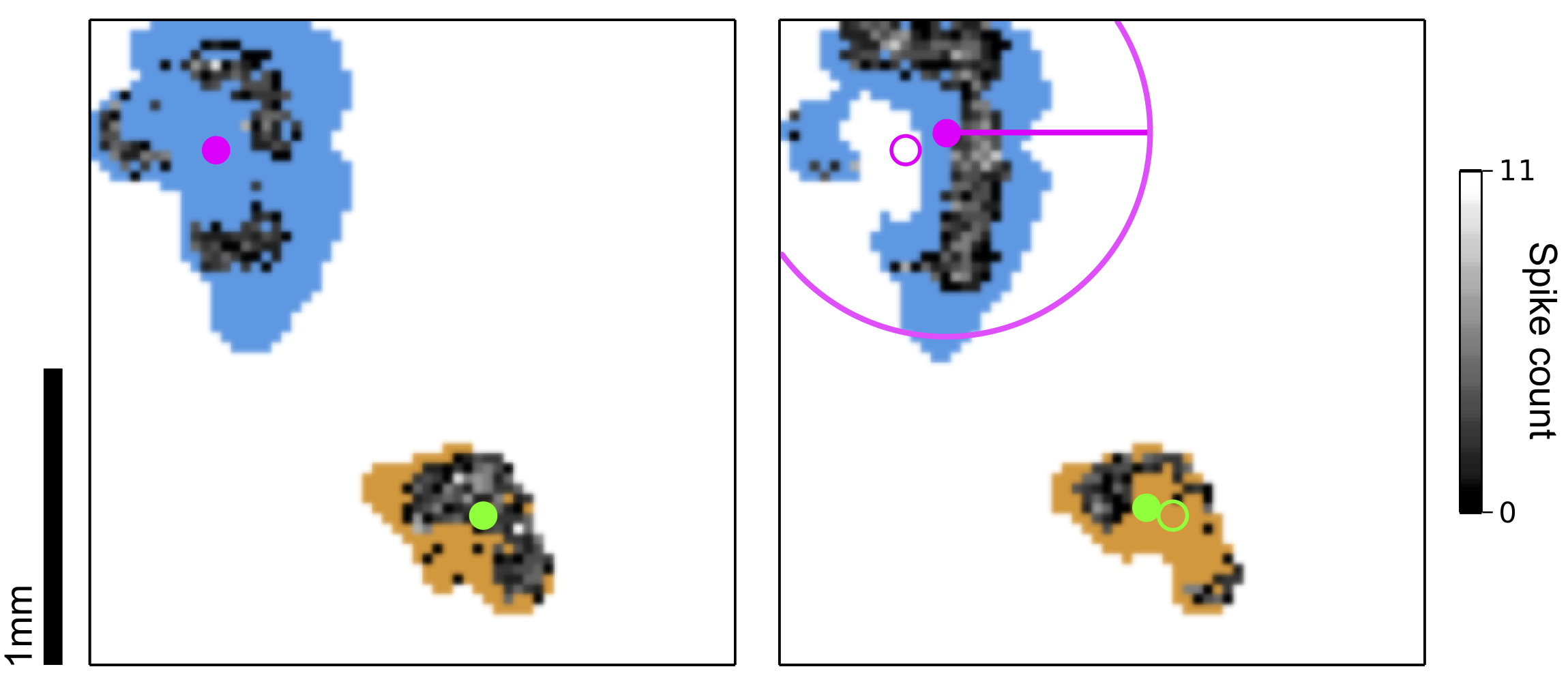

Supplement: S7 Fig — The two images depict activity that was generated with the complex wave model at time bins k (left) and k + 1 (right). The solid blue areas mark the domains in M s(x, y, t) that were assigned to one isolated wave, and the solid orange areas mark domains that were assigned to another isolated wave. Greyscale pixels illustrate the firing rates of RGCs in M(x, y, t) within each domain. Solid purple and green dots denote the COM for the first and second wave, respectively, in the current time bin. To illustrate how the COMs moved between time bins, the open purple and green dots (right) denote the COM of each wave in the previous time bin (left). Domains with COMs that are separated by less than 680 μm (purple circle around the blue wave), in the same time bin or in adjacent time bins, are assigned to the same wave. (TIFF) [file pcbi.1004422.s009.tiff]
